# *In silico* modelling of multi‐electrode arrays for enhancing cardiac drug testing on hiPSC‐CM heterogeneous tissues

**DOI:** 10.1113/JP287276

**Published:** 2025-05-11

**Authors:** Sofia Botti, Rolf Krause, Luca F. Pavarino

**Affiliations:** ^1^ Euler Institute Università della Svizzera Italiana Lugano Switzerland; ^2^ Department of Mathematics “Felice Casorati” University of Pavia Pavia Italy; ^3^ Faculty of Mathematics and Informatics, UniDistance Brig Switzerland; ^4^ AMCS, CEMSE, King Abdullah University of Science and Technology Thuwal Kingdom of Saudi Arabia

**Keywords:** cardiac drug testing, computational cardiology, human‐induced pluripotent stem cell‐derived cardiomyocytes, mathematical models, multi‐electrode array systems

## Abstract

**Abstract:**

Human‐induced pluripotent stem cell‐derived cardiomyocytes (hiPSC‐CMs) offer a transformative platform for *in vitro* and *in silico* testing of patient‐specific drugs, enabling detailed study of cardiac electrophysiology. By integrating standard experimental techniques with extracellular potential measurements from multi‐electrode arrays (MEAs), researchers can capture key tissue‐level electrophysiological properties, such as action potential dynamics and conduction characteristics. This study presents an innovative computational framework that combines an MEA‐based electrophysiological model with phenotype‐specific hiPSC‐CM ionic models, enabling accurate *in silico* predictions of drug responses. We tested four drug compounds and ion channel blockers using this model and compared these predictions against experimental MEA data, establishing the model's robustness and reliability. Additionally, we examined how tissue heterogeneity in hiPSC‐CMs affects conduction velocity, providing insights into how cellular variations can influence drug efficacy and tissue‐level electrical behaviour. Our model was further tested through simulations of Brugada syndrome, successfully replicating pathological electrophysiological patterns observed in adult cardiac tissues. These findings highlight the potential of hiPSC‐CM MEA‐based *in silico* modelling for advancing drug screening processes, which have the potential to refine disease‐specific therapy development, and improve patient outcomes in complex cardiac conditions.

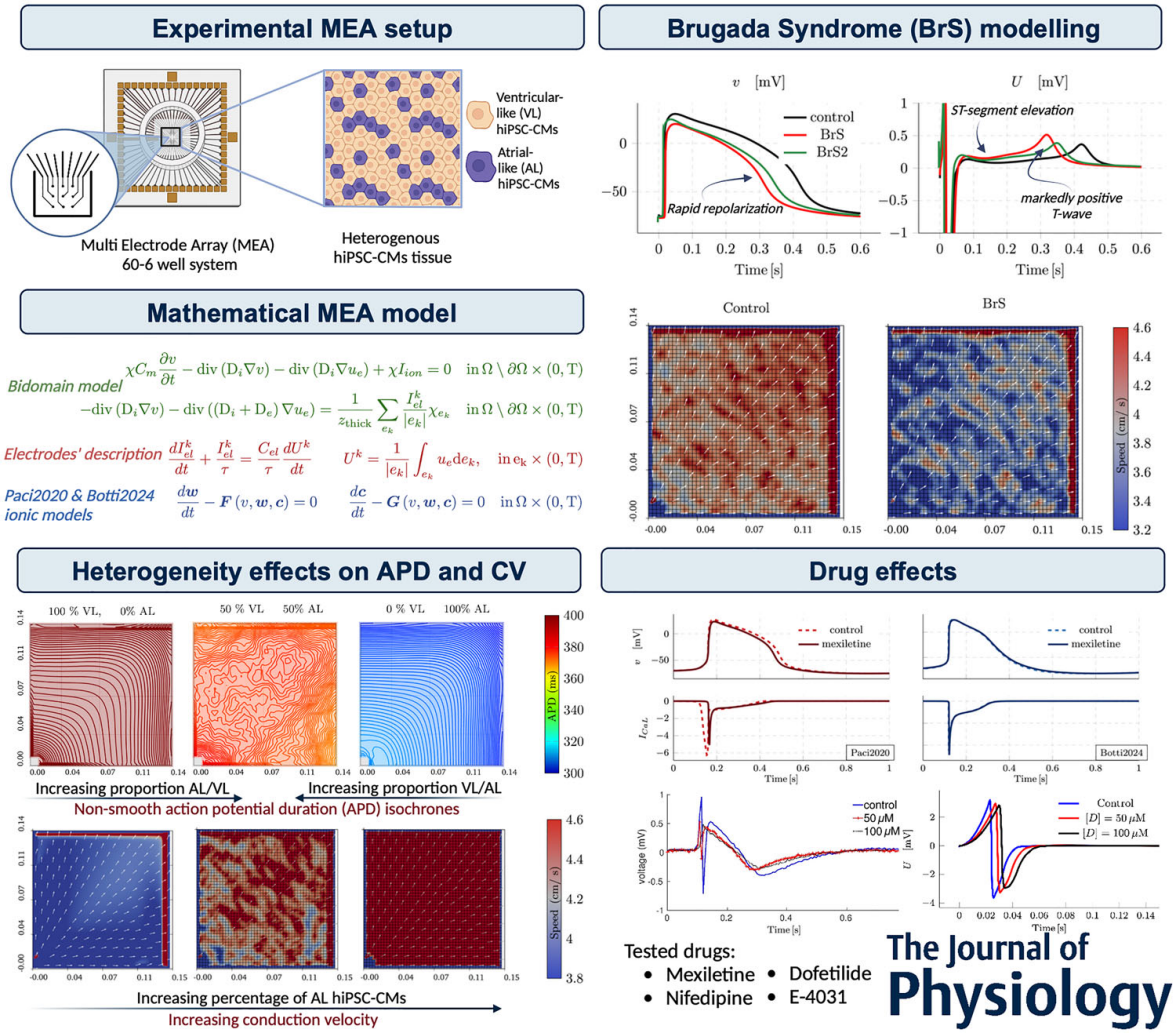

**Key points:**

- Human‐induced pluripotent stem cell‐derived cardiomyocytes (hiPSC‐CMs) offer a transformative platform for *in vitro* and *in silico* testing of patient‐specific drugs, enabling detailed study of cardiac electrophysiology.
- Development of an innovative computational framework that combines a multi‐electrode array (MEA)‐based electrophysiological model with phenotype‐specific hiPSC‐CM ionic models.
- Drug testing of four compounds and ion channel blockers using this hiPSC‐CM MEA model and comparison against experimental MEA data, establishing the model's robustness and reliability.
- Study of the effect of tissue heterogeneity in hiPSC‐CMs on conduction velocity, providing insights into how cellular variations can influence drug efficacy and tissue‐level electrical behaviour.
- Brugada syndrome simulation through the hiPSC‐CM MEA model, successfully replicating pathological electrophysiological patterns observed in adult cardiac tissues.

## Graphical abstract

**Abstract figure legend** Schematic overview of the experimental and computational framework for investigating hiPSC‐CM electrophysiology with MEA systems. The MEA‐based model integrates experimental data with phenotype‐specific ionic models and tissue‐level heterogeneity. Colour‐coded maps highlight the impact of heterogeneity on conduction velocity, which increases with a higher percentage of atrial‐like (AL) cells. Brugada syndrome simulations reveal locally reduced conduction velocity, consistent with adult tissue observations. Drug simulations for mexiletine, dofetilide, nifedipine and E‐4031 demonstrate the model's capability to predict extracellular potential changes and ionic current dynamics. This integrative framework offers a comprehensive approach to assess disease‐specific and pharmacological electrophysiological responses.

## Introduction

Human induced pluripotent stem cell‐derived cardiomyocytes (hiPSC‐CMs) have become an essential tool for drug testing and cardiotoxicity screening due to their ability to replicate key electrophysiological properties of native cardiomyocytes (Takahashi & Yamanaka, 2006; Takahashi et al., 2007). They provide a scalable, human‐relevant platform for high‐throughput pharmacological studies, offering advantages over traditional animal models (Bekhite & Schulze, 2021). Multi‐electrode array (MEA) technology plays a central role in hiPSC‐CM‐based drug screening by enabling non‐invasive, real‐time recording of extracellular electrical signals. This well‐established method provides insights into cellular electrophysiology and drug responses, making it a gold standard for evaluating compound effects (Pickard, 1979; Spira & Hai, 2013). However, challenges such as hiPSC‐CM immaturity and cellular heterogeneity hinder their predictive power, requiring advances in differentiation protocols and culture conditions (Cerneckis et al., 2024). The integration of hiPSC‐CM monolayer cultures with MEA systems enables accurate, real‐time assessment of electrophysiological properties, supporting applications in drug discovery, disease modelling and personalized medicine (Zhu et al., 2017; Raphel et al., 2017). MEA‐recorded field potentials (FPs) indirectly reflect action potentials (APs), capturing depolarization and repolarization phases. However, FP analysis is complicated by signal variability arising from factors such as cell density, culture conditions, electrode placement and hiPSC‐CM heterogeneity (Clements & Thomas, 2014).

From a computational perspective, existing hiPSC‐CM tissue simulations rely on the bidomain model but lack MEA device‐specific features and phenotypical heterogeneity. At the single‐cell level, patch‐clamp measurements allow for the classification of hiPSC‐CMs into atrial‐like (AL), ventricular‐like (VL) and nodal‐like subtypes based on AP characteristics (Lodrini et al., 2020; Chapotte‐Baldacci et al., 2024, 2023). Recent ionic models, such as Paci2020 (Paci et al., 2020), improve the representation of VL phenotypes, while the Botti2024 model (Botti et al., 2024), based on dynamic clamp recordings, advances AL modelling by incorporating atrial‐specific currents. This study extends the bidomain model with a novel electrode–tissue coupling framework tailored for MEA devices. A key contribution of this work is the incorporation of phenotypical heterogeneity into hiPSC‐CM tissue models, allowing for realistic simulations of diverse cellular responses. This advancement improves the predictive accuracy of *in silico* studies for drug screening and disease modelling. Additionally, we simulate the differential electrophysiological manifestations of Brugada syndrome in stem cell‐derived cardiac tissues, highlighting parallels with adult cardiac tissue responses. Finally, we evaluate the effects of four pharmacological compounds and validate the model against experimental MEA data from Abbate et al. (2018) and Millard et al. (2018), demonstrating the model's applicability in preclinical drug testing.

The remainder of this paper is structured as follows: the next section outlines the models and numerical methods, providing some useful definition. The subsequent section presents key results, including an activation and repolarization maps, Brugada syndrome effects, and the effects of four current blockers: mexiletine, dofetilide, nifedipine and E‐4031. These findings are then discussed, followed by concluding remarks.

## Methods

Our primary objective is to develop a robust model that accurately captures the realistic behaviour, complexity and heterogeneity of a MEA system. In this section, we introduce a mathematical model tailored to address tissue electrophysiology while incorporating the effects of micro‐electrodes. We also address the immature characteristics of hiPSC‐CM cultures, including the resulting heterogeneity in the phenotype distribution, and we briefly outline the numerical scheme employed to discretize the system in time and space.

### Mathematical model

In cardiac electrophysiology modelling, the electrical behaviour of cardiac tissue in space (x∈Ω) and time (t∈(0,T)) is described by means of two superimposed continua, the intra‐ and the extra‐cellular spaces. These compartments exchange charge through the ionic current $I_{\mathrm{ion}}$ across the cellular membrane, the intensity of which depends in a point‐wise manner on the difference between the intracellular potential $u_{i}$ and the extracellular potential $u_{e}$

(1) $$v\;\left(x,t\right)=u_{i}\;\left(x,t\right)-u_{e}\left(x,t\right).$$

Within a single compartment, the charge redistributes according to the linear diffusion law governed by the conductivity tensors *D*_i_ and *D*_e_ for the intracellular and extracellular spaces, respectively, linking the local cellular state to that of neighbouring cells.

Moreover, the charge flux through the cellular membrane depends on the capacitance $C_{m}$ and ratio χ – between the membrane surface area and the volume enclosed by this surface – regulating the time delay, while the current $I_{\mathrm{app}}$ represents an external stimulation applied at a specific location within the domain for a defined duration. The charge conservation law finally leads to the bidomain formulation. For a detailed derivation of the bidomain model see Colli Franzone et al. (2014) and Sundnes et al. (2007).

The standard mathematical model is then coupled with a source term which describes the electrode behaviour as in Abbate et al. (2018). In a three‐dimensional description, we denote with $e_{k}$ the $k^{\mathrm{th}}$ electrode location, while electric current, denoted as $I_{\mathrm{el}}^{k}$, recorded by the $k^{\mathrm{th}}$ electrode, is calculated by solving the following ordinary differential equation (ODE):

(2) $$\frac{dI_{\mathrm{el}}^{k}}{\mathrm{dt}}+\frac{I_{\mathrm{el}}^{k}}{\tau }=\frac{C_{\mathrm{el}}}{\tau }\;\frac{dU^{k}}{\mathrm{dt}}\;\mathrm{with}\;\;U^{k}=\frac{1}{\left|e_{k}\right|}\;\int_{e_{k}}u_{e}de_{k}.$$

Here, we define $\tau =\left(R_{i}+R_{e}\right)\;C_{\mathrm{el}}$, where $R_{i}$ represents the resistance to ground, while $R_{\mathrm{el}}$ and $C_{\mathrm{el}}$ denote the resistance and capacitance associated with the electrode. In Eq. (2), we also introduce $U^{k}$ as the average value of the extracellular potential $u_{e}$ on the electrode k.

Moreover, the subsequent boundary condition must be applied in the areas where the electrodes are situated, as noted by Moulin et al. (2008):

(3) $$D_{e}\nabla u_{e}\cdot n=\frac{I_{\mathrm{el}}^{k}}{\left|e_{k}\right|}\;\;\mathrm{on}\;\;e_{k}.$$

Given that the thickness of the cell monolayer $z_{\mathrm{thick}}$ is assumed to be negligible relative to the other dimensions in the problem, we can treat all variations in the z‐direction as insignificant when compared to those in the *x* and *y* directions. Following some algebraic manipulations, Eq. (3) is reformulated as a source term within the parabolic–elliptic (PE) bidomain framework. The FP measured by electrode *k* is then determined as

(4) $$U_{\mathrm{FP}}^{k}=R_{i}\;I_{\mathrm{el}}^{k}$$

In a two‐dimensional configuration of the MEA, the set of equations of the MEA model in the PE formulation reads as:


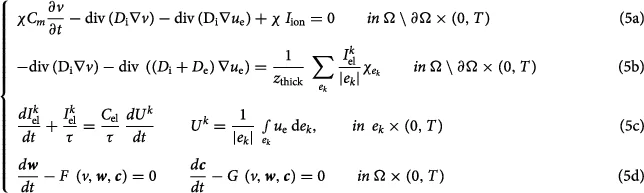

where $|e_{k}|$ denotes the $k^{\mathrm{th}}$ electrode's surface, and $\chi_{e_{k}}$ its characteristic function (equal to one inside the electrode and zero outside). Note that this MEA bidomain model of electrode recordings differs from organ‐level bidomain settings where electrode signals are simulated with pseudo‐ECG, lead field and similar approaches. In particular, this formulation models the 60–6 well MEA system from Multi Channel Systems (https://www.multichannelsystems.com)schematically depicted in Fig. 1, which consists of six independent wells, each containing nine electrodes, enabling parallel experiments under consistent conditions.

The system also embeds the single‐cell ionic model, through the transmembrane ionic current $I_{\mathrm{ion}}=I_{\mathrm{ion}}\;\left(v,w,c\right)$, which reproduces the activity of hiPSC‐CMs. This encompasses the function of various transmembrane channels along with the intracellular calcium (Ca^2+^) dynamics, which are characterized by the system of ODEs presented in Eq. (5d). The vector of state variables, denoted as w, includes the gating variables $w^{1},\text{…},w^{s}$ whereas c represents the vector of concentrations, specifically $c^{1},\;c^{2},\;c^{3}$. The system is coupled with appropriate initial conditions for the transmembrane potential, gating variables, and ionic concentrations

(6) $$v\;\left(x,0\right)=v_{0}\;,\;w\;\left(x,0\right)=w_{0}\;,\;c\;\left(x,0\right)=c_{0.}\;$$

We also impose the following boundary conditions, where **n** is the outward pointing normal:

- homogeneous Neumann boundary conditions associated with the no‐flux assumption of the intracellular potential on the external surface $\partial \Omega :D_{i}\nabla \left(v+u_{e}\right)\cdot n=0$ on ∂Ω×(0,T);
- homogeneous Dirichlet boundary conditions on the three edges connected to the ground: $u_{e}\cdot n=0$ on $\partial \Omega_{D}\times \left(0,T\right);$
- Neumann no‐flux boundary condition for the remaining edge: $D_{e}\nabla u_{e}\cdot n=0$ on $\partial \Omega_{N}\times \left(0,T\right)$.

### Tissue heterogeneity, VL and AL ionic models

Electrophysiological diversity and variability are notable characteristics of stem cell preparations. This heterogeneous behaviour mainly arises from the intrinsic variations in the cell lines employed in the experiments, as well as the specific conditions under which the cells undergo differentiation.

To model the electrophysiological heterogeneity seen in the experimental setup, we introduce both AL and VL phenotypes in the same domain. In accordance with Altomare et al. (2023), 30% of the cells recorded in a standard plate can be defined as AL CMs. Thus, when studying the conduction velocity, we randomly labelled 30% of points of the discretized grid as AL hiPSC‐CMs, as depicted in Fig. 2 (blue squares). The rest of the dish is considered to be made up by VL hiPSC‐CMs (red squared dots in Fig. 2). Furthermore, in MEA platforms, the electrical activity of hiPSC‐CMs is initialized by a small group of cells and propagated to the neighbouring CMs via gap junctions. Since the phenotype of hiPSC‐CMs in a platform is generally differentiated between AL and VL, with a higher percentage of VL cells in the untreated configuration (Doss et al., 2012), we assume that the propagating wave originates from a point source in the left‐bottom corner, made up of faster VL hiPSC‐CMs (dark squared dots in Fig. 2).

**Figure 1:**
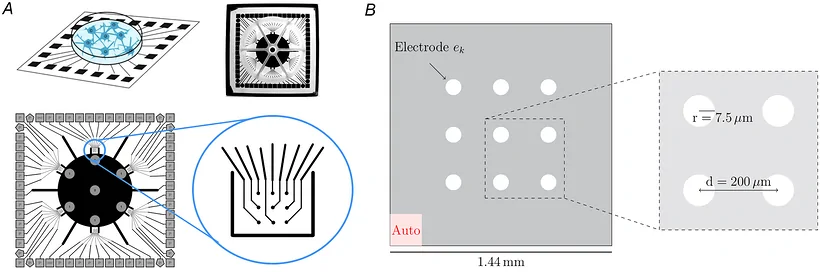
Schematic representation of the MEA device *A*, detailed representation of the experimental setup. The top row depicts two pictures of the device, showing the interaction with the culture of cells (on the left) and the position of the six independent culture chambers, separated by a macrolon ring (photo on the right). The bottom row depicts a schematic diagram of the MEA chip (on the left): inside each well, in between the marked two bars exiting from the circle in the middle of the MEA, there is a field of nine electrodes with an internal reference electrode (see the zoomed view on the right). *B*, geometry used in the MEA model simulations. The domain contains 3 × 3 evenly spaced electrodes, and the propagating wave is initiated by a group of spontaneously firing cells in the lower left corner of the domain.

**Figure 2:**
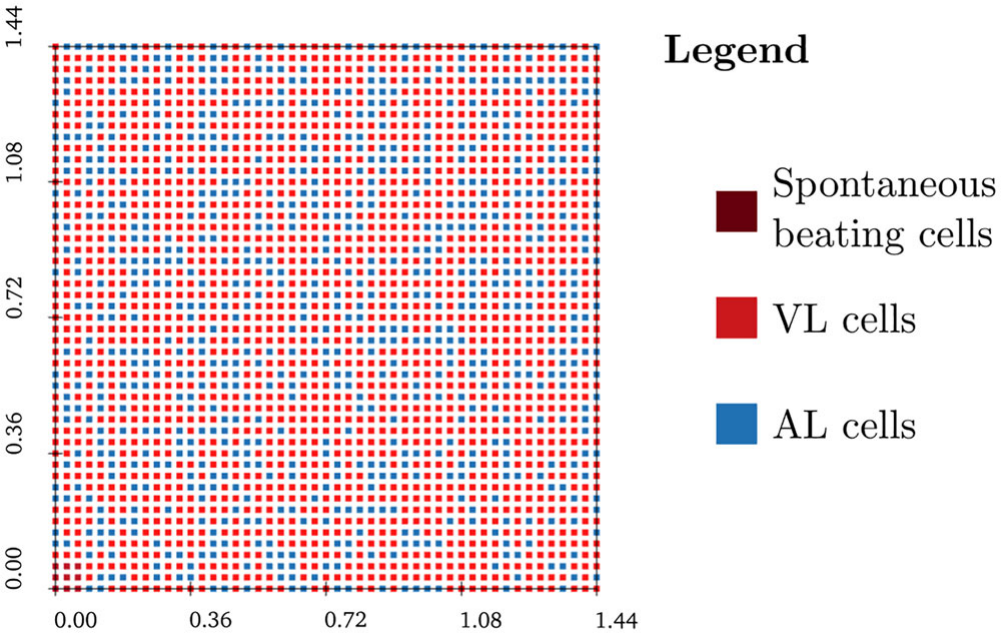
Heterogenous tissue configuration Random position of AL and VL cells inside the MEA well when considering 30% of AL hiPSC‐CMs. In the bottom left corner, a group of spontaneously beating VL cells is imposed in this configuration. If the percentage of AL hiPSC‐CMs is >50, then a group of spontaneously beating AL cells has to be imposed.

To model the single‐cell phenotypic variability, we employed the latest version of the Paci2020 hiPSC‐CM ionic model presented in Paci et al. (2020) to model the VL hiPSC‐CM phenotype. This model reflects both the automatic firing activity (dashed dark red line in Fig. 3) and the response to a given stimulus (solid red line in Fig. 3) In addition, we adopt the novel atrial‐specific ionic model Botti2024, presented in Botti et al. (2024), to simulate the AL hiPSC‐CM phenotype. This model accurately reflects the AL AP under external stimulation and injection of additional $I_{K1}$; nevertheless, the immature condition showing spontaneous firing activity arises for lower values of $I_{K1}$ conductance ($g_{K1}$). Thus, here we considered a modified version of the published model, setting $g_{K1\;}=0.05$ [nS*/*pF], which also reflects both the automatic firing activity (dashed dark blue line in Fig. 3) and the response to a given stimulus (solid blue line in Fig. 3).

**Figure 3:**
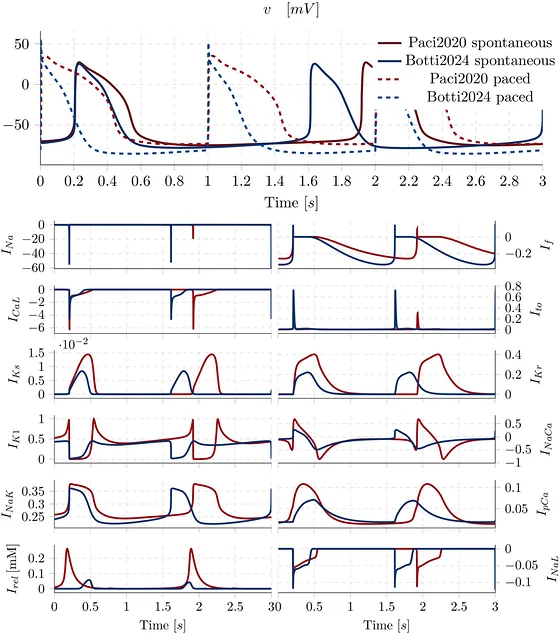
Phenotype‐specific ionic models Tissue heterogeneity is described using two different ionic models: the Paci2020 model for VL hiPSC‐CMs and the Botti2024 model for AL hiPSC‐CMs. They simulate both the spontaneous firing activity (used for the automatic corner, solid lines in the upper panel) and the response to a given stimulus (used in the rest of the domain, dashed lines in the upper panel). Ionic currents that underlie the differences between the AL and VL hiPSC‐CMs are shown in the other panels. Notably, the primary distinction is the presence of atrial‐specific $I_{\mathrm{Kur}}$ and $I_{\mathrm{KCa}}$ currents in the AL hiPSC‐CMs. For the sake of clarity, only currents in spontaneously firing hiPSC‐CMs are shown, allowing for better visualization of non‐overlapping traces. Unless otherwise specified, units of ionic current are expressed as [pA*/*pF].

The ionic term of both these ionic models is the sum of sodium (Na^+^) currents ($I_{\mathrm{Na}}$, $I_{\mathrm{Na}L}$, $I_{b\mathrm{Na}}$), potassium (K^+^) currents ($I_{\mathrm{to}},\;I_{\mathrm{Kr}},\;I_{\mathrm{Ks}},\;I_{K1}$), Ca^2+^ currents ($I_{\mathrm{Ca}L}$, $I_{b\mathrm{Ca}}$), as well as the funny current $I_{f}$, the Na^+^/Ca^2+^ exchanger $I_{\mathrm{NaCa}}$ and the Na^+^/K^+^ and the sarcolemmal Ca^2+^ pumps ($I_{\mathrm{NaK}},\;I_{p\mathrm{Ca}}$). The atrial Botti2024 model also takes into account the Ca^2+^ currents $I_{\mathrm{Kur}}$ and $I_{\mathrm{KCa}}$.

In the mathematical description of the model, tissue heterogeneity is introduced in the PE MEA formulation by solving different ionic models in Eq. (5d) when considering different nodes and the source term in Eq. (5a) accordingly. For this purpose, we introduce $\Omega_{1}\;:=\;\cup_{i\in I_{1}}\omega_{i}$ where $I_{1}$ is the set of nodes labelled AL, $\Omega_{2}\;:=\;\cup_{i\in I_{2}}\omega_{i}$, where $I_{2}$ is the set of nodes labelled VL, and $\Omega_{3}\;:=\;\cup_{i\in I_{3}}\omega_{i}$, where $I_{3}$ is the set of pacemaker nodes, being $\Omega =\Omega_{1}\cup \Omega_{2}\cup \Omega_{3}$. Thus, the system (5) is modified accordingly and Eq. (5d) becomes


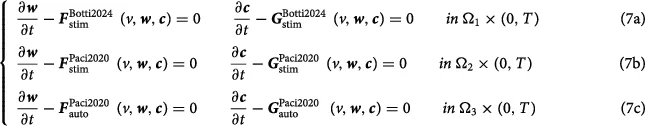




The source term in Eq. (5a) and initial conditions in Eq. (6) are modified accordingly.

We note that, when considering percentages of AL hiPSC‐CMs >50%, the ionic model used in $\Omega_{3}$ is the automatic version of the AL Botti2024 model.

### Tissue conductivity

In adult cardiac tissue, the arrangement of fibres rotating anticlockwise from epi‐ to endocardium facilitates the propagation of electrical signals in a preferred direction. The conductivity of hiPSC‐CM tissue also depends on the spatial arrangement and geometric orientation of the cells, as well as the expression of gap junctions and other biological factors associated with cell maturation (MacQueen et al., 2018; Botti & Torre, 2023). Coherently, the general two‐dimensional anisotropic conductivity tensors $D_{i}\left(x\right)$ and $D_{e}\left(x\right)$ are defined as:

(8) $$\begin{array}{ccc} D_{i,e}\;\left(x\right) & = & \sigma_{l}^{i,e}\;a_{l}\left(x\right)a_{l}^{⊤}\left(x\right)+\sigma_{t}^{i,e}a_{t}\left(x\right)a_{t}^{⊤}\;\left(x\right)\\ & & \;\;\;=\;\sigma_{t}^{i,e}\;I+\left(\sigma_{l}^{i,e}-\sigma_{t}^{i,e}\right)a_{l}\left(x\right)a_{l}^{⊤}\left(x\right) \end{array}$$

where $a_{l}\left(x\right),\;a_{t}\left(x\right)$ is a pair of orthonormal principal axes in R, and $\sigma_{l}^{i,e}$, $\sigma_{t}^{i,e}$ are the conductivity coefficients in the intra‐ and extracellular media measured along the corresponding directions $a_{l},\;a_{t}$.

Since cell maturation affects conductivity in an unknown manner, resulting in a complex experimental quantification of the parameters, and since no preferential conduction direction is shown in MEA recordings, we consider an axisymmetric isotropic mediUM with $\sigma_{l}^{i,e}=\sigma_{t}^{i,e}$, and we define the resulting conductivity tensors as

(9) $$D_{i,e}\;\left(x\right)=\sigma^{i,e}\;I$$

We choose $\sigma^{i}=0.01$ mS*/*cm and $\sigma^{e}=0.1$ mS*/*cm as suggested in Jæger et al. (2021a) and which results in a conduction velocity equal to about 4 cm*/*s, matching the values in Abbate et al. (2018). These values are lower than those observed in human cardiac tissue, as the conduction in hiPSC‐CMs is significantly slower compared to that in the human heart.

### Numerical methods

The MEA system of partial differential equations (PDEs) is discretized in space with piecewise linear finite elements, using a uniform tetrahedral triangulation $T^{h}$ of Ω, i.e. a 2D hexahedral mesh with h=30μm (Hughes, 2012; see Colli Franzone et al. 2014, Ch. 7). The finite element interpolant of *U* is numerically computed at each time step as the trapezoidal numerical integral of the scattered interpolant of $u_{e}$ on the $k^{\mathrm{th}}$ electrode.

Furthermore, the system of PDEs is discretized in time using a decoupled semi‐implicit backward Euler scheme, to ensure an accurate integration of the PE MEA system's temporal evolution (Sundnes et al., 2007). To ensure a small relative error on the AP, we adapted the temporal time‐step, setting Δt=1.0μs.

In the different setups presented in the Results, the simulations for updating the initial conditions of the ionic models (see Fig. 3) were performed by solving systems of ODEs using the Matlab built‐in solver ode15s, which is designed for stiff systems (Shampine & Reichelt, 1997). The solver was configured with an initial step size of $2\times 10^{-5}$ s and a maximum allowable step size of 1 ms. Numerical tests were conducted by varying the relative and absolute tolerance parameters of ode15s, with values set to $\mathrm{RelTol}\;=10^{-3}$ and $\mathrm{AbsTol}\;=10^{-6}$ to ensure accurate reproduction of the AP morphology.

Our implementation utilizes parallel computing on a single CPU with 32 threads, achieving a runtime of ∼3 h for each simulation involving the full computational domain. This efficiency is largely due to the implicit time‐stepping scheme and domain decomposition strategies used in the code.

### Activation time, repolarization time and AP duration

We define the activation time AT(x) at point x as the earliest time instant when $v\;\left(x,\;AT(x)\right)=v_{\mathrm{up}}$, where $v_{\mathrm{up}}=-50$ mV, a value exceeding the threshold needed to trigger the upstroke of the AP.

We define the repolarization time RT(x) at point x as the first time instant after the upstroke when the membrane potential reaches $v_{\mathrm{down}}=-45$ mV, i.e. $v\;\left(x,\;RT(x)\right)=v_{\mathrm{down}}$.

The AP duration (APD) at each point ***x*** is then computed as the difference between repolarization and activation times: APD(x)=RT(x)−AT(x).

### Conduction velocity

We compute locally the conduction velocity using a finite difference framework as in Cantwell et al. (2015). In this paper, derivatives are approximated at a given grid‐point, through differences between neighbouring grid‐points, using a stencil. This approach can be used to compute at each point in the grid the local conduction velocity estimates.

The horizontal and vertical components of the gradient of activation **G**, in the directions **i** and **j** respectively, are computed using standard first‐order finite‐difference stencils as

$$\begin{array}{c} x_{i,j+1}\\ |\\ x_{i-1,j}\text{--}x_{i,j}\text{--}x_{i+1,j}\\ |\\ x_{i,j-1} \end{array}\;\begin{array}{c} G_{x}\;\left(x_{i,j}\right)=\frac{AT\left(x_{i+1,j}\right)-AT\left(x_{i-1,j}\right)}{2h}\;\;i,\\ G_{y}\;\left(x_{i,j}\right)=\frac{AT\left(x_{i,j+1}\right)-AT\left(x_{i,j-1}\right)}{2h}\;\;j \end{array}$$

where *h* is the distance between two adjacent nodes of the mesh triangulation of the domain Ω. The conduction speed |**u**| and the unit vector $\hat{n}$ indicating the direction of activation are then expressed as

$$\begin{array}{ccc} \;\left|u\right| & = & \frac{1}{\left|G_{A}\right|}=\frac{1}{\sqrt{G_{x}^{2}+G_{y}^{2}}}\;,\\ \hat{n} & = & i\frac{G_{x}}{\sqrt{G_{x}^{2}+G_{y}^{2}}}+j\frac{G_{y}}{\sqrt{G_{x}^{2}+G_{y}^{2}}} \end{array}$$

leading to the conduction velocity vector:

(10) $$u=\left|u\right|\;\hat{n}=i\frac{G_{x}}{G_{x}^{2}+G_{y}^{2}}+j\frac{G_{y}}{G_{x}^{2}+G_{y}^{2}}$$

We also consider the average conduction velocity across our MEA domain, defined as in Jæger et al. (2021c), as the distance between the centre of the two points on the diagonal (A and B) divided by the time between the activation time crosses the two points, i.e.

(11) $$\mathrm{cv}\;=\frac{\left|x_{A}-x_{B}\right|}{\left|AT\left(x_{A}\right)-AT\left(x_{B}\right)\right|}\;$$

in cm/s, where A and B are 0.2121 mm distant from the bottom left corner and the upper right one, respectively.

### Brugada syndrome modelling

Brugada syndrome (BrS) is strongly linked to mutations in genes encoding ion channel proteins. In this study, we model two BrS cases associated with two different mutations.

The first one (referred to as BrS1) is associated with mutations in the SCN5A gene, responsible for the α‐subunit of the cardiac Na^+^ channel. SCN5A mutations are present in ∼20% of clinically diagnosed BrS1 cases and are known to impair Na^+^ channel function. This leads to a reduction in the Na^+^ current, contributing to an imbalance of ionic currents during the early phases of the AP. Such disturbances predispose the myocardium, particularly the right ventricular outflow tract, to arrhythmic events by creating a heterogeneous electrical substrate. Na^+^ channel blockers further exacerbate this electrophysiological profile, reinforcing the diagnostic and pathophysiological significance of SCN5A mutations (Tse et al., 2016).

The second BrS case we consider (referred to as BrS2) is linked to L‐type Ca^2+^ channel (LTCC) mutations. LTCC comprises subunits encoded by genes such as CACNA1C, CACNB2 and CACNA2D1. Loss‐of‐function mutations in these genes impair LTCC expression, trafficking or function, leading to reduced Ca^2+^ current (Antzelevitch et al., 2007). Although less prevalent than SCN5A mutations, LTCC‐related BrS presents a unique pathophysiological profile that warrants further investigation. These mutations highlight the importance of Ca^2+^ dynamics in maintaining electrical stability and preventing arrhythmias (Tse et al., 2016).

*BrS1*. Following the approach proposed by Hoogendijk et al. (2011) and Scacchi et al. (2023), where ST‐segment elevation in BrS1 is linked to reduced Na^+^ channel ($g_{\mathrm{Na}}$) and L‐type Ca^2+^ channel ($g_{\mathrm{CaL}}$) conductances alongside increased K^+^ channel ($g_{\mathrm{to}}$) conductance, we simulate these ionic alterations using the Paci2020 VL hiPSC‐CM model. In our approach, we adjust $g_{\mathrm{Na}}$, $g_{\mathrm{CaL}}$ and $g_{\mathrm{to}}$ under the assumption that all cells within the simulated tissue region exhibit characteristics of BrS1. Furthermore, since our interest is focused on a ventricular‐specific disorder, we consider the standard differentiation protocol (Altomare et al., 2023), which leads to 30% of VL hiPSC‐CMs in the culture. Specifically, we consider the following setting in the Paci2020 model, which leads to the single cell response depicted in Fig. 9 below:

- $g_{\mathrm{Na}}$ is reduced and set at 70% of its normal value;
- $g_{\mathrm{CaL}}$ is reduced and set at 90% of its original value;
- $g_{\mathrm{to}}$ is increased and set to 110% of its normal value.

*BrS2*. Loss‐of‐function mutations involving LTCC are known to predispose to a phenotype consisting of BrS2 with an abbreviated QT. Through a bottom‐up approach, we base our BrS2 model on Burashnikov et al. (2010), which considered a p.V2014I‐CACNA1C mutation causing a loss of function of Ca^2+^, and involving the co‐presence of a p.D601E polymorphism in CACNB2b that augments $I_{\mathrm{CaL}}$. To model this specific mutation, we performed the same shift voltage dependencies of activation and inactivation variables as depicted in fig. 5 of Burashnikov et al. (2010). In the Paci2020 model, the analytical expression of $I_{\mathrm{CaL}}^{*}$, accordingly modified to replicate the changes between the control (Ctrl) and the p.V2014I‐CACNA1C mutant channel (BrS2), is

$$\begin{array}{ccc} \;I_{\mathrm{CaL}}^{*} & = & \frac{4\cdot v\cdot F^{2}\cdot g_{\mathrm{CaL}}}{R\cdot T}\;\cdot \frac{\left(Ca_{i}\cdot \exp\left(\frac{2\cdot v\cdot F}{R\cdot T}\right)-0.341\cdot Ca_{o}\right)}{\exp\left(\frac{2\cdot v\cdot F}{R\cdot T}\right)-1}\\ & & \cdot \;d^{*}\cdot f1^{*}\cdot f2\cdot \mathrm{fCa} \end{array}$$

where $g_{\mathrm{CaL}}$ is the maximal conductance, $Ca_{i}$ and $Ca_{o}$ the subspace and extracellular Ca^2+^ concentrations, respectively, $d^{*}$ the activation gate, $f1^{*}$ f2 the voltage‐dependent inactivation gates, fCa the Ca^2+^‐dependent inactivation gate, v and VmV the transmembrane potential in volts and millivolts, respectively, F the Faraday constant, R the gas constant and T the absolute temperature. All parameter values are those reported in Botti et al. (2024).

The activation function $d_{\infty }^{*}$ is

$$\;d_{\infty }^{*}=\frac{1}{1+\exp\left(\frac{-VmV-v_{a0.5}^{*}}{7}\right)}$$

with activation voltage for 50% channels open $v_{a05}^{*}$ given by $v_{a0.5}^{\mathrm{Ctrl}}=8.1$ mV, $v_{a0.5}^{\mathrm{BrS}2}=8.1$ mV.

The inactivation function $f1_{\infty }^{*}$ is

$$\;f1_{\infty }^{*}=\frac{1}{1+\exp\left(\frac{VmV+v_{\mathrm{ina}0.5}^{*}}{3}\right)}$$

with inactivation voltage for 50% channels open $v_{\mathrm{ina}0.5}^{*}$ given by $v_{\mathrm{ina}0.5}^{\mathrm{Ctrl}}=26$ mV, $v_{\mathrm{ina}0.5}^{\mathrm{BrS}2}=40$ mV.

The parameters were adjusted so that our activation ($d_{\infty }^{*}$) and inactivation ($f1_{\infty }^{*}$) curves, displayed in Fig. 9, match at least qualitatively the experimental plots reported in fig. 5E and 5F of Burashnikov et al. (2010), and the resulting effects in our simulated pathological $I_{\mathrm{CaL}}^{*}$ waveforms, also displayed in Fig. 9, are comparable with those observed in fig. 5 of Burashnikov et al. (2010).

### Drug modelling

In the final part of the study, *in silico* drug trials were performed for four compounds and were compared with experimental observations.

To this end, we need a mathematical model of the effect of each drug on the properties of a mutant ion channel. We base this on the general formulation proposed in Jæger et al. (2021b) for both ion channel blockers (antagonists) and openers (agonists), which reflects drugs alter channel dynamics and conductance is limited. To do this, we use the assumption that the effect of a drug on a current I can be written in the form

(12) $$I\;\left(\left[D\right]\right)=\left(1+\frac{{\left(\varepsilon \left[D\right]\right)}^{H}}{{\left(\varepsilon \left[D\right]\right)}^{H}+1}E\right)\;I,$$

where E denotes the maximum effect of the drug, [D] is the concentration of the drug, *H* stands for the Hill coefficient and $EC_{50}=1/\varepsilon$ is the concentration that corresponds to the half maximum effect of the drug. To use this model, we need to estimate ε, *H* and *E* from data that describe the drug's effects on the ion current properties of the myocyte with the mutation. When the drug acts as a blocker, it is often practical to set E=−1, which allows Eq. (12) to assume the familiar form of the $IC_{50}$ model, as also noted in Paci et al. (2015). In this context, drug block is modelled using a straightforward pore block approach that influences the relevant maximum conductances according to a dose–response curve for each ionic current, *I*, as outlined below:

(13) $$I\;\left[D\right]=\frac{1}{1+{\left(\frac{\left[D\right]}{IC_{50}}\right)}^{H}}\;I$$

where $IC_{50}=1/\varepsilon$ is the drug concentration required for 50% inhibition. Both values are considered in µM in the formula. Note that when estimating *E*, there is a clear lower bound of E=−1, but no evident upper limit exists.

To better quantify the current reduction or increase, we define an integral biomarker in [(pA·ms)/pF], i.e. the integral of the current *I* over time, defined as

(14) $$\mathrm{Int}\;\left(I\right)=\int_{0}^{600}\left|I\left(t\right)\right|dt\;.$$

## Results

### Activation time maps

The excitation wavefronts are visualized by plotting the isochrone lines of the activation times across the MEA tissue. Figure 4 illustrates the activation times for six different configurations: two representing homogeneous configurations (one composed entirely of VL cells and another entirely of AL cells) and four representing heterogeneous mixtures, where AL cells constitute 20, 40, 60 and 80% of the ventricular substrate, where they are randomly distributed. A qualitative assessment suggests that AL tissues exhibit faster overall activation, requiring less time to achieve complete excitation compared to the other configurations. This observation underlines the influence of cellular composition on the electrical behaviour of the tissue, particularly in terms of activation dynamics.

**Figure 4:**
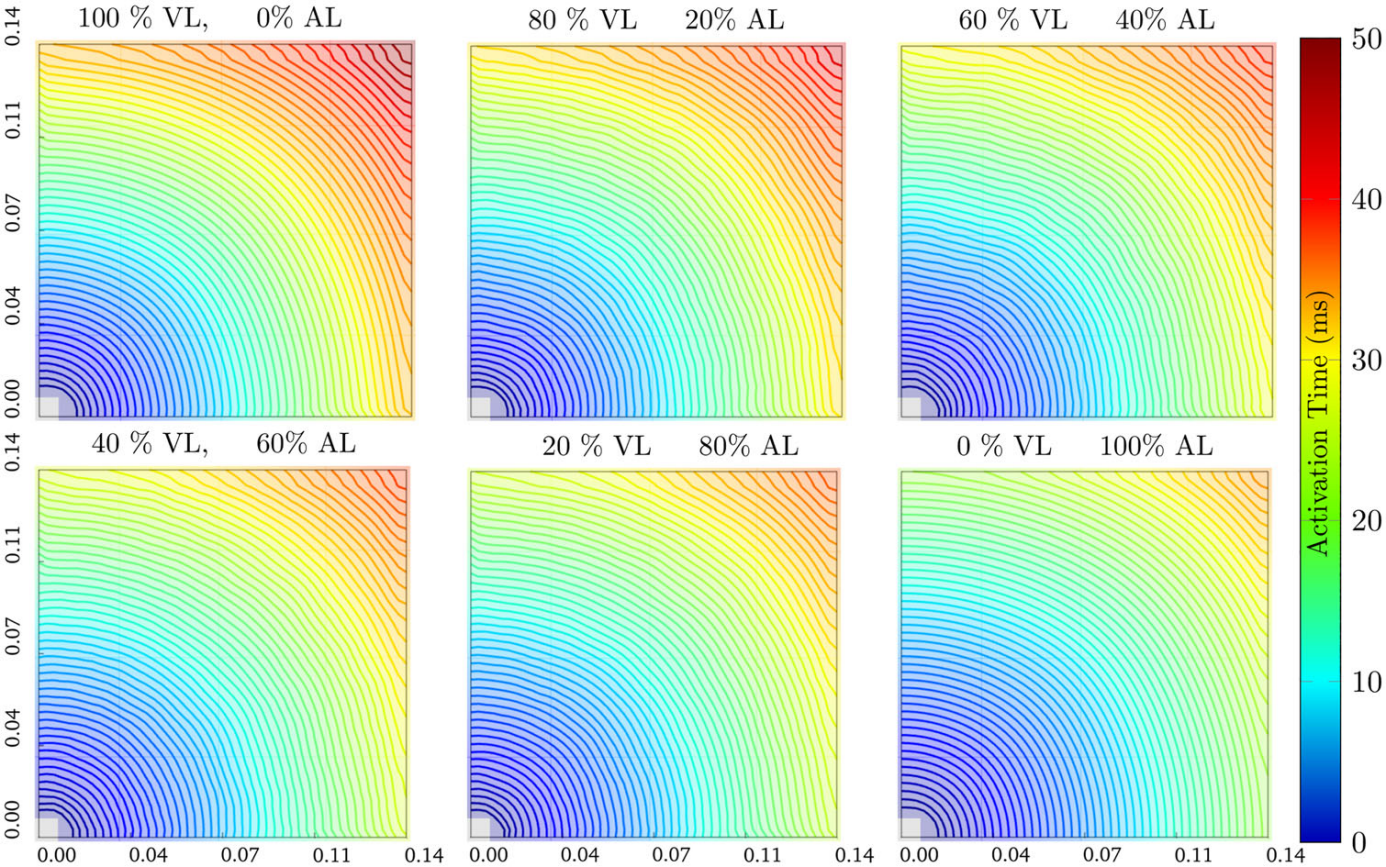
Activation time maps Activation time isochrones computed by solving the MEA model when considering homogeneous VL or AL tissue, or mixed configurations of phenotype‐specific hiPSC‐CMs in the well. The colour bar indicates the range of values represented by the displayed equipotential lines.

### Local and average conduction velocity

In this study, we examine both the local conduction velocity, which is calculated point‐by‐point, and the average conduction velocity of the activation wavefront. Both velocity metrics are computed from the local activation times.

Figure 5 shows the pointwise conduction speed |**u**| together with the conduction velocity vectors, computed as detailed in the Methods, for MEA tissues with different mixes of VL and AL cells, ranging from tissues with 100% VL cells (top left) to tissues with 100% AL cells (bottom right). These plots show that tissues with AL cells exhibit a higher conduction velocity compared to tissues with VL hiPSC‐CMs.

**Figure 5:**
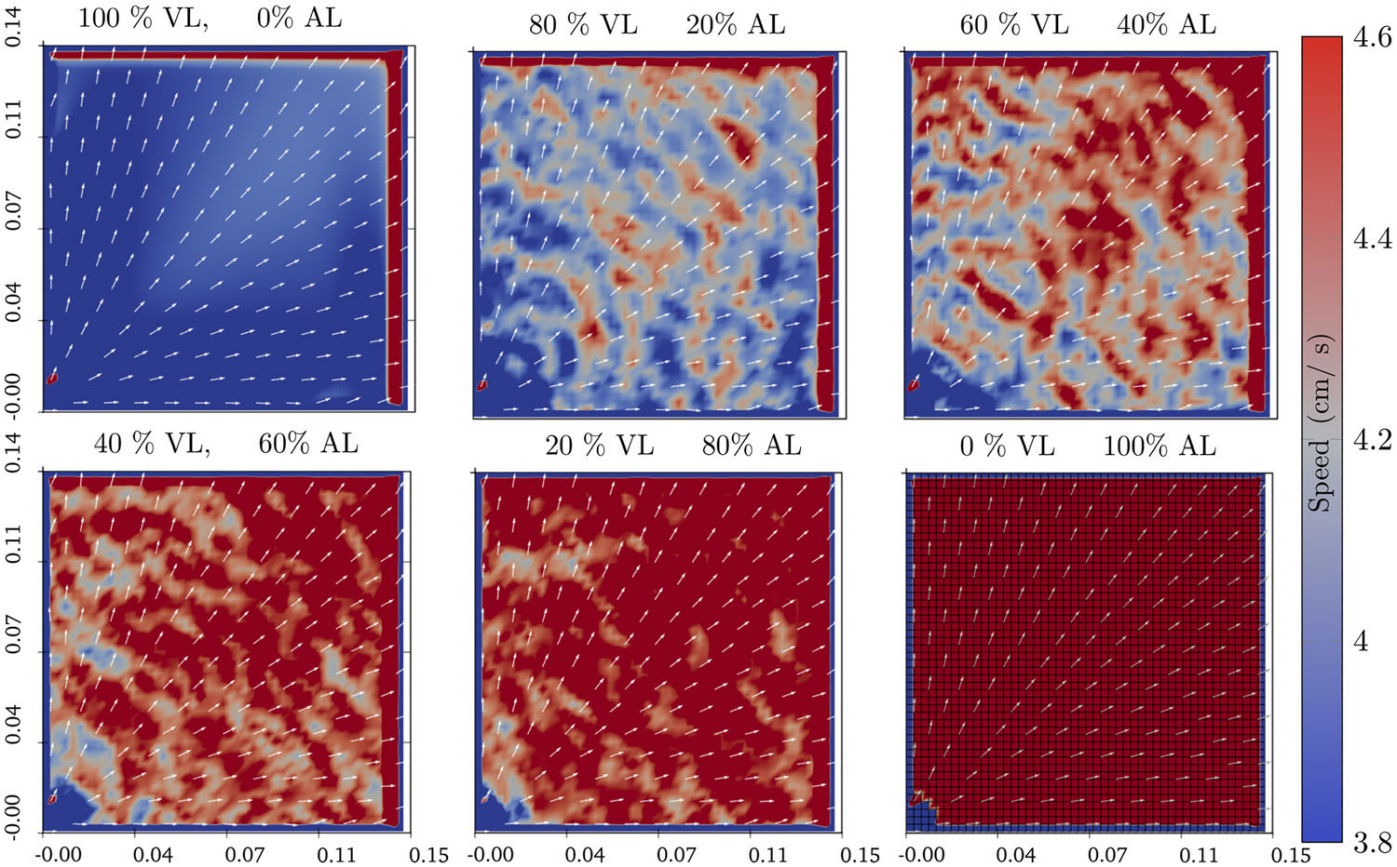
Local conduction speed maps The local conduction speed vector field was computed at each point, considering either homogeneous VL or AL tissue, as well as a mixed configuration of phenotype‐specific hiPSC‐CMs within the well. To enhance clarity, 20% of the vector field arrows are displayed.

Figure 5 also shows the spatial variation of the conduction velocity within heterogeneous tissues. In contrast to the relatively uniform activation time shown in Fig. 4, the conduction velocity in heterogeneous tissues is highly sensitive to local cellular phenotypes. The presence of different phenotypic regions within the tissue results in a non‐uniform distribution of conduction velocity, creating areas of faster or slower propagation depending on the local composition.

Moreover, the analysis intentionally excludes the effects near the tissue boundaries visible in Fig. 5, which are often subject to distortions or edge effects that could skew interpretation of the conduction properties. Instead, the focus is placed on the interior region of the tissue domain, where conduction velocity is assumed to be more representative of the intrinsic properties of the tissue. By isolating the central area of the tissue, the study aims to provide a clearer understanding of how conduction velocity behaves in the core regions, free from potential artefacts introduced by edge conditions.

We also report in Fig. 8 below the average conduction velocity defined in Eq. (11) across the domain, which allows us to assert the strong dependency of average conduction velocity on the cellular phenotype. Specifically, velocity increases as the percentage of AL hiPSC‐CMs in the tissue rises.

### Repolarization time maps and APDs maps

Figure 6 shows the isochrone maps of the repolarization time for different heterogeneous tissues with increasing percentages of uniformly distributed AL cells. This quantitative analysis highlights the pronounced effects of heterogeneity, which are less noticeable on the activation map due to the rapid nature of the depolarization phase. However, these effects become more apparent during the slower process of tissue repolarization. As the degree of heterogeneity increases, the isochrones become less smooth, indicating more irregular and chaotic propagation patterns, with some regions of the tissue displaying closed isochrones. This suggests the presence of localized areas of abnormal repolarization.

**Figure 6:**
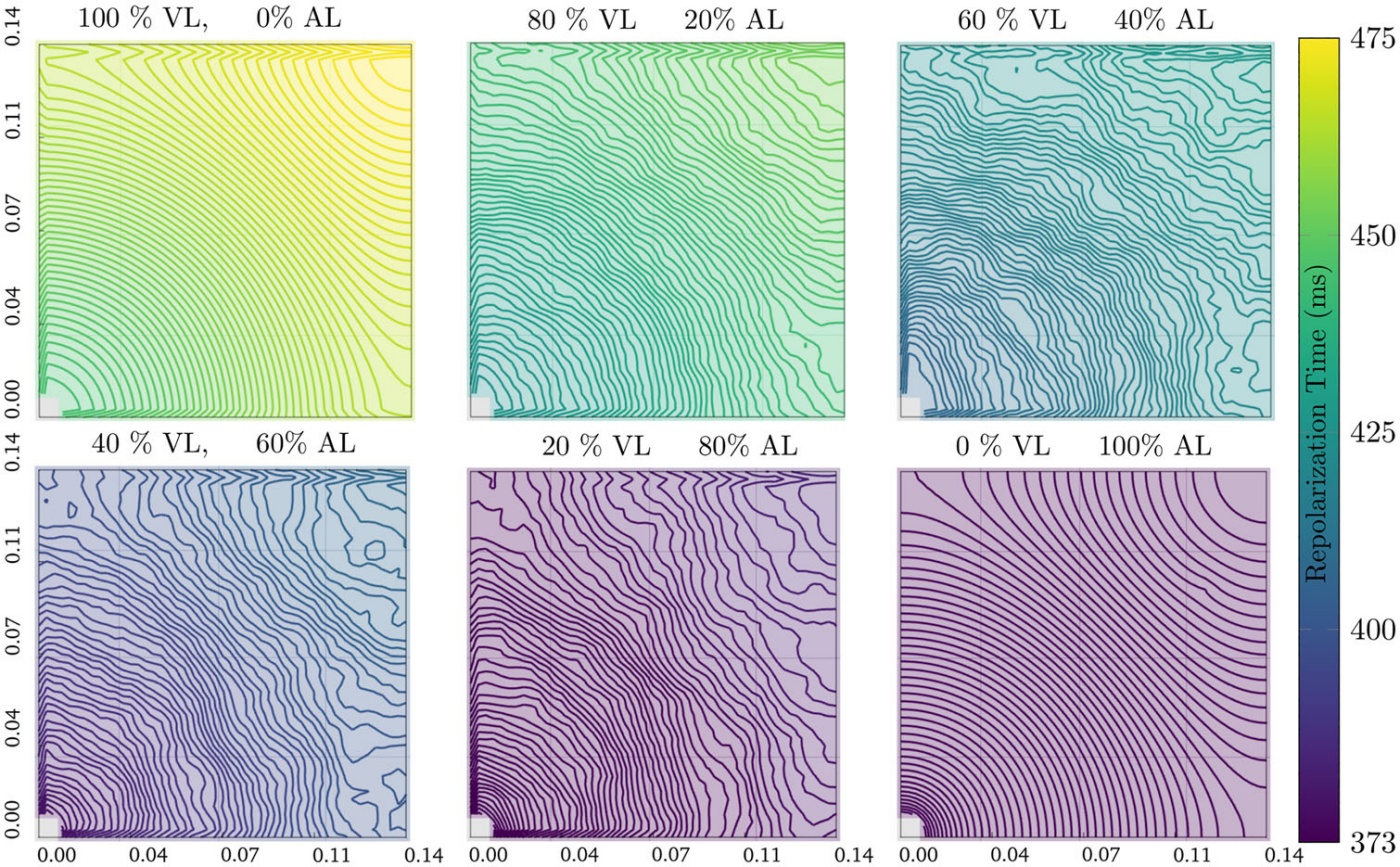
Repolarization time maps Isochrones of repolarization time were computed using the MEA model for various heterogeneous configurations of phenotype‐specific hiPSC‐CMs within the well. The colour scale indicates the range of repolarization times along the equipotential lines. Isochrones illustrate the irregular patterns in highly heterogeneous tissues, while the colour gradient emphasizes the prolonged recovery times for AL tissues returning to the resting state.

As expected due to the shorter AP duration in AL cells, the analysis of repolarization times shows that AL tissues repolarize earlier, requiring about 100 ms less than homogeneous VL tissue to return to the resting potential. This behaviour reflects the triangular shape of the AL AP, highlighting their faster recovery dynamics compared to other tissue types.

In addition to the analysis of repolarization times, we also examine the associated APD maps, which provide insights into the duration of the electrical activity of the tissue independent of activation time. This approach effectively normalizes the repolarization dynamics by focusing on the interval between depolarization and repolarization for each spatial point, eliminating any biases introduced by differences in activation time.

The APD maps in Fig. 7 reveal that despite normalization to the same initial time, late repolarization remains evident in VL‐dominant domains. This behaviour is characterized by prolonged APD values in these regions, consistent with the slower recovery dynamics of VL cells. On the other hand, AL‐dominant tissues exhibit significantly shorter APDs, reflecting their characteristic faster repolarization dynamics and triangular AP morphology.

**Figure 7:**
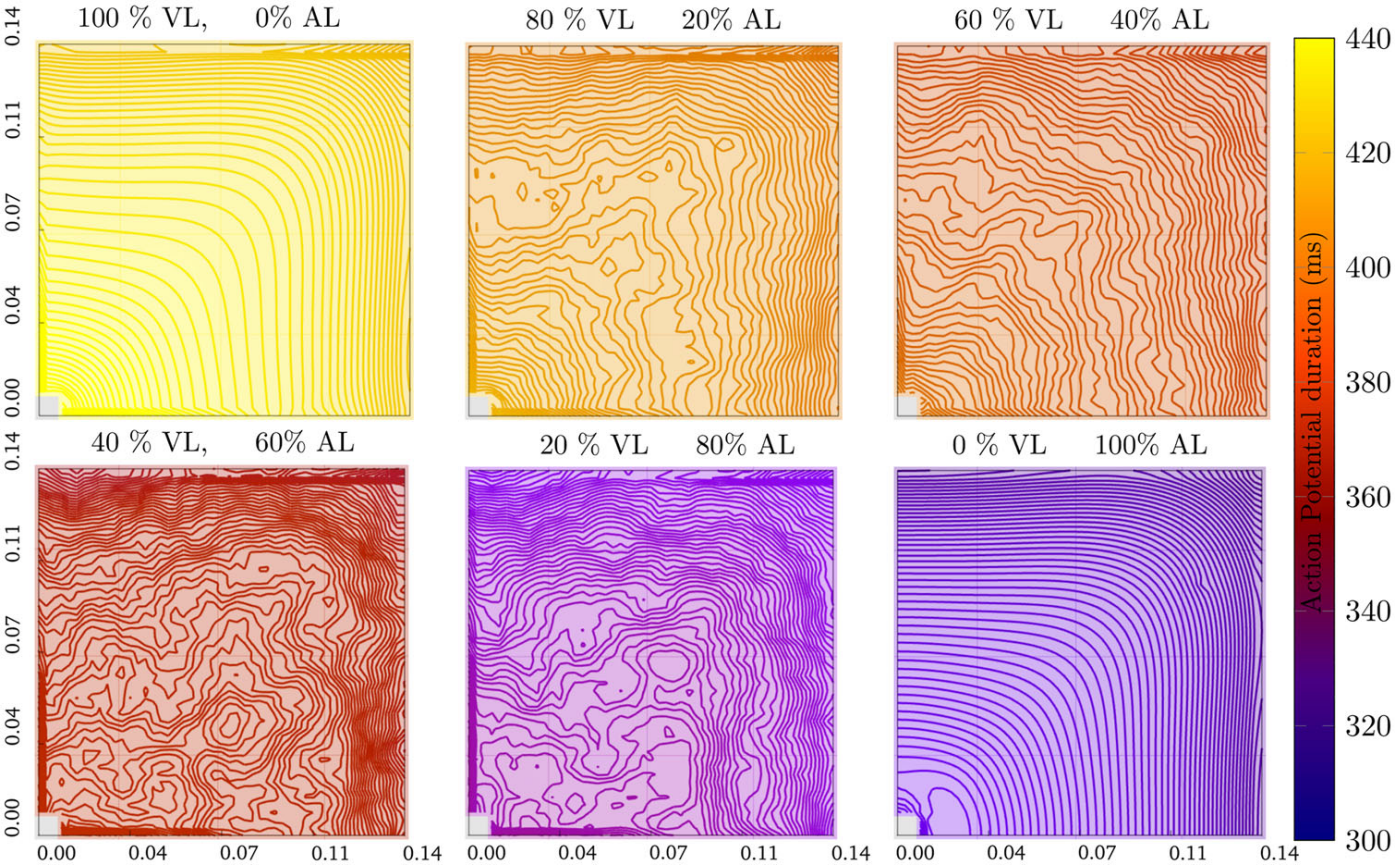
APD time maps Isochrones of APD were computed using the MEA model for various heterogeneous configurations of phenotype‐specific hiPSC‐CMs within the well. The colour scale indicates the range of APDs along the equipotential lines. Isochrones illustrate the irregular patterns in highly heterogeneous tissues.

**Figure 8:**
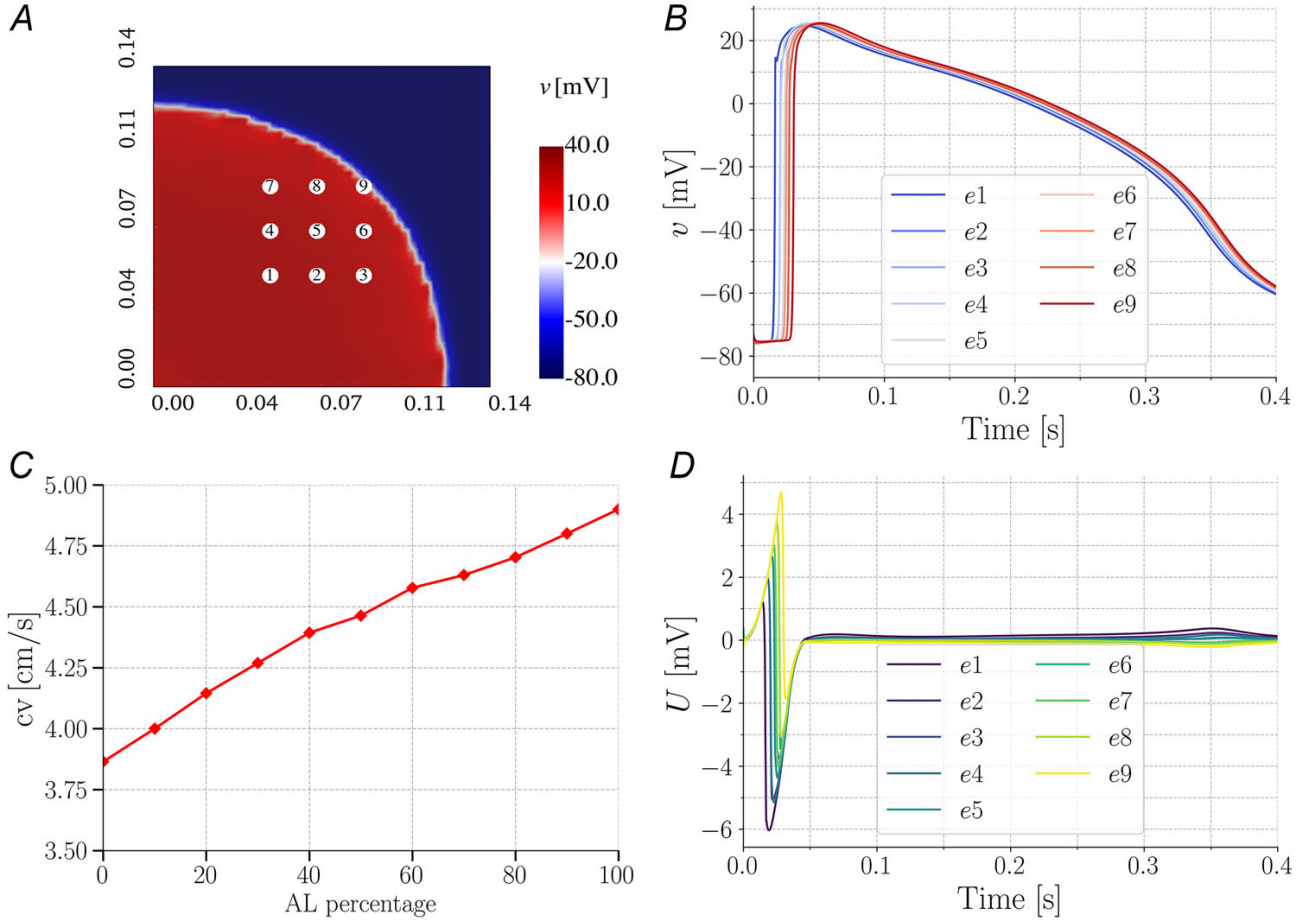
Heterogeneity effect on the average conduction velocity; AP and FP in the nine electrodes *A*, MEA domain with nine electrodes and transmembrane potential *v* about 30 ms after stimulation. *B* and *D*, AP and the FP waveforms at the nine electrodes placed as in *A*; Simulations WEre performed considering 30% AL hiPSC‐CMs in the MEA platform. *C*, increasing average conduction velocity for increasing percentage of AL hiPSC‐CMs in the MEA well. This relationship highlights how cellular composition directly influences electrical coupling efficiency and conduction properties.

Furthermore, as the degree of heterogeneity increases, the APD isochrones become less smooth, indicating more irregular and chaotic recovery patterns.

### Brugada syndrome results

In this section, we present the results of modelling two BrS cases, BrS1 associated with SCN5A mutations and BrS linked to LTCC mutations, through a bottom‐up approach (Burashnikov et al., 2010).

Figure 9 illustrates the simulated APs of a healthy VL hiPSC‐CM compared to two distinct BrS models. The solid black line represents the control condition, while the red and green dashed lines correspond to the BrS1 and BrS2 models, respectively. The figure demonstrates that BrS1 primarily affects $I_{\mathrm{Na}}$, $I_{\mathrm{CaL}}$ and $I_{\mathrm{to}}$, leading to alterations in depolarization dynamics. In contrast, BrS2 impacts $I_{\mathrm{CaL}}$, influencing both activation and inactivation processes. The bottom panels depict the gating function modifications implemented in the models, which subsequently alter the ionic currents via a bottom‐up approach. These ionic perturbations result in distinct AP morphologies (upper panel), with BrS1 exhibiting reduced upstroke velocity due to diminished $I_{\mathrm{Na}}$, while BrS2 shows changes in repolarization linked to altered $I_{\mathrm{CaL}}$ dynamics. These findings highlight the electrophysiological consequences of BrS subtypes at the single‐cell level, emphasizing the role of specific ionic mechanisms in shaping disease phenotypes.

**Figure 9:**
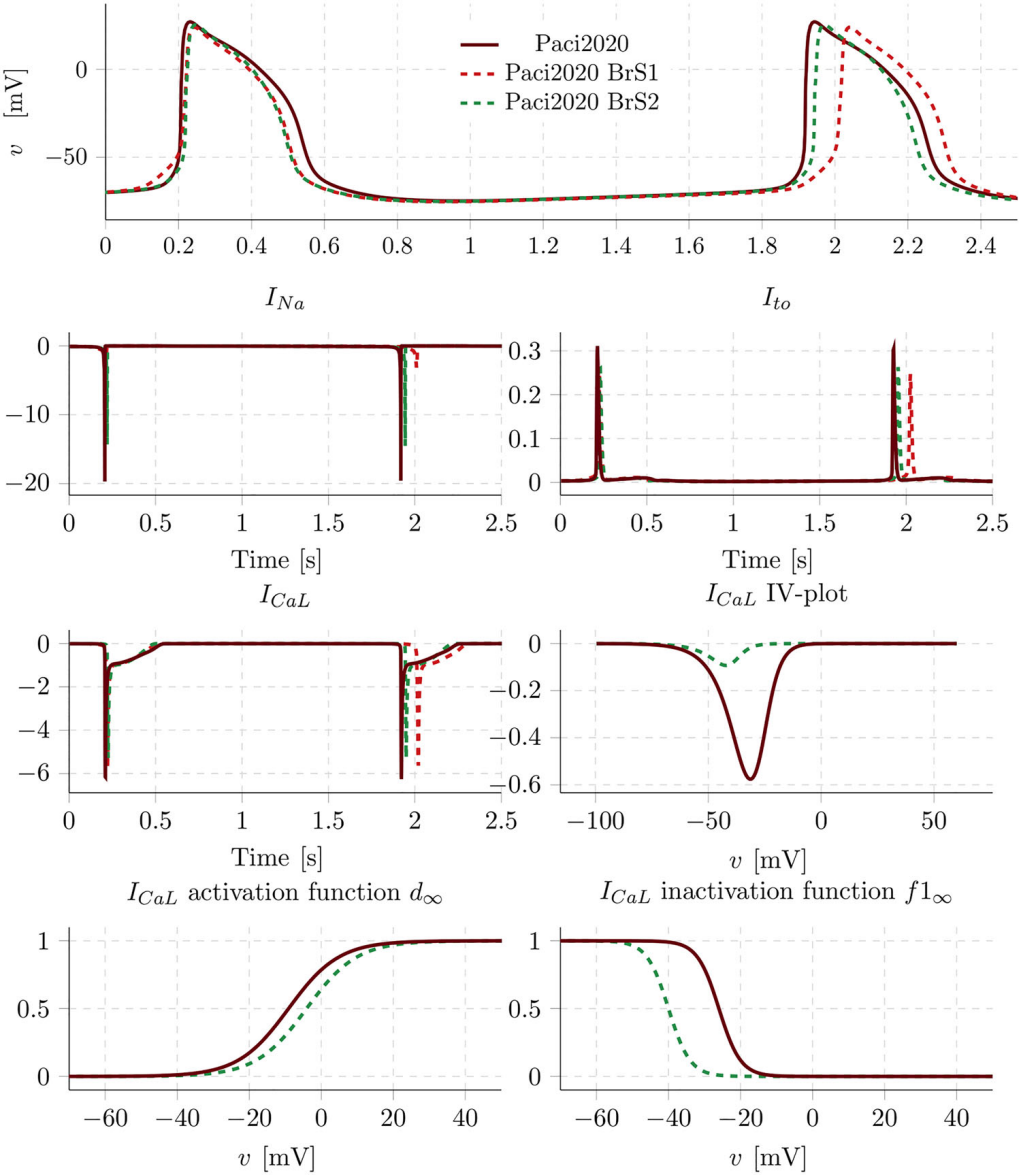
Ionic model comparison for healthy and BrS1‐ or BrS2‐affected VL hiPSC‐CMs Simulated AP of a healthy VL hiPSC‐CM (solid black line) alongside a modified model that incorporates changes to replicate the single‐cell effects characteristic of diseased hiPSC‐CMs (dashed lines). Red dashed lines refer to BrS1 modelling, while Green dashed lines refer to BrS2 modelling. The modifications of activation and inactivation curves (bottom panels) lead to modified ionic currents (bottom‐up approach). Thus, ionic currents reflect altered ionic dynamics associated with the diseased state, highlighting differences in AP morphology (upper panel).

Figure 10 shows the control, BrS1 and BrS2 waveforms of both the transmembrane potential (left) and FP (right) at a selected MEA electrode. The most prominent effect of both BrS1 and BrS2 simulations is the pronounced ST‐segment elevation and the markedly positive T‐wave, which result from rapid repolarization. These observations are consistent with the findings of both Scacchi et al. (2023), who simulated adult cardiac tissue affected by BrS1, and Burashnikov et al. (2010).

**Figure 10:**
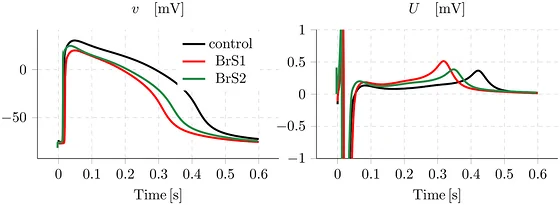
Electrical activity in the selected electrode AP *v* (left panel) and FP *U* (right panel), recorded by one electrode when considering hiPSC‐CM tissue in control conditions (black line) and when considering both the BrS1 and BrS2 changes affecting the VL ionic model (red and green lines, respectively). Note that the AP in the left panel is not equivalent to the single‐cell dynamics shown in Fig. 9.

Furthermore, at the tissue level, Fig. 11 indicates a reduction in conduction velocity, which is only taken into account for BrS1 incorporating SCN5A mutations. This observation is quantitatively confirmed by calculating the average conduction velocity using Eq. (11), which yields a value of 3.59 cm*/*s in the tissue affected by BrS1. In comparison, the conduction velocity in healthy tissue is measured at 4.27 cm*/*s. This marked decrease in conduction velocity in BrS1 tissue can be attributed to the impaired Na^+^ channel function and subsequent slower propagation of electrical impulses across the myocardium, a hallmark of the arrhythmogenic substrate seen in BrS1.

**Figure 11:**
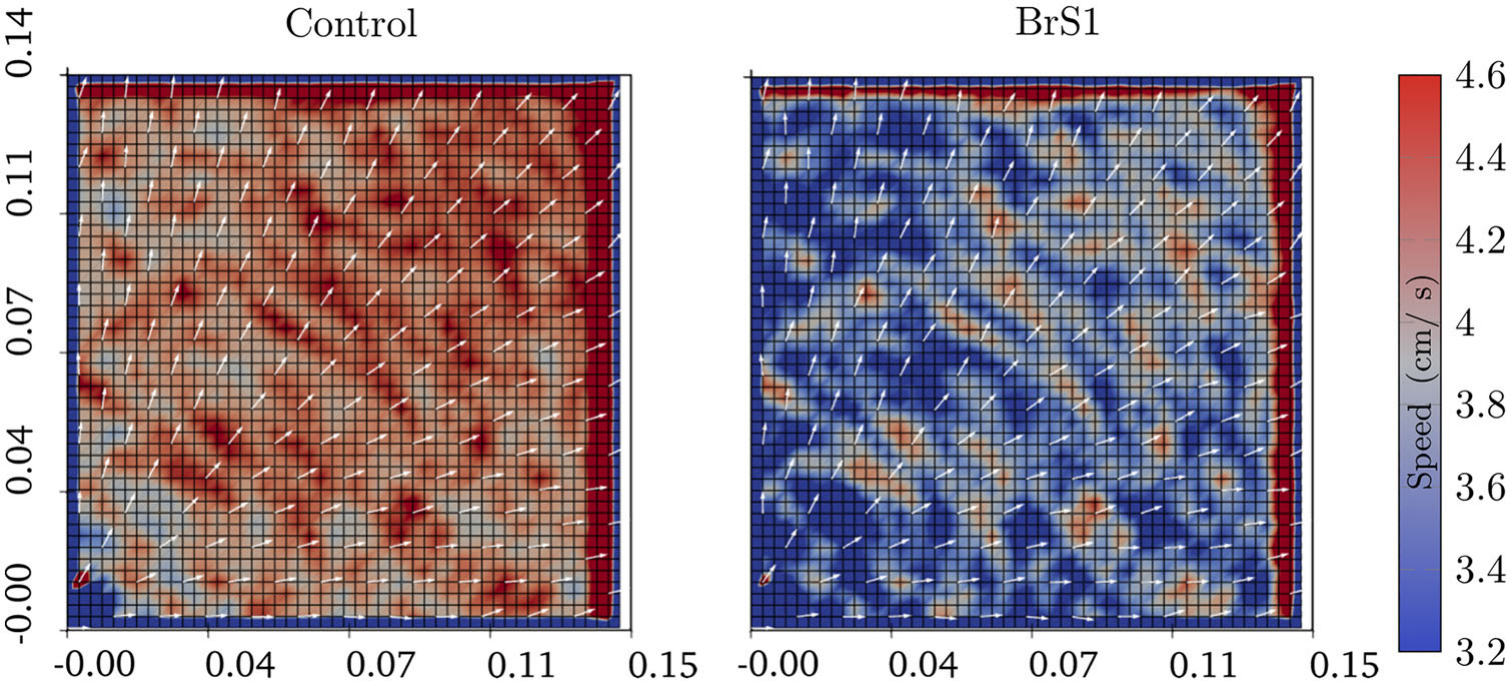
Local conduction speed map comparison Comparison of the local conduction speed vector field of the healthy tissue (Control, left panel) and the tissue affected by BrS1 (right panel). In both cases, the simulations were performed considering 30% of AL hiPSC‐CMs inside the well. To enhance clarity, only 20% of the vector field arrows are displayed. The colour scale shows both the local and global reduction of the BrS1 conduction velocity.

The reduction in the average and local conduction velocity is critical, as it facilitates the formation of re‐entrant circuits, which are central to the development of life‐threatening arrhythmias such as ventricular fibrillation. The localized slowing of conduction, combined with the electrical heterogeneity of the tissue, further contributes to the increased susceptibility to arrhythmias observed in BrS patients. These findings align with previous studies that have highlighted the impact of Na^+^ channel dysfunction on electrical propagation and the onset of arrhythmic events in this condition.

### Current blocker effects

In this study, we also analysed the effects of four drugs – mexiletine, nifedipine, dofetilide and E‐4031 – on ion channel dynamics using an *in silico* framework, comparing the results to experimental data from Abbate et al. (2018) and Millard et al. (2018). To quantify drug‐induced modifications, we employ the mathematical model detailed in the Methods. This model captures the dose‐dependent influence of ion channel blockers and openers on current properties through Eqs (12) and (13). These formulations allow us to predict both agonistic and antagonistic drug effects by incorporating parameters such as drug concentration, Hill coefficients and maximum drug efficacy.

Building on this framework, we focus on the implications of drug interactions specifically for the VL and AL hiPSC‐CMs. Since our interest is focused on the effect of the drug, a standard differentiation protocol is assumed in our simulations, while the effects of different heterogeneity degrees are excluded. Following the description of the standard differentiation protocol analysed in Altomare et al. (2023), in the simulated culture we set a value of 30% of AL hiPSC‐CMs.

The following results highlight the interplay between the modelled drug effects and the experimental observations, shedding light on the predictive power of the *in silico* approach in understanding ion channel behaviour under the influence of a drug.

#### Mexiletine

Mexiletine is an anti‐arrhythmic drug, multi‐channel blocker, structurally related to lidocaine, primarily known for being both a voltage‐gated Na^+^ channel ($I_{\mathrm{Na}}$) blocker and an inhibitor of late Ca^2+^ currents ($I_{\mathrm{CaL}}$).

Based on Jæger et al. (2021b), current blocks was modelled in both Paci2020 and Botti2024 ionic models following Eq. (12) with E=−1, where $EC_{50,I_{\mathrm{Na}}}=201\;\mu M$ and $EC_{50,I_{\mathrm{CaL}}}=963\;\mu M$ are taken directly from the literature (Jæger et al., 2021c) and the Hill coefficients are estimated in Jæger et al. (2021b) from fitting dose‐dependent block data (H=1).

By inhibiting Na^+^ influx during phase 0 of the AP, mexiletine reduces the depolarization rate, leading to a shortened APD, as depicted in Fig. 12. Furthermore, partial block of the Na^+^ influx also reduces the average conduction velocity, equal to 3.2768 cm*/*s, with respect to the one in healthy dishes. Conversely, by reducing Ca^2+^ entry, mexiletine contributes to the stabilization of membrane repolarization, which may prevent early afterdepolarizations that can trigger arrhythmias.

**Figure 12:**
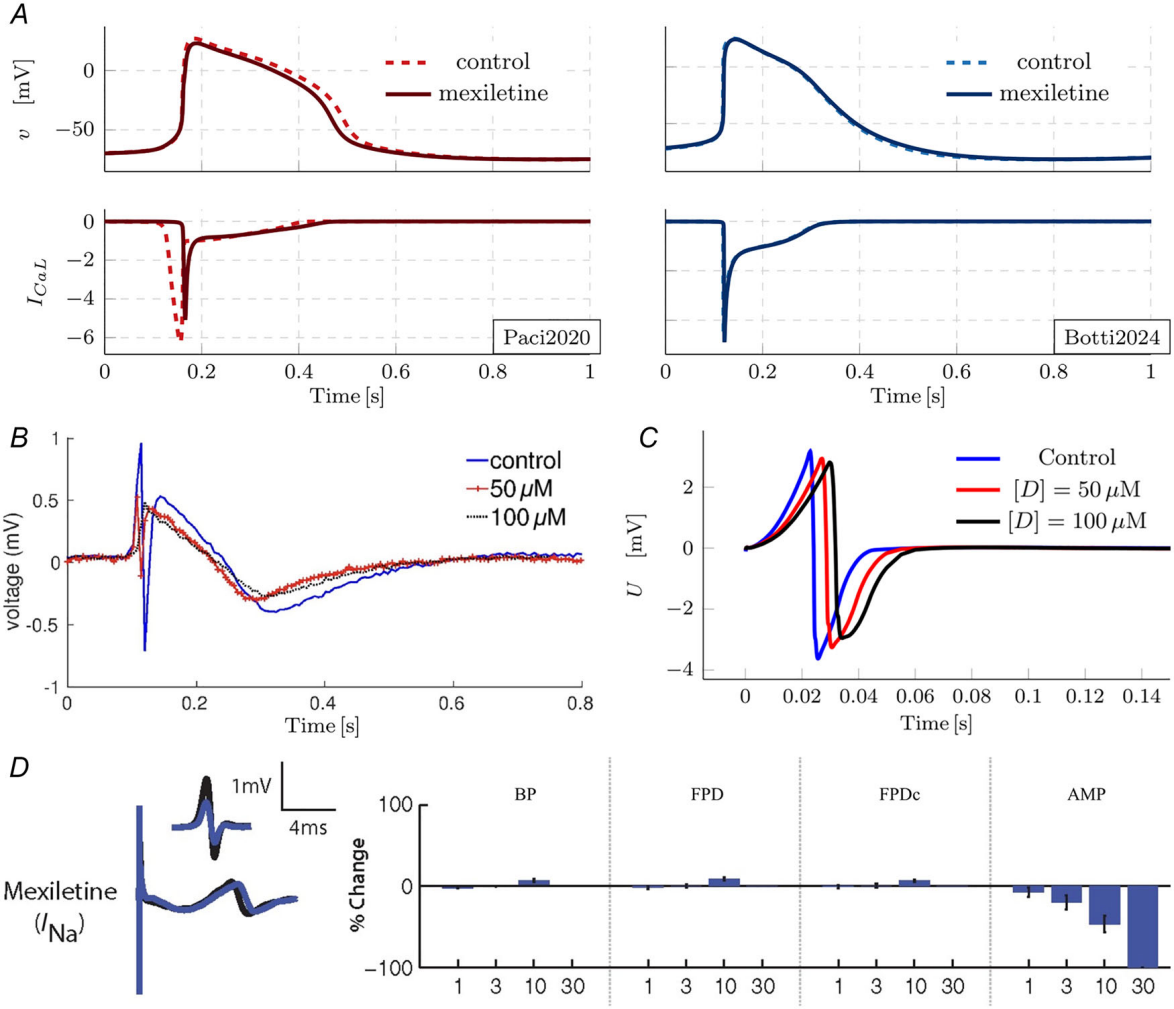
Mexiletine effect *A*, control (dashed line) and drug effects (continuous line) on v (top) and $I_{\mathrm{CaL}}$ current (bottom) for phenotype‐specific ionic models (Paci2020 VL dynamics on the left, Botti2024 AL dynamics on the right). *B*, experimental data from Millard et al. (2018); and *C*, simulated results: black lines correspond to control conditions, blue lines to mexiletine effect. *D*, experimental data from Millard et al. (2018). Left: experimental FP waveforms (black line corresponds to control conditions, blue line corresponds to mexiletine effect). Right: percentage of change for different drug concentrations of the following biomarkers: beat period (BP), FP duration (FPD), FPD measurements rate‐corrected (FPDc) using the Fridericia correction (Fridericia, 2003) and spike amplitude (AMP).

The single‐cell dynamic, depicted in Fig. 12, highlights the APD reduction effect as a consequence of $I_{\mathrm{CaL}}$ reduction. In the Paci2020 model, $\mathrm{Int}(I_{\mathrm{CaL}})$ equals 207 and 309 (pA·ms)/pF in the superfused and control conditions, respectively, computed following Eq. (14). In the Botti2024 model, the reduction corresponds to 200 compared to 204 (pA·ms)/pF. In ventricular CMs, $I_{\mathrm{CaL}}$ is the major inward current responsible for maintaining the AP plateau, making it a major determinant of AP duration. Ca^2+^ influx during the plateau phase initiates the process of calcium‐induced calcium release. The effect $I_{\mathrm{CaL}}$ block through mexiletine results in a reduction of Ca^2+^ influx and a decrease of the plateau phase (and APD). In contrast, blocking $I_{\mathrm{CaL}}$ appears not to impact AP morphology in AL hiPSC‐CMs, and this is in line with the observation that the dynamics of $I_{\mathrm{CaL}}$ primarily contributes to the plateau phase, mainly absent in the triangular shape of AL hiPSC‐CMs.

Therefore, this compound mainly impacts the depolarizing phase of cells, as can be observed in the measurements in Abbate et al. (2018). We simulated *in silico* the current block effect when considering different drug concentrations ([*D*] = 50 µM and [*D*] = 100 µM) and the same dose‐dependent effects are observed in both experiments and simulations taken from Abbate et al. (2018) as depicted in the comparison in Fig. 12. As expected, mexiletine produced a concentration‐dependent decrease in the amplitude, with minimal effects on FP duration at the highest concentrations, confirming the data provided in Millard et al. (2018) and shown in Fig. 12*D*. Spike amplitude provides an indirect measure of drug effects on the propagating AP upstroke, confirming the quantitative reduction of the local conduction velocity stated above.

#### Dofetilide

Dofetilide is an anti‐arrhythmic drug that selectively blocks the rapid component of the delayed rectifier K^+^ current ($I_{\mathrm{Kr}}$) in cardiac myocytes. Based on Abbate et al. (2018), current blocks was modelled in both Paci2020 and Botti2024 ionic models following Eq. (13), where IC_50_ = 5 nM, and the Hill coefficient *H* is equal to 1. We simulated *in silico* the effect of current block when considering [*D*] = 10 nM.

By inhibiting $I_{\mathrm{Kr}}$, dofetilide prolongs the repolarization phase (phase 3) of the cardiac AP, leading to a prolongation of the APD. This effect is clearly reproduced in both the VL and AL hiPSC‐CM ionic models and shown in Fig. 13. In the Paci2020 model, $\mathrm{Int}(I_{\mathrm{Kr}})$ equals 48 and 128 (pA·ms)/pF in the superfused and control conditions, respectively, computed following Eq. (14). In the Botti2024 model, the reduction corresponds to 27 compared to 68 (pA·ms)/pF. This action results in a prolongation of the QT interval on the ECG, corresponding to a prolongation of the FP duration, as seen in both the experimental results presented in Abbate et al. (2018) and simulation curves (see Fig. 13). This action results in a prolongation of the QT interval on the ECG, corresponding to a prolongation of the FP duration, as seen in both the experimental results presented in Abbate et al. (2018) and simulation curves (see Fig. 13). The same dose‐dependent delay of the repolarization wave can be noted in Fig. 13*B*, and confirmed by our simulation shown in Fig. 13*C*.

**Figure 13:**
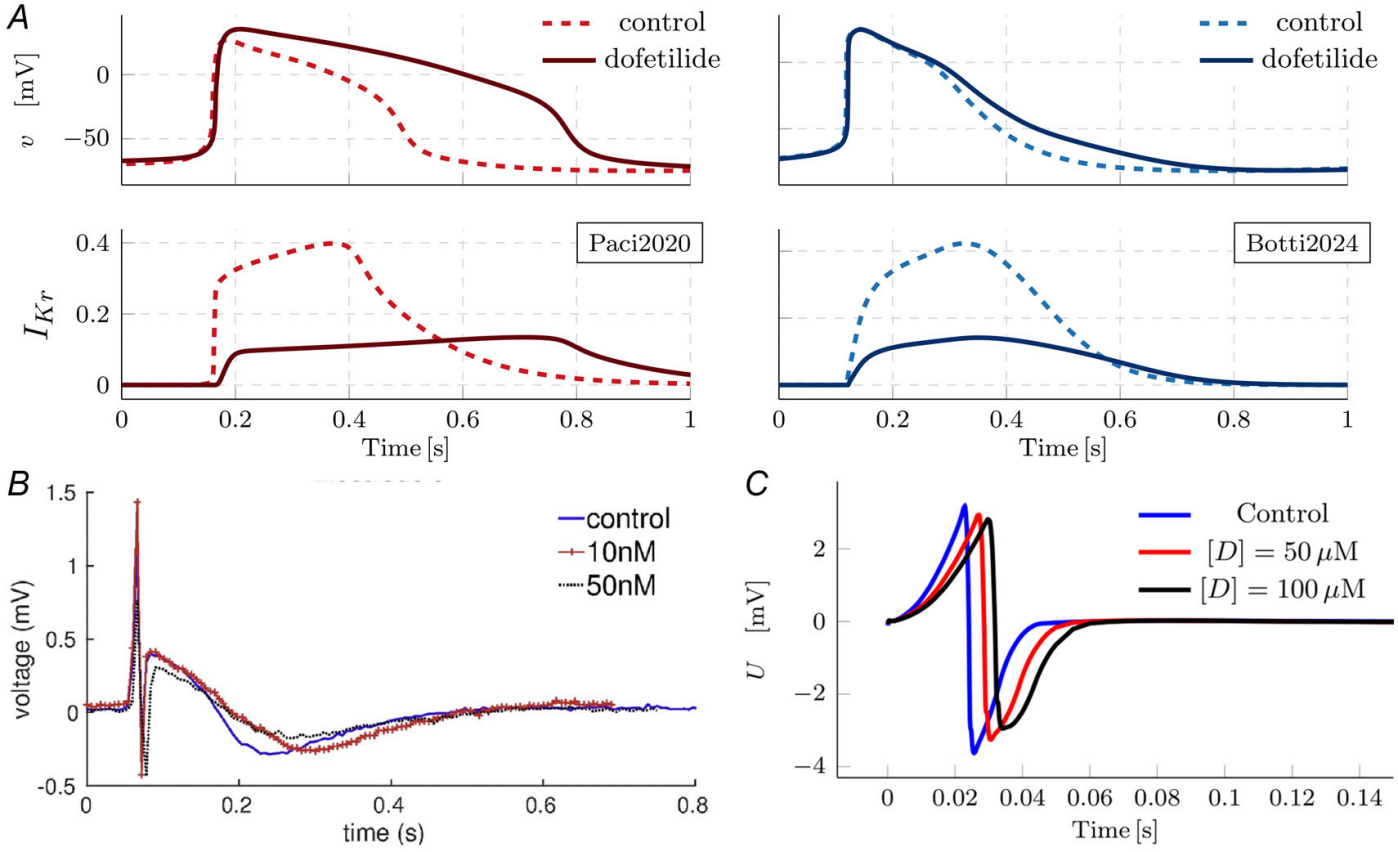
Dofetilide effect *A*, control (dashed line) and drug effects (continuous line) on v (top) and $I_{\mathrm{Kr}}$ current (bottom) for phenotype‐specific ionic models (Paci2020 VL dynamics on the left, Botti2024 AL dynamics on the right). *B*, experimental data from Abbate et al. (2018): blue line corresponds to control conditions, red line to 10 nM and black line to 50 nM. *C*, simulated results: blue line corresponds to control conditions, and red line to dofetilide effect.

#### Nifedipine

Nifedipine is a Ca^2+^ channel blocker, primarily acting on $I_{\mathrm{CaL}}$ in cardiac and vascular smooth muscle cells. Based on Paci et al. (2018), current blocks was modelled in both Paci2020 and Botti2024 ionic models following Eq. (13), where IC_50_ = 38 nM, and the Hill coefficient *H* is equal to 1. We simulated *in silico* the effect of current block when considering [*D*] = 10 nM.

By inhibiting Ca^2+^ influx through these channels during phase 2 of the cardiac AP, nifedipine shortens the plateau phase of the AP, shortening APD and triangulating the AP profile. In the Paci2020 model, $\mathrm{Int}(I_{\mathrm{CaL}})$ equals 211 and 309 (pA·ms)/pF in the superfused and control conditions, respectively, computed following Eq. (14). In the Botti2024 model, the reduction corresponds to 199 compared to 204 (pA·ms)/pF. In single‐cell simulations, when considering ionic models used in the MEA simulation, the $I_{\mathrm{CaL}}$ block‐induced APD shortening was evident in VL hiPSC‐CMs, as shown in Fig. 14. The effects of nifedipine on VL hiPSC‐CMs confirm the results reported in Paci et al. (2020). Similarly, the effect of nifedipide ion AL hiPSC‐CMs is almost imperceptible, mirroring the mexiletine's $I_{\mathrm{CaL}}$ block effect.

**Figure 14:**
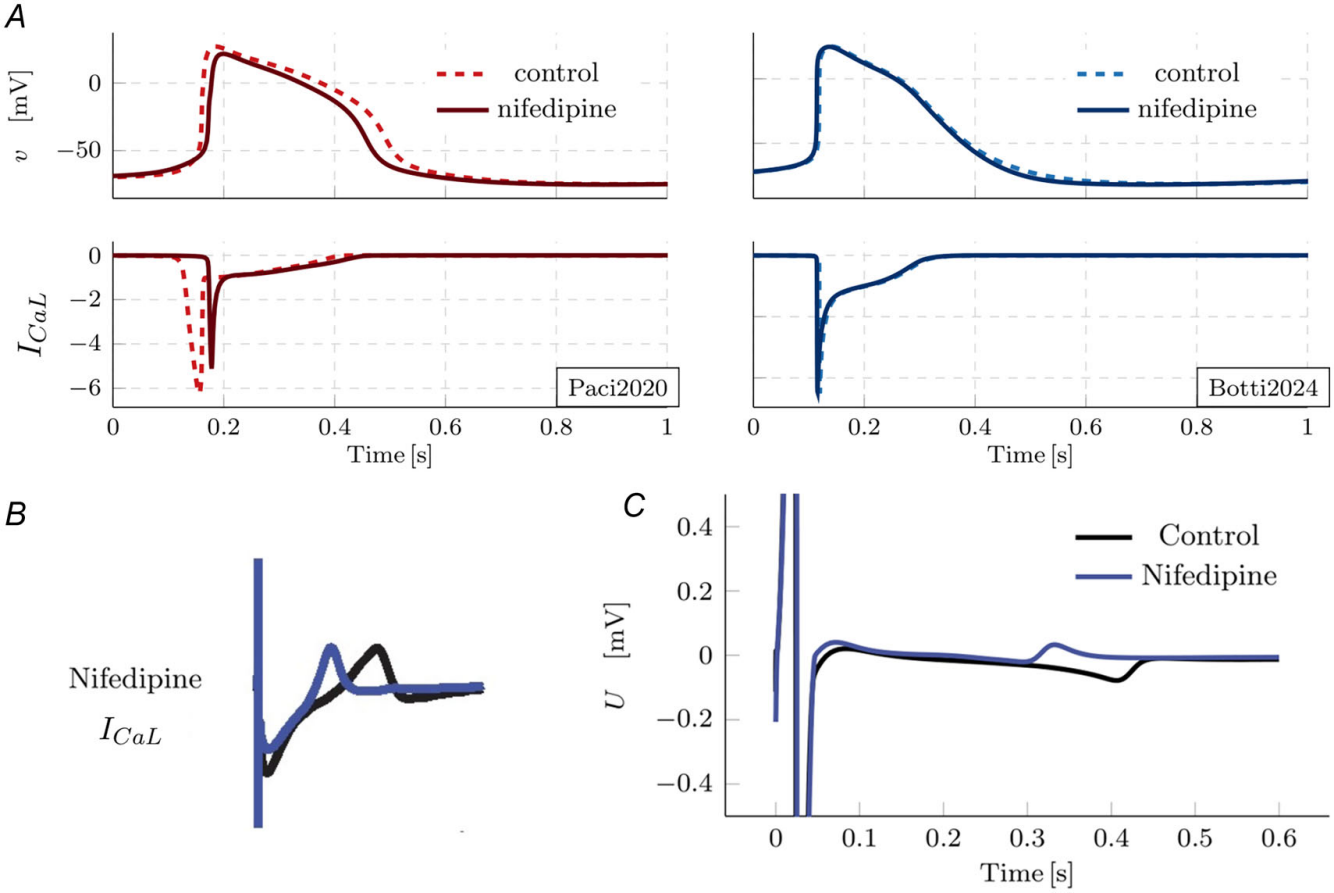
Nifedipine effect *A*, control (dashed line) and drug effects (continuous line) on v (top) and $I_{\mathrm{CaL}}$ current (bottom) for phenotype‐specific ionic models (Paci2020 VL dynamics on the left, Botti2024 AL dynamics on the right). *B*, experimental data from Millard et al. (2018); and *C*, simulated results: black lines correspond to control conditions, and blue lines to nifedipine effect.

Furthermore, the $I_{\mathrm{CaL}}$ blocking drug nifedipine produced concentration‐dependent reductions of the beat period and shortening of FP duration, with minimal effects on the amplitude, as shown both in experimental profiles and simulated FP in Fig. 14.

#### E‐4031

E‐4031 is a potent and selective blocker of the rapid component of the $I_{\mathrm{Kr}}$ in CMs. Based on Paci et al. (2018), current blocks was modelled in both Paci2020 and Botti2024 ionic models following Eq. (13), where IC_50_ = 100 nM, and the Hill coefficient H is equal to 1. We simulated *in silico* the effect of current block when considering [*D*] = 100 nM.

By inhibiting $I_{\mathrm{Kr}}$, E‐4031 prolongs the repolarization phase (phase 3) of the cardiac AP, leading to a marked increase in APD and inducing a strong APD prolongation. This effect is clearly reproduced in both the VL and AL hiPSC‐CM ionic models and shown in Fig. 15. In the Paci2020 model, $\mathrm{Int}(I_{\mathrm{Kr}})$ equals 71 and 128 (pA·ms)/pF in the superfused and control conditions, respectively, computed following Eq. (14). In the Botti2024 model, the reduction corresponds to 38 compared to 68 (pA·ms)/pF. A similar APD prolongation was obtained *in vitro* using E‐4031 (Ma et al., 2011), which reduced K^+^ efflux through the cell membrane, thus prolonging the AP. The *in silico* effects of E‐4031 on VL hiPSC‐CMs confirm the results reported in Paci et al. (2020). Similarly, the effect of E‐4031 on AL hiPSC‐CMs is almost imperceptible, mirroring the dofetilide's $I_{\mathrm{CaL}}$ block effect.

**Figure 15:**
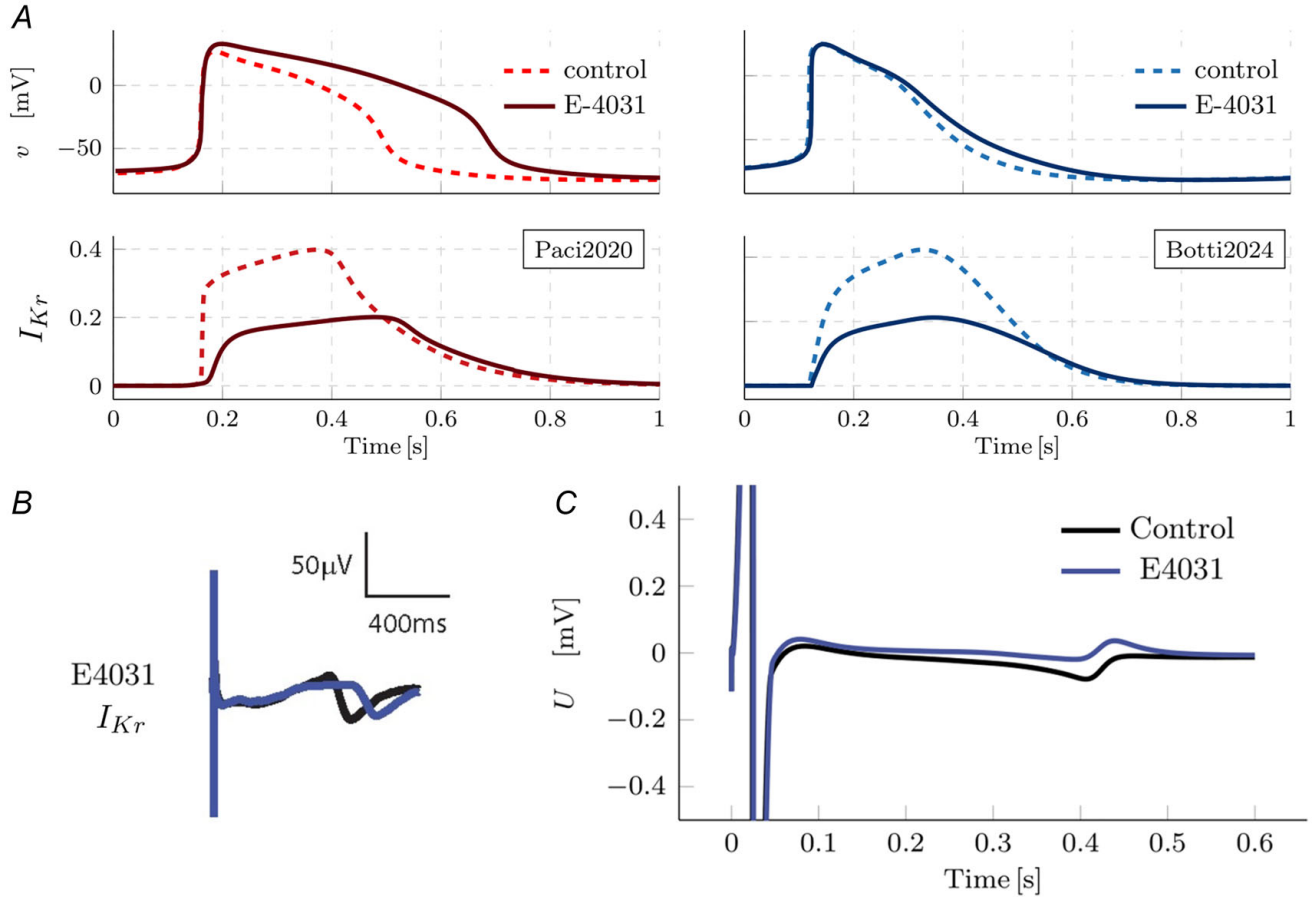
E‐4031 effect *A*, control (dashed line) and drug effects (continuous line) on v (top) and $I_{\mathrm{Kr}}$ current (bottom) for phenotype‐specific ionic models (Paci2020 VL dynamics on the left, Botti2024 AL dynamics on the right). *B*, experimental data from Millard et al. (2018); and *C*, simulated results: black lines correspond to control conditions, and blue lines to E‐4031 effect.

This prolongation of the AP results in an extended QT interval on the ECG and a prolongation of the FP duration, as depicted in Fig. 15, where we show the comparison between experimental data from Millard et al. (2018) and the FP measured by one electrode in our MEA simulation.

## Discussion

In this paper, we presented a comprehensive mathematical framework for modelling the electrophysiological properties of hiPSC‐CM cultures recorded via MEA devices. Our model builds on the established bidomain equations, widely used in studies of cardiac electrical activity, and incorporates a model of electrodes that couples with the bidomain framework. This approach facilitates a robust simulation environment for analysing MEA recordings.

To enhance the utility of hiPSC‐CMs in drug development, reliable MEA recordings are essential. MEAs provide a sensitive and non‐invasive approach to studying CM electrophysiology, offering detailed spatio‐temporal resolution. However, their application with human CMs faces challenges, such as the structural complexity of embryoid bodies (Zhu et al., 2017). Over the past decade, MEAs have gained popularity in iPSC‐based disease modelling and functional phenotyping. The latest multi‐well MEA systems support higher throughput, making them ideal for large‐scale functional and pharmacological studies. Additionally, because MEA recording is non‐destructive, researchers can observe the same hiPSC‐CM cultures over extended periods, yielding insights into long‐term maturation (Clements & Thomas, 2014). Combining hiPSC‐CM monolayer cultures with MEA systems allows for accurate, real‐time measurement of electrophysiological properties, making them valuable tools for studying human heart development, disease mechanisms, and drug efficacy and toxicity (Zhu et al., 2017; Raphel et al., 2017). MEA‐recorded signals, the FPs, provide indirect measures of cardiac AP, capturing depolarization and repolarization phases. However, FP analysis remains challenging due to the variability of FP traces and the limited studies available compared to direct AP recordings. The variability of FPs arises from several factors, including differences in cell density, culture conditions, electrode placement and the inherent heterogeneity of hiPSC‐CM preparations. These factors influence the amplitude, duration and morphology of the recorded signals, making it difficult to standardize and interpret results across experiments.

### Chamber‐specific hiPSC‐CM ionic models and heterogeneous VL/AL MEA

From a mathematical perspective, existing simulations are based on the tissue bidomain model for ventricular‐like hiPSC‐CMs, but they lack specific modelling of MEA device interactions and tissue phenotypical heterogeneity. At the single‐cell level, the classification of AL, VL and nodal‐like phenotypes is based on (i) comparisons of their APs with those of adult CMs and (ii) the triangular shape of their APs (Chapotte‐Baldacci et al., 2024, 2023). The distinction between AL and VL is usually based on biomarkers representing the repolarization slope; however, significant variations in AP morphology can still be observed within a single phenotype. The Paci2013 model (Paci et al., 2013) introduced AL and VL ionic models, highlighting key differences in ion channel conductances and Ca^2+^ current dynamics. However, this model was based on recordings from a mixed population of nodal, and ventricular‐ and atrial‐like hiPSC‐CMs, and it reproduced phenotypical heterogeneity by scaling parameters rather than incorporating phenotype‐specific currents. In recent years, the use of maturation techniques developed has highlighted the chamber‐specific AP phenotype of the cells (Devalla et al., 2015). In this direction, the latest version of the Paci2020 hiPSC‐CM ionic model presented in Paci et al. (2020) provides the most recent version of a VL hiPSC‐CM model. Among the different maturation techniques, dynamic clamp (DC) overcomes the immature electrical properties of hiPSC‐CMs through the injection of a virtual $I_{K1}$ current in a real‐time mode, as $I_{K1}$ plays a significant role in maintaining the resting membrane potential and stabilizing the electrical excitability of cardiomyocytes (Horv´ath et al., 2018). The AL datasets presented in Altomare et al. (2023), recorded under DC, have been recently used to build a novel atrial‐specific ionic model, Botti2024, which also takes into account atrial‐specific currents, such as $I_{\mathrm{Kur}}$ and $I_{\mathrm{KCa}}$ (Maleckar et al., 2009; Skibsbye et al., 2016).

In our bi‐dimensional framework, we accounted for the heterogeneity in hiPSC‐CM phenotypes by adopting the Paci2020 ionic model (Paci et al., 2020), which captures the electrophysiological properties of VL hiPSCs. Additionally, we incorporated the recently published Botti2024 model for AL hiPSCs (Botti et al., 2024), extending the utility of Paci's ionic modelling framework to encompass various phenotypes. Both of the models were used considering the spontaneous protocol, and the solid lines in Fig. 3 qualitatively reflect the behaviour presented in Lemme et al. (2018) of AP traces recorded in control and retinoic acid treated engineered heart tissue (EHT). Notably, the ionic term in our bidomain model can be readily adapted to other established electrophysiological models of hiPSC‐CMs, such as those by Koivumaäki et al. (2018) and Kernik et al. (2019). Detailed comparative analyses of these models have been previously discussed, as referenced in recent reviews (Trayanova et al., 2024; Trancuccio, 2023; Paci et al., 2021). In this paper, we focus primarily on VL and AL mixed configurations and diseased states. Recent advances in computational atrial models have significantly enhanced our understanding of how intrinsic structural and electrophysiological heterogeneity predispose atrial tissue to arrhythmias (Niederer et al., 2019). These insights underline the potential value of investigating the MEA computational model within a framework that emphasizes a predominant atrial phenotype.

### Parameter calibration

#### MEA conductivities

After defining phenotype heterogeneity, we also calibrated the intra‐ and extracellular conductivities of our MEA model (see Eq. 5). Previous studies have demonstrated that bidomain simulations in small hiPSC‐CM populations often exhibit negligible or absent repolarization waves (Abbate et al., 2018), especially when standard parameter values are used. Research has shown that reducing the intracellular conductivity $\sigma^{i}$ can enhance the visibility of repolarization waves (Jæger et al., 2021c); however, this reduction alone may weaken the extracellular signal. By additionally decreasing the extracellular conductivity $\sigma^{e}$, our model produces realistic amplitudes for both depolarization and repolarization waves, aligning more closely with physiological observations.

#### Drug modelling

The two principal methodologies for modelling the impact of drugs on ion currents in CMs are based on Markov models and IC_50_ models. Markov models, as described in Clancy & Rudy (1999), provide a highly detailed representation that closely corresponds to the molecular structure and biophysical characteristics of ion channels in certain cases. However, these models demand extensive datasets for each individual current, which are often unavailable. Ideally, Markov models would be parameterized using data derived from single‐channel recordings, but such data are generally scarce. In contrast, the IC_50_‐based approach applied in the study of Zemzemi et al. (2013) is less complex and relies on fewer biomarkers for estimation. It is important to note that reported IC_50_ values in the literature can vary considerably, a variability that may stem from the absence of standardized protocols for IC_50_ measurement, as suggested by Lee et al. (2019). We have addressed parameter selection for each drug in the corresponding subsections of the Results.

### Impact of tissue heterogeneity on activation/repolarization dynamics and conduction velocity

Clark et al. (2024) emphasized that the inherent electrophysiological heterogeneity of these cells imposes significant limitations on the depth of insights that can be extracted from even well‐designed experiments. This heterogeneity affects various electrophysiological properties, influencing the conduction dynamics and repolarization patterns in complex ways.

A particularly noteworthy finding is the spatial variation in conduction velocity within heterogeneous tissues, despite relatively uniform activation time. This suggests that intracellular interactions and tissue architecture influence electrical signal propagation in a manner distinct from homogeneous tissues. Our results indicate that conduction velocity increases as the proportion of AL hiPSC‐CMs in the tissue rises. This is a crucial observation, as it implies that analysing conduction velocity could help in identifying the predominant cellular phenotype within a tissue sample.

Contrary to our findings, some studies (O’ Brien et al., 2022; Goldfracht et al., 2020) report that the conduction velocity of VL EHT is lower than that of retinoic acid‐treated AL EHT. However, this is not necessarily a discrepancy. Homogeneous EHT structures differ significantly from MEA syncytia, and it is plausible that fibre structures and cellular clustering also contribute to the observed conduction differences within EHT models.

Moreover, integrating conduction velocity analysis with repolarization time isochrones may provide deeper insights into tissue heterogeneity, offering a powerful tool to quantify and characterize variability in electrophysiological properties. The impact of heterogeneity is also evident in repolarization time and associated APD maps. As the level of heterogeneity increases, APD isochrones become less smooth, reflecting irregular and chaotic recovery patterns.

This phenomenon is clearly illustrated in fig. 5 of Riedel et al. (2014), where isochronal time maps from a typical MEA chamber depict AT (activation), ARI (a surrogate for APD) and conduction velocity. While AT maps exhibit relatively smooth isochrones, conduction velocity and APD maps display significant non‐uniformity, emphasizing the intricate effects of tissue heterogeneity on electrical activity. In these plots, AT and ARI are expressed in milliseconds, whereas thconduction velocity is reported in cm/s.

These findings underline the importance of considering structural and cellular heterogeneity when interpreting electrophysiological data, as even subtle variations in tissue composition can significantly impact electrical conduction and repolarization dynamics.

### Impact of tissue heterogeneity and Brugada syndrome

BrS is a genetic cardiac arrhythmia disorder associated with a heightened risk of sudden cardiac death. BrS was first described by Martini et al. (1989) and is characterized by right bundle branch block and ST segment elevation, which can lead to ventricular arrhythmias such as re‐entrant ventricular tachycardia and ventricular fibrillation. BrS has gained significant clinical attention due to its prevalence in younger, otherwise healthy individuals, emphasizing the critical need for understanding its underlying mechanisms.

By disrupting the intricate balance of inward and outward ionic currents, both SCN5A and LTCC mutations can contribute to the hallmark ECG and arrhythmic features of BrS. However, SCN5A mutations remain the predominant genetic marker associated with the syndrome, while LTCC‐related cases require further exploration to fully elucidate their clinical and molecular implications (Tse et al., 2016). In this study, we have analysed both BrS1 cases associated with SCN5A mutations by using insights from existing computational models (Scacchi et al., 2023), and BrS2 cases linked to LTCC mutations through a bottom‐up approach (Burashnikov et al., 2010).

The most commonly reported consequence of SCN5A mutations is a reduction in the inward Na^+^ current ($I_{\mathrm{Na}}$) flow, which results in an imbalance between inward and outward ionic currents during the transient repolarization phase of the AP. This disruption can manifest through a diminished $I_{\mathrm{Na}}$ and L‐type Ca^2+^ current ($I_{\mathrm{CaL}}$) or through an increase in the outward K^+^ current ($I_{\mathrm{to}}$). These ionic shifts create a pronounced notch and the loss of the AP dome, leading to the formation of a substrate that is prone to the initiation of arrhythmias. Such heterogeneous electrical substrates, as identified in Pappone et al. (2018), are thought to facilitate re‐entrant circuits that trigger malignant ventricular arrhythmias. Recent research, such as that by Scacchi et al. (2023), has examined both clinical and electrophysiological predictors of malignant ventricular tachyarrhythmia in BrS1 patients. These studies suggest that there may be a correlation between the extent of electrically altered regions within the right ventricle and the risk of fatal arrhythmias. However, these insights may not fully apply to hiPSC‐CM‐based *in silico* models, where the heterogeneous differentiation of the cells makes it challenging to map specific substrates to anatomical regions, such as the right ventricle.

Models of BrS1 incorporating SCN5A mutations, which impair the Na^+^ current ($I_{\mathrm{Na}}$), demonstrate pronounced conduction abnormalities, particularly in the right ventricle (RV) compared to the left ventricle (LV). In these cases, the reduced conduction velocity arises from a slower upstroke velocity of phase 0 in the AP, supporting the depolarization theory. This theory posits that the slowing of AP propagation is a key mechanism driving arrhythmogenesis in BrS1 (Tse et al., 2016).

In contrast, pathogenic variants in BrS that do not affect $I_{\mathrm{Na}}$ follow a different electrophysiological mechanism. Mutations that reduce the Ca^2+^ current or enhance the K^+^ current do not alter the phase 0 upstroke velocity and, consequently, do not reduce the conduction velocity. Instead, these variants primarily influence the repolarization phase, shortening the AP plateau duration (Tse et al., 2016). This highlights the diverse mechanisms underlying BrS2, with conduction disturbances being specific to *SCN5A*‐related mutations (Tse et al., 2016).

### Drugs effects on heterogeneous MEA recordings

The application of computational modelling to investigate the effects of drugs on diseased and treated cardiac tissues has garnered increasing attention in the field of cardiac electrophysiology. This approach is particularly relevant given the challenges associated with traditional drug development, such as high financial costs and regulatory constraints (Macheras & Iliadis, 2016; Mirams et al., 2012; Mulder et al., 2018). As a result, modelling has emerged as a valuable tool for optimizing existing pharmacological therapies and predicting drug interactions, especially in arrhythmic conditions (Brennan et al., 2009).

Previous studies have demonstrated the efficacy of anti‐arrhythmic drugs such as mexiletine in managing ventricular arrhythmias and BrS, as well as in shortening the QT interval in patients with long QT syndrome type 3 (LQT3) (Mazzanti et al., 2016; Burashnikov et al., 2010). Computational models have successfully replicated the dose‐dependent effects of mexiletine on Na^+^ channel function, corroborating its clinical efficacy (Matsa et al., 2011).

Similarly, dofetilide's role in delaying repolarization has been extensively studied both experimentally and *in silico*. The drug's selective inhibition of the $I_{\mathrm{Kr}}$ current makes it a crucial agent in the treatment of atrial fibrillation and flutter (Hancox et al., 2008). Our findings align with previous computational studies demonstrating how dofetilide‐induced AP prolongation can predispose cardiomyocytes to early afterdepolarizations (EADs), a known pro‐arrhythmic risk (Zhao & Li, 2019).

Regarding Ca^2+^ channel modulation, nifedipine has been widely recognized for its dual role in lowering blood pressure and modulating cardiac excitability. Its ability to reduce Ca^2+^ overload has been well documented in ischaemic and failing hearts (Hayashi, 2000). Our simulations support these findings, reinforcing the potential of nifedipine in preventing arrhythmogenesis by stabilizing intracellular Ca^2+^ dynamics.

E‐4031, a selective blocker of $I_{\mathrm{Kr}}$, has served as a key pharmacological tool for investigating the electrophysiological basis of arrhythmias, particularly in the context of long QT syndrome (LQTS). Previous computational studies have illustrated how E‐4031 prolongs APD and induces EADs, thereby increasing the risk of life‐threatening arrhythmias (Grandi et al., 2010). Our results are consistent with these observations, further validating the concept of repolarization reserve and its role in maintaining stable cardiac rhythms under pharmacological stress.

Despite inherent limitations, multiscale models integrating ionic dynamics with tissue‐level electrophysiology provide a robust framework for assessing drug responses. As demonstrated in prior studies (Passini et al., 2017), such models enhance our understanding of MEA‐acquired signals and facilitate the advancement of computational drug testing methodologies. The findings of this study contribute to this growing body of literature, underlining the translational potential of *in silico* approaches in cardiac pharmacology

### Alternative cell‐by‐cell tissue models

Finally, our model assumes that the intracellular (I), cell membrane (M) and extracellular (E) spaces are uniformly distributed throughout the simulated domain. While this simplification has been effective in computational cardiology for millimetre‐scale resolutions over decades, it becomes less accurate at the micrometre scale of individual cells. At this level, more detailed approaches such as the extracellular–membrane–intracellular (EMI) model, which explicitly separates the computational domains for the E, M and I spaces, offer improved precision (Bécue et al. 2017; Jæger & Tveito, 2022; Tveito et al., 2017). However, despite their accuracy at subcellular scales, these methods are computationally intensive. In this study, the bidomain model was selected due to its efficiency in simulating larger tissue geometries while maintaining computational feasibility. While the EMI model could theoretically provide more accurate results, it would probably require significantly higher computational resources, especially at the cell‐by‐cell scale. Published studies have reported that EMI simulations typically require a runtime several times longer than bidomain simulations for comparable domains. Furthermore, EMI models often necessitate fine‐grained mesh discretization, dramatically increasing memory and computational costs. Future improvements could focus on optimizing the code by implementing GPU‐based parallelization, advanced preconditioning techniques for solving the large linear systems, and adaptive mesh refinement to balance accuracy and efficiency. Nonetheless, for the purposes of this study, the bidomain model offers a well‐established and computationally efficient framework that balances accuracy and computational practicality for tissue‐level simulations.

## Conclusions

In this study, we have developed a robust computational framework that integrates MEA‐based models with phenotype‐specific ionic models for hiPSC‐CMs, offering a detailed platform for *in silico* drug testing and cardiac disease simulation. By combining experimental MEA data with predictive mathematical models, we have successfully evaluated the effects of four drug compounds on hiPSC‐CMs, demonstrating the model's capacity to capture nuanced electrophysiological responses and validate experimental findings. This dual approach not only strengthens the accuracy of *in silico* predictions but also underlines the relevance of MEA systems in testing drug compounds in a non‐invasive, high‐throughput environment.

Our exploration of tissue heterogeneity in hiPSC‐CMs and its impact on conduction velocity provides critical insights into how cellular diversity influences electrophysiological properties at the tissue level. This consideration is essential for developing more effective, patient‐specific treatments, especially for conditions where conduction anomalies play a central role.

Furthermore, the framework's flexibility allowed us to simulate the electrophysiological behaviour associated with BrS, illustrating its potential to replicate pathological dynamics observed in adult CMs. This validation supports the utility of hiPSC‐CMs as a reliable model for studying inherited arrhythmias and highlights the ability of MEA‐based models to simulate complex disease states, an advancement particularly useful for preclinical drug screening.

Overall, this study demonstrates that combining hiPSC‐CM cultures with MEA‐based computational models hold significant promise for advancing cardiac drug testing and disease modelling. By providing a high‐fidelity, reproducible and scalable approach, this framework has the potential to optimize drug development pipelines, reduce the need for animal testing and enhance therapeutic strategies for complex cardiac conditions. Future work may expand this model to capture additional disease phenotypes, further increasing its utility as a versatile tool in personalized pharmacology.

### Limitations

Among the limitations of our work, we mention the lack of newly acquired experimental data, which will be a priority in subsequent studies to validate and refine the model. Additionally, we have considered only isotropic tissue, since no preferential conduction direction is shown in MEA recordings. Future work could enhance our modelling approach by incorporating a quantitative analysis of FP, contingent on defining precise biomarkers. Furthermore, we only considered a uniform random distribution of AL and VL cells. Other distributions could be investigated as well, but we do not expect this factor to significantly affect our results.

## Additional information

## Competing interests

The authors declare that they have no competing interests.

## Author contributions

The study was conceived and designed by S.B., L.F.P. and R.K. Method‐ology: S.B. Formal analysis: S.B. Writing the original draft: S.B. Writing the review and editing: S.B., L.F.P. and R.K. Supervision: L.F.P. and R.K. Conceptualization: L.F.P. and R.K. All authors have approved the final version of the manuscript and agree to be accountable for all aspects of the work.

## Funding

This project was supported by the Swiss National Science Foundation, SNSF grant 217025 (S.B.), the Italian Ministry of University and Research under Grant PRIN PNRR 2022, P2002B38NR (L.F.P.) and by the European High‐Performance Computing Joint Under‐taking (JU) EuroHPC under grant agreement No. 101172576 (MICROCARD‐2) (R.K, L.F.P.).

## Supporting information


Peer Review History

## Data Availability

The code is available upon request.
