# Supplementary material for: In silico modelling of multi‐electrode arrays for enhancing cardiac drug testing on hiPSC‐CM heterogeneous tissues
Source: J Physiol. 2025 May 11;604(19):8141–66. doi: 10.1113/JP287276 (PMC13628577; doi:10.1113/JP287276)
Supplement: Supplementary file 1 [file TJP-604-8141-s001.pdf]

The Journal of Physiology

In-silico Modeling of Multi-Electrode Arrays for Enhancing Cardiac Drug Testing on hiPSC-CMs Heterogeneous Tissues

Sofia Botti, Rolf Krause, and Luca Franco Pavarino
DOI: 10.1113/JP287276

Corresponding author(s): Sofia Botti (sofia.botti02@universitadipavia.it)

The following individual(s) involved in review of this submission have agreed to reveal their identity: Jussi Koivumäki (Referee #2)

Review Timeline:

Submission Date:	14-Nov-2024
Editorial Decision:	09-Dec-2024
Revision Received:	17-Feb-2025
Editorial Decision:	10-Mar-2025
Revision Received:	18-Mar-2025
Accepted:	25-Mar-2025

Senior Editor: Natalia Trayanova

Reviewing Editor: Christian Vergara

Transaction Report:

(Note: With the exception of the correction of typographical or spelling errors that could be a source of ambiguity, letters and reports are not edited. Depending on transfer agreements, referee reports obtained elsewhere may or may not be included in this compilation. Referee reports are anonymous unless the Referee chooses to sign their reports.)

Dear Dr Botti,

Re: JP-RP-2024-287276 "In-silico Modeling of Multi-Electrode Arrays for Enhancing Cardiac Drug Testing on hiPSC-CMs Heterogeneous Tissues" by Sofia Botti, Rolf Krause, and Luca Franco Pavarino

Thank you for submitting your manuscript to The Journal of Physiology. It has been assessed by a Reviewing Editor and by 2 expert referees and we are pleased to tell you that it is potentially acceptable for publication following satisfactory major revision.

Please advise your co-authors of this decision as soon as possible.

The referee reports are copied at the end of this email.

Please address all the points raised and incorporate all requested revisions or explain in your Response to Referees why a change has not been made. We hope you will find the comments helpful and that you will be able to return your revised manuscript within 2 months. If you require longer than this, please contact journal staff: jp@physoc.org. Please note that this letter does not constitute a guarantee for acceptance of your revised manuscript.

Your revised manuscript should be submitted online using the link in your Author Tasks: Link Not Available. This link is accessible via your account as Corresponding Author; it is not available to your co-authors. If this presents a problem, please contact journal staff (jp@physoc.org). Image files from the previous version are retained on the system. Please ensure you replace or remove any files that are being revised.

If you do not wish to submit a revised version of your manuscript, you must inform our journal staff (jp@physoc.org) or reply to this email to request withdrawal. Please note that a manuscript must be formally withdrawn from the peer review process at one journal before it may be submitted to another journal.

TRANSPARENT PEER REVIEW POLICY: To improve the transparency of its peer review process, The Journal of Physiology publishes online as supporting information the peer review history of all articles accepted for publication. Readers will have access to decision letters, including Editors' comments and referee reports, for each version of the manuscript, as well as any author responses to peer review comments. Referees can decide whether or not they wish to be named on the peer review history document.

ABSTRACT FIGURES: Authors are expected to use The Journal's premium BioRender account to create/redraw their Abstract Figures. Information on how to access this account is here: https://physoc.onlinelibrary.wiley.com/journal/14697793/biorender-access.

This will enable Authors to create and download high-resolution figures. If authors have used the free BioRender service, they can use the instructions provided in the link above to download a high-resolution version suitable for publication.

The link provided should only be used for the purposes of this submission. Authors will be charged for figures created on this account if they are not related to this manuscript submission.

LANGUAGE EDITING AND SUPPORT FOR PUBLICATION: If you would like help with English language editing, or other article preparation support, Wiley Editing Services offers expert help, including English Language Editing, as well as translation, manuscript formatting, and figure formatting at www.wileyauthors.com/eeo/preparation. You can also find resources for Preparing Your Article for general guidance about writing and preparing your manuscript at www.wileyauthors.com/eeo/prepresources.

RIGOUR AND REPRODUCIBILITY: We recommend authors consult The Journal's Rigour and Reproducibility requirements to ensure that their manuscript is suitable for publication: https://physoc.onlinelibrary.wiley.com/pb-assets/hub-assets/physoc/documents/TJP-Rigour-and-Reproducibility-Requirements.pdf

REVISION CHECKLIST:

Check that your Methods section conforms to journal policy: https://jp.msubmit.net/cgi-bin/main.plex?form_type=display_requirements#methods.

Check that data presented conforms to the statistics policy: https://jp.msubmit.net/cgi-bin/main.plex?form_type=display_requirements#statistics.

Upload a full Response to Referees file. To create your 'Response to Referees': copy all the reports, including any comments from the Senior and Reviewing Editors, into a Microsoft Word, or similar, file and respond to each point, using font or background colour to distinguish comments and responses and upload as the required file type.

Please upload two versions of your manuscript text: one with all relevant changes highlighted and one clean version with no

changes tracked. The manuscript file should include all tables and figure legends, but each figure/graph should be uploaded as separate, high-resolution files.

You may also upload:

- 'Potential Cover Art' for consideration as the issue's cover image
- Appropriate Supporting Information (Video, audio or data set: see https://jp.msubmit.net/cgi-bin/main.plex?form_type=display_requirements#supp).

We look forward to receiving your revised submission.

If you have any queries, please reply to this email and we will be pleased to advise.

Yours sincerely,

Natalia Trayanova
Senior Editor
The Journal of Physiology

REQUIRED ITEMS

- Include a Key Points list in the article itself, before the Abstract.

- Author photo and profile. First or joint first authors are asked to provide a short biography (no more than 100 words for one author or 150 words in total for joint first authors) and a portrait photograph. These should be uploaded and clearly labelled together in a Word document with the revised version of the manuscript. See Information for Authors for further details.

- The reference list must be in alphabetical order, rather than numbered, to comply with our Journal format.

- Your manuscript must include a complete Additional Information section, including competing interests; funding; author contributions and acknowledgements.

- Please upload separate high-quality figure files via the submission form.

- Please ensure that the Article File you upload is a Word file.

- A Data Availability Statement is required for all papers reporting original data. This must be in the Additional Information section of the manuscript itself. It must have the paragraph heading 'Data Availability Statement'. All data supporting the results in the paper must be either: in the paper itself; uploaded as Supporting Information for Online Publication; or archived in an appropriate public repository. The statement needs to describe the availability or the absence of shared data. Authors must include in their statement: a link to the repository they have used, or a statement that it is available as Supporting Information; reference the data in the appropriate sections(s) of their manuscript; and cite the data they have shared in the References section. Whenever possible, the scripts and other artefacts used to generate the analyses presented in the paper should also be publicly archived. If sharing data compromises ethical standards or legal requirements then authors are not expected to share it, but must note this in their statement. For more information, see our Statistics Policy.

- Please include an Abstract Figure file, as well as the Figure Legend text within the main article file. The Abstract Figure is a piece of artwork designed to give readers an immediate understanding of the research and should summarise the main conclusions. If possible, the image should be easily 'readable' from left to right or top to bottom. It should show the physiological relevance of the manuscript so readers can assess the importance and content of its findings. Abstract Figures should not merely recapitulate other figures in the manuscript. Please try to keep the diagram as simple as possible and

without superfluous information that may distract from the main conclusion(s). Abstract Figures must be provided by authors no later than the revised manuscript stage and should be uploaded as a separate file during online submission labelled as File Type 'Abstract Figure'. Please also ensure that you include the figure legend in the main article file. All Abstract Figures should be created using BioRender. Authors should use The Journal's premium BioRender account to export high-resolution images. Details on how to use and access the premium account are included as part of this email.

- Please include a full title page as part of your main article (Word) file, which should contain the following: title, authors, affiliations, corresponding author name and contact details, keywords, and running title.

EDITOR COMMENTS

Reviewing Editor:

The Referees see potential interest in the paper. You should carefully address their comments, preparing a detailed letter, indicating where changes occur and highlighting in color such changes in the text.

Please also see 'Required Items' above.

REFEREE COMMENTS

Referee #1:

This paper proposes a computational framework for electrophysiological (EP) simulations of heterogeneous phenotype-specific hiPSC-CM tissue and the corresponding extracellular potential recorded as a signal. At the cellular level, this framework's novelty lies in using combined ionic models to simulate atrial-like (AL) and ventricular-like (VL) phenotypes, preserving the physiologically diverse electrical responses of AL and VL hiPSC-CMs. At the tissue level, the framework enables the simulation of multi-electrode array (MEA) devices, commonly used in in vitro experiments to measure the EP properties of human cardiomyocytes, specifically hiPSC-CMs. The framework is tested for its ability to predict drug responses in silico, considering four drug compounds and ion channel blockers, with predictions compared against in vitro experimental MEA data. Furthermore, the authors demonstrate the framework's utility in exploring how cellular variations and distributions within human cardiac tissue affect conduction velocities and replicating pathological conditions, such as Brugada syndrome. For the latter, in silico results are compared against experimental measurements.

The work is original in its combination of heterogeneous ionic models to represent AL and VL phenotypes and its investigation of how heterogeneity influences the EP properties of cardiac tissue in both healthy and pathological scenarios. The mathematical model at the tissue level is rigorously defined, and the numerical experiments are well-designed, drawing on thorough literature research and physiological findings.

The experimental data are reliable and provide initial insights into the role of cellular differentiation in EP simulations for understanding physiological mechanisms underlying cardiac pathologies and informing drug testing.

While some limitations exist, the study demonstrates the proposed computational framework's significant potential to influence future research in cardiac EP simulation, drug testing, and response prediction. Additionally, it offers broader insights into the physiological mechanisms underlying cardiac pathologies.

However, the conclusions are valid but not well-structured and, in parts, confusing. Additionally, it places significant emphasis on the mathematical description of the EP models at the tissue level, while the cellular models are only briefly mentioned and cited. Furthermore, the limitations of the proposed study are not adequately addressed. Specifically, my major concerns are:

1. While the introduction adequately discusses the importance of using hiPSC-CMs for experimental design in cardiac experiments and pharmacological research, as well as the utility of MEA devices for recording in vitro measurements, it fails to address existing in silico studies on these topics. Furthermore, the novelty of the proposed methodology is only briefly mentioned in the last paragraph, making it difficult to appreciate the contributions of this work. By contrast, certain sections in the methodology (e.g., pages 7-8) and results (especially Sections 3.2-3.3.4) present extensive literature surveys. I recommend restructuring the introduction to include a well-defined state-of-the-art discussion on in silico modeling of MEA devices and ionic modeling for hiPSC-CMs, followed by a structured description of the proposed approach that highlights its

novelty.

2. I would recommend the authors consider including a final paragraph in the introduction outlining the paper's organization, with a breakdown of sections and subsections. This would improve readability.

3. In Section 2.1, while the Bidomain model is well-known and widely used for both cellular and organ-level simulations, the formulation for extracellular potential recordings via MEA devices is specific to this experimental setup, which resembles a multi-cellular environment rather than an organ-level setting. Typically, at the organ level, the heart is immersed in a bath, and record signals are e.g. ECGs or EGMs using methods like Lead Field, phi-recovery, or Pseudo-Bidomain. The authors should better emphasize that their model for electrode recordings differs from organ-scale approaches.

Furthermore, MEA systems use multiple electrodes, each recording a distinct signal. However, the results section often presents a single signal-are these signals spatially averaged? Lastly, given the familiarity with the Bidomain model, the description and formulation (e.g., initial conditions in Equation 6) could be condensed.

4. In Section 2.2, the authors describe a significant novelty: the use of distinct ionic models for AL and VL cells to reflect phenotype-specific differences. However, much of the description resembles a review of hiPSC-CM modeling literature, which might be better suited to the introduction. Instead, this section could focus on the mathematical formulation of the ionic models used, at least in compact form, given the multiple references to these parameters in the results. Additionally, the paragraph on page 7 discussing ionic models is occasionally unclear and could benefit from revision.

5. The paper states (page 6) that 30% of recorded cells in a standard experiment are AL, per [16], and uses this percentage in simulations. However, subsequent sections, including Figure 2, show diverse percentages. The rationale for changing AL-VL proportions should be explained. Additionally, the authors mention a random selection of AL cells-what random algorithm was used? Could varying distributions affect the results? These questions should be addressed, perhaps in the limitations section.

6. In Section 2.3, the authors note that isotropic tissue is a simplifying assumption. This limitation should also be explicitly stated in the limitations section.

7. In Section 2.4, the authors give a general description of the numerical methods utilized to solve the proposed model, both at the cellular and organ scales. However, these techniques are commonly used in cardiac EP simulations. I would therefore recommend increasing the citation in this section. Moreover, I would suggest better explaining what the different ODE setups are, as mentioned in the last paragraph of this section.

8. Formulas and methods for computing conduction velocities and ionic quantities are introduced in the results section. These would be more appropriate in the methodology.

9. Figure 6: The repolarization time is inherently linked to activation time under the same modeling assumptions; thus, delayed depolarization (e.g., 100% VL cells) also causes delayed repolarization. What happens if repolarization times are normalized to the same initial time? Would late repolarization still be observed in VL-dominant domains? Additionally, I recommend harmonizing Figures 4 and 6 by using the same AL-VL distributions.

10. Page 16, Last Paragraph. The authors describe localized conduction velocity slowing due to Brugada syndrome, yet Figure 10 suggests a more homogeneous decrease. Please clarify and comment on whether this pattern relates to diseased cell distribution.

11. In the drug testing sections, there are often references to the appendix figures. Since the appendix is really brief, I would recommend the authors remove it, and include the figures in the main text of the paper.

12. Page 22 compares the Bidomain and EMI models, implying that the latter is superior except for computational efficiency. Since the computational domain used in this study is relatively small, I would expect a not too high computational costs in general. Could the authors comment on computational time and provide insights into their code's efficiency?

13. On Page 23, the authors mention that extracellular conductivity was reduced to reproduce realistic amplitudes but do not highlight this choice in earlier sections. If this is a key contribution, it should be emphasized in the methodology.

14. To improve readability, I recommend aligning the order of results, discussion, and conclusions. For example, the results sections address conduction velocity first, followed by Brugada syndrome and drug studies, while the discussion and conclusions analyze these in a different order.

Minor Comments:

1. On Page 3, the first sentence of Section 2.1, there probably is a "is" missing after "EP models". I would suggest to rephrase this first sentence.

2. Page 5, under equations 6, "If considering" should be "Concerning"?

3. Page 5, equations 7b, is the domain \partial\Omega_N or _D? According to the paragraph above, that should be a _D, however, these are Neumann conditions.

4. Page 6, after "(APD_20 over APD_90), is there a "was" missing?

5. Caption Fig.3, I would recommend the author to add the explanation for dash and solid lines.

6. Page 8, first row, what datasets for AL have been used?

7. Page 11, paragraph after the formulas, last row. Should the cells here be VL cells instead of AL cells?

8. On Page 15, there is an "in-vitro" that should probably be "in-silico"

9. On Page 15, there is also a "ventricular-like" that should be changed to VL, and the same paragraph seems to be incomplete.

10. Page 16, the third point in the list, there is probably a missed "to". Moreover, in the following paragraph, there is a reference to equation 5 which is probably incorrect.

11. Page 17, toward the end, there is a "blocker blockers", and a missing s in "blocker" in the following row.

12. Fig. 11, in the caption, Fig.11c is described with a red line that should probably be a black line.

Referee #2:

My comments to the authors are in the attached pdf file that has 4 pages.

END OF COMMENTS

Comments for the Author (required)

Botti et al. introduce a comprehensive computational framework that integrates a virtual multi-electrode array (MEA) setup with in silico human induced pluripotent stem cell-derived cardiomyocytes (hiPSC-CMs) models, to predict drug responses. By testing four drug compounds and simulating Brugada syndrome, the authors demonstrate the framework's robustness, while also providing some insights into how tissue heterogeneity could affect electrical behaviour. These findings underscore the potential of this approach to enhance drug screening.

I have five (5) major concerns to be addressed and modified accordingly:

1) The language of individual sentences is clear and readable; however, there are major issues in the structure of the manuscript that make the story hard to follow and rather exhausting to read.

- At least to me, the manuscript appears to include paragraphs and/or text formatted in way that typical for a review paper of a book chapter.
- For example, the Introduction section starts by mentioning regenerative medicine, which is not the topic of the manuscript. Drug testing is the topic, but authors get to only in the 2nd paragraph of the intro. Why not start with that, as the 3rd paragraph describes that very well?
- There's some really generic text in the Intro. For example, this bit: "This work focuses on human induced pluripotent stem cell-derived CMs (hiPSC-CMs) [1, 2]. These cells, derived from reprogrammed somatic cells, can be maintained in a pluripotent state or differentiated into various somatic cell types, including CMs. Notably, hiPSC-CMs bear significant resemblance to adult cardiomyocytes, particularly in their electrophysiological properties, making them valuable in-vitro models for cardiac research." Is that relevant?
- On the other hand, there are also that kind of details in the Intro that could/should actually be in the Methods section: "Our mathematical model builds on the bidomain equations, incorporating an electrode model tailored for MEA devices. The study focuses on a specific device (the 60-6 well MEA by Multi Channel Systems, https://www.multichannelsystems.com), which consists of six independent wells, each containing nine electrodes, allowing for parallel experiments under consistent conditions." And, also this sentence "The data used in this study are sourced from the systems described in [11, 12]." would be better placed in the Methods.
- The word count of the Introduction section is 540. The authors should aim to shorten that with ~100 words. That should be quite straightforward based on the bullet points above.
- There is similar kind of wordiness in other parts of the manuscript as well. For example, the 1st paragraph in the Methods section, which again looks more like something from a book chapter or a review paper.
- Page #17: First six paragraphs of chapter 3.3 should be in the Methods section, as they describe the general approach for modelling the drug-induced current blocks in silico.

- Page #19, #20 and #21: Last sentence of chapter 3.3.1, last two sentences of chapter 3.3.2, last two sentences of chapter 3.3.3, and last three sentences of chapter 3.3.4 read like points that should be made in the Discussion section instead of Results.
- Overall, the manuscript does not appear to be formatted according to the J Physiol guidelines. Starting from the abstract that is supposed to written as a continuous, unstructured paragraph. The Figure legends have also some unusual formatting. For example, in Figure 1 referring to the figure panels could be done briefly as a) and b), instead of Fig. 1a) and Fig. 1b).
- These observations apply to the whole manuscript. Thus, the authors should reformat and edit the text so that it complies the J Physiol guidelines.

2) About the Figures:

- Figure 3 is not very informative in its current format. Please add panels that show traces for those ion currents that underlie the differences between the atrial-like and ventricular-like virtual cells.
- Extend Figure 8 with additional panels to include 1) the simulated "Brugada AP" from the Botti24 model, and 2) the traces of those ion currents (both control and "Brugada" cases), whose conductances were modified to obtain the "Brugada" variant of the CM models.
- Presenting part of the main results in the Appendix makes the story difficult to follow. And, I do not think that is in accordance with J Physiol guidelines either. Please, integrate the drug-specific panels of Figure A.15 with corresponding Figures 11-14.

3) The authors need to critically re-evaluate their claims regarding 1) their framework's utility and 1) physiological significance of the simulation results, in the context of the VL vs. AL hiPSC-CM phenotype. This applies, for example, to pages #14 and #26. The fundamental problem with comparing the VL and AL model variants, which are used in this study, is that they are <u>not based on coherent in vitro data sets</u>. It is like "comparing apples and oranges", as the common idiom states. To my knowledge, there are no published in vitro studies that would show, for example, that the minimum diastolic potential (MDP) would be more negative in AL vs. VL hiPSC-CMs or engineered heart tissue (EHT) in coherent data sets. Please correct me, if I am wrong. The only coherent datasets that I am aware of, indicate the opposite. Check out Figure 6A in Lemme et al. (2018; https://doi.org/10.1016/j.stemcr.2018.10.008) regarding the MDP in AL vs. VL EHT. Those findings are in line with I_K1 amplitudes – measured in coherent in vitro conditions – that are about 7 vs. 14 pA/pF at -100 mV as reported by Schulz et al. (2023; https://doi.org/10.1161/circep.122.011602) and Horvath et al. (2018; https://doi.org/10.1016/j.stemcr.2018.01.012) for AL vs. VL hiPSC-CMs, respectively. The results that authors present are valid and insightful in the context of tissue heterogeneity but not in the context AL vs. VL hiPSC-CMs phenotype.

4) About the modelling of the Brugada Syndrome (BrS). While the authors have cleverly selected BrS as a disease case to investigate with their frameworks, they are hampering potential significance of their findings by selecting a generic, "top-down" way to implement the BrS variants of the in silico cell models. There are plenty of hiPSC-based in vitro studies that report the changes in I_Na properties. For example, a search "brugada hiPSC patch-clamp" gives 18 results in PubMed. I would urge the authors to select a specific BrS-causing mutation and to implement a corresponding "bottom-up" BrS variants of the cell models. The simulations results obtained with that approach are more physiologically relevant and

potentially much more insightful. That is because – at least to my knowledge – the weaker I_Na in BrS is commonly a combination of decreased sodium channel expression and shifted voltage dependencies of activation & inactivation (and in some mutations, also recovery kinetics) of I_Na. Those alterations together with the tissue heterogeneity, which authors can account for in their framework, would potentially result in much more complex changes in electrical function of the virtual tissue.

5) On page #18, the authors state: "Conversely, by reducing late-phase calcium entry, mexitiline…". However, Figure A15 does not indicate that. In the panel 2nd from the top on the left (Paci20 model), the amplitude of I_CaL appears to be larger during the late phase of the action potential. In the same Figure, corresponding panel on the right, it is not possible to compare the traces of control vs. mexitiline for the Botti24 model, because the figure panel appears to include some other current than I_CaL (perhaps I_Na?). The 5th panel from the top on the right (i.e., the "nifedipine" panel) in that same Figure appears to include actual I_CaL traces. And they do not correspond with the panel 2nd from the top on the right. Please correct the Figure. Also, consider including an additional panel that zooms in the differences of the I_CaL and/or calculating the integral of I_CaL. That is, if the authors make some statements in the text about calcium entry changing, that the difference should be quantified and/or clearly visible in the Figure.

Minor points to consider and fix:

- Page #2: What is this comma before the citation? That is not standard formatting, right? For example: "discovery, [4]."
- Page #2: "insights into long-term maturation"; reference?
- Page #2: "due to variability"; variability of what exactly?
- Figure 2: "spontaneously beating" instead of "spontaneous beating"
- Page #6: "...AP triangular shape"; reference?
- Page #6: I think proper English phrase is "According to" or "In accordance with" instead of "According with"?
- Page #7: Very long sentence that is difficult to follow. Please, rewrite as two separate sentences: "In Paci13 [20], the authors introduce AL and VL ionic models, highlighting that the primary distinctions lie in the conductances of certain channels and the dynamics of ICaL, which primarily contributes to the plateau phase; but it was based on recordings obtained from a mixture of nodal- ventricular and atrial–like hiPSC–CMs and the reproduce the phenotypical heterogeneity different scalings were considered, instead of phenotype–specific currents."
- Page #7: Please write "real-time" instead of "real time"?
- Figure 3: Please write "spontaneous" instead of "unpaced", for the sake of consistency.
- Page #8: Should be "immature" instead of "mature"?
- Page #8: Please write "Thus, here" instead of "Thus"?
- Page #9: Please write "which results in" instead of "we reach the"?
- Page #9: Please write "4 cm/s matching [11]." instead of "4 cm/s provided by the study of [11]."?
- Page #9: Please write "conduction" instead of "conduction velocity"?
- Page #13: The cv definition should be in Methods?
- Page #13, this appears borderline trivial: "Furthermore, an analysis of the Repolarization Times reveals that AL tissues tend to repolarize more quickly,

requiring approximately 100 ms less with respect to the homogeneous VL tissue to return to the resting potential. This finding aligns with the triangular shape of the AL AP, which characterizes their faster recovery dynamics compared to other tissue types." I mean, what else could one expect, as AL cells have shorter APs than VL cells. So, maybe rephrase this to something like: "As expected based on the shorter APs in AL cells, …"?

- Page #15: Please write "hiPSC-CM-based in vitro models" instead of "to in-vitro models proposed in this study, based on hiPSC-CMs"?
- Page #15: Something important missing here: "protocol, which leads to the 30"?
- Figure 8: What does "ionic AP" mean, in contrast to just an "AP"? Please write simply "AP" instead.
- Page #16: Something missing here: "and set to 110"?
- Page #17: Please write "blockers" instead of "blocker"?
- Page #22: Please consider rewriting the 3rd paragraph of Discussion, with a bit less repetition regarding the EMI model.
- Page #22: Please write "VL cells" and "AL cells" instead of "VL stem cells" and "AL stem cells". After the CMs have been derived and differentiated from stem cells, they are no longer stem cells, right?
- Page #24: It is not clear, how the tool will potentially benefit "regenerative medicine". So, maybe it is more reasonable to make statements on the pharmacological aspects only?

February 16, 2025

Dear Editors of The Journal of Physiology,

Enclosed please find a copy of the revised manuscript “*In-silico* Modeling of Multi-Electrode Arrays for Enhancing Cardiac Drug Testing on hiPSC-CMs Heterogeneous Tissues”, coauthored by Sofia Botti, Luca F. Pavarino and Rolf Krause, which we resubmit for publication in The Journal of Physiology.

We have taken into account the changes requested by the Referees, that we would like to thank for their valuable comments that have helped us to improve the manuscript. Our detailed answers and list of changes to the specific comments raised by the Referees follow below. The revised text in the manuscript is marked in red color.

We look forward to hearing from you soon. Thank you and best regards. Sincerely,

Sofia Botti
Luca F. Pavarino
Rolf Krause

Referee Report (# 1)

This paper proposes a computational framework for electrophysiological (EP) simulations of heterogeneous phenotype-specific hiPSC-CM tissue and the corresponding extracellular potential recorded as a signal. At the cellular level, this framework's novelty lies in using combined ionic models to simulate atrial- like (AL) and ventricular-like (VL) phenotypes, preserving the physiologically diverse electrical responses of AL and VL hiPSC-CMs. At the tissue level, the framework enables the simulation of multi-electrode array (MEA) devices, commonly used in in vitro experiments to measure the EP properties of human cardiomyocytes, specifically hiPSC-CMs. The framework is tested for its ability to predict drug responses in silico, considering four drug compounds and ion channel blockers, with predictions compared against in vitro experimental MEA data. Furthermore, the authors demonstrate the framework's utility in exploring how cellular variations and distributions within human cardiac tissue affect conduction velocities and replicating pathological conditions, such as Brugada syndrome. For the latter, in silico results are compared against experimental measurements.

The work is original in its combination of heterogeneous ionic models to represent AL and VL phenotypes and its investigation of how heterogeneity influences the EP properties of cardiac tissue in both healthy and pathological scenarios. The mathematical model at the tissue level is rigorously defined, and the numerical experiments are well-designed, drawing on thorough literature research and physiological findings.

The experimental data are reliable and provide initial insights into the role of cellular differentiation in EP simulations for understanding physiological mechanisms underlying cardiac pathologies and informing drug testing.

While some limitations exist, the study demonstrates the proposed computational framework's significant potential to influence future research in cardiac EP simulation, drug testing, and response prediction. Additionally, it offers broader insights into the physiological mechanisms underlying cardiac pathologies.

However, the conclusions are valid but not well-structured and, in parts, confusing. Additionally, it places significant emphasis on the mathematical description of the EP models at the tissue level, while the cellular models are only briefly mentioned and cited. Furthermore, the limitations of the proposed study are not adequately addressed. Specifically, my major concerns are:

1. While the introduction adequately discusses the importance of using hiPSC-CMs for experimental design in cardiac experiments and pharmacological research, as well as the utility of MEA devices for recording in

vitro measurements, it fails to address existing in silico studies on these topics. Furthermore, the novelty of the proposed methodology is only briefly mentioned in the last paragraph, making it difficult to appreciate the contributions of this work. By contrast, certain sections in the methodology (e.g., pages 7-8) and results (especially Sections 3.2-3.3.4) present extensive literature surveys. I recommend restructuring the introduction to include a well-defined state-of-the-art discussion on in silico modeling of MEA devices and ionic modeling for hiPSC-CMs, followed by a structured description of the proposed approach that highlights its novelty.

Answer. Thank you for raising these important points. We have restructured the Intro- duction and included a comprehensive state-of-the-art discussion on *in-silico* modeling of MEA devices and ionic modeling for hiPSC-CMs. To achieve this, we moved the relevant literature previously presented in the Methods section to the Introduction, ensuring a clearer and more coherent narrative. We also moved the description of ionic models to the Discussion to provide readers with the necessary context and to highlight the novelty of our approach. However, we respectfully retained the detailed discussion of the Brugada syndrome-specific model in the Methodology section, as it is highly specialized and too extensive to be effectively summarized in the Introduction. This choice is also due to the request of Referee 2 to reduce considerably the Introduction's length. We believe that this organization best balances readability while preserving scientific depth. We appreciate your constructive feedback, which has greatly improved the clarity and structure of our work.

2. I would recommend the authors consider including a final paragraph in the introduction outlining the paper's organization, with a breakdown of sections and subsections. This would improve readability.

Answer. We have added a final paragraph in the Introduction outlining the paper's organization.

3. In Section 2.1, while the Bidomain model is well-known and widely used for both cellular and organ-level simulations, the formulation for extracellular potential recordings via MEA devices is specific to this experimental setup, which resembles a multi-cellular environment rather than an organ-level setting. Typically, at the organ level, the heart is immersed in a bath, and record signals are e.g. ECGs or EGMs using methods like Lead Field, phi- recovery, or Pseudo-Bidomain. The authors should better emphasize that their model for electrode recordings differs from organ-scale approaches.

Furthermore, MEA systems use multiple electrodes, each recording a distinct signal. However, the results section often presents a single signal-are these signals spatially averaged? Lastly, given the familiarity with the Bidomain model, the description and formulation (e.g., initial conditions in Equation 6) could be condensed.

Answer. Thank you for this clarification. We have added on p. 5 that "We remark that this MEA Bidomain model of electrode recordings differs from organ-level Bidomain settings where electrode signals are simulated with pseudo-ECG, Lead Field, and similar approaches.".

We have added panel (a) in Fig. 8 showing the MEA setup and the position of the 9 electrodes e1 - e9 in our simulations. In the same figure, we also added two other panels (b) and (d) showing respectively the action potentials and field potentials recorded at the 9 electrodes. In all other figures in the paper, we show only the recording at the central electrode e5 for better clarity.

We have condensed the initial conditions (6) on a single line and also the boundary conditions in three more compact items (i), (ii), (iii) on p. 6.

4. In Section 2.2, the authors describe a significant novelty: the use of distinct ionic models for AL and VL cells to reflect phenotype-specific differences. However, much of the description resembles a review of hiPSC-CM modeling literature, which might be better suited to the introduction. Instead, this section could focus on the mathematical formulation of the ionic models used, at least in compact form, given the multiple references to these parameters in the results. Additionally, the paragraph on page 7 discussing ionic models is occasionally unclear and could benefit from revision.

Answer. We appreciate the referee's suggestion regarding Section 2.2. Following this recommendation, we have moved the review of hiPSC-CM modeling literature to the Discussion (since the Introduction had to be shortened due to a specific request of Referee 2), as we also consider ionic models that have not been used in our specific simulations. To improve clarity and conciseness, we have added a summary of the main ionic currents for each model in Section 2.2. However, given the complexity and length of the mathematical formulations, we refer the reader to the supplementary material of the original papers for full details. Additionally, we have revised the discussion of ionic models on page 7 to enhance clarity and coherence. We hope this reformulation better aligns with the referee's expectations. Thank you for the valuable feedback.

5. The paper states (page 6) that 30% of recorded cells in a standard experiment are AL, per [16], and uses this percentage in simulations. However, subsequent sections, including Figure 2, show diverse percentages. The rationale for changing AL-VL proportions should be explained. Additionally, the authors mention a random selection of AL cells - what random algorithm was used? Could varying distributions affect the results? These questions should be addressed, perhaps in the limitations section.

Answer. Thank you for allowing us to clarify this point.

We have added on p. 21 (sec. 3.5) that "we focus on the implications of drug interactions specifically for the VL and AL hiPSC-CMs. Since our interest is focused on the effect of the drug, a standard differentiation protocol is assumed in our simulations, while the effects of different heterogeneity degrees are excluded. Following the description of the

standard differentiation protocol analyzed in Altomare (2023), we set the 30% of AL hiPSC-CMs in the simulated culture."
Moreover, we have added a Limitations subsection at the end of the Discussion, where we have stated that we have considered only a uniform random distribution of AL and VL cells, and that other distributions could be investigated as well.

6. In Section 2.3, the authors note that isotropic tissue is a simplifying assumption. This limitation should also be explicitly stated in the limitations section.

Answer. We have added in the Limitations subsection that we have considered an isotropic tissue since no preferential conduction direction is shown in MEA recordings.

7. In Section 2.4, the authors give a general description of the numerical methods used to solve the proposed model, both at the cellular and organ scales. However, these techniques are commonly used in cardiac EP simulations. I would therefore recommend increasing the citation in this section. Moreover, I would suggest better explaining what the different ODE setups are, as mentioned in the last paragraph of this section.

Answer. We sincerely thank the referee for the insightful comments on Section 2.4. In response to the suggestion to increase the citations, we have thoroughly revised the section to include additional references that highlight the numerical techniques commonly adopted in cardiac electrophysiology simulations. This provides a more comprehensive context for the methods employed in our study.
Regarding the request for clarification of the different ODE setups, we have added detailed explanations to Section 2.4. Specifically, we clarified that in the various setups presented in the Results section, the simulations for updating the initial conditions of the ionic models were conducted by solving systems of ODEs using the Matlab built-in solver ode15s, which is designed for stiff systems. The solver was configured with an initial step size of 2×10^{-5}, s and a maximum allowable step size of 1, ms. Additionally, we performed numerical tests by varying the relative and absolute tolerance parameters of ode15s, setting RelTol = 10^{-3} and AbsTol = 10^{-6}. These parameter choices were carefully selected to ensure accurate reproduction of the action potential morphology. We trust that these revisions address the referee's concerns and enhance the clarity and scientific rigor of Section 2.4.

8. Formulas and methods for computing conduction velocities and ionic quantities are introduced in the results section. These would be more appropriate in the methodology.

Answer. Thank you for correcting this point. We moved the formulas and methods for computing the conduction velocity to the Methods, subsection 2.6. We also moved to this section the definition of Activation Time AT, Repolarization Time RT, APD (subsection 2.5), as well as the formulas for modelling the Brugada Syndrome effects (subsection 2.7) and the drug effects on the ionic currents (subsection 2.8).

9. Figure 6: The repolarization time is inherently linked to activation time un- der the same modeling assumptions; thus, delayed depolarization (e.g., 100% VL cells) also causes delayed repolarization. What happens if repolarization times are normalized to the same initial time? Would late repolarization still be observed in VL-dominant domains? Additionally, I recommend harmonizing Figures 4 and 6 by using the same AL-VL distributions.

Answer. We appreciate the referee's insightful suggestion and fully understand the im- portance of normalizing repolarization times to ensure a coherent and consistent analysis. In response, we have added a detailed section (3.3) on the action potential duration (APD) to provide a suitable normalization framework. This addition allows for a more comprehensive evaluation of the interplay between activation and repolarization times. Moreover, as recommended, we have harmonized Figures 4, 6 (and also 5, 7) by adopting the same AL-VL distributions, enhancing their comparability. The updated Figure 6 now includes six panels, which display activation time, depolarization time, and APD for both AL and VL-dominant domains. The results clearly demonstrate that even when repolarization times are normalized to the same initial activation time, the late repolarization trends in VL-dominant domains persist, highlighting the robustness of this phenomenon.

10. Page 16, Last Paragraph. The authors describe localized conduction velocity slowing due to Brugada syndrome, yet Figure 10 suggests a more homogeneous decrease. Please clarify and comment on whether this pattern relates to diseased cell distribution.

Answer. Thank you for pointing out this inconsistency. The homogeneous decrease in the old Fig. 10 was due to a wrong colorbar range. We have redrawn the figure using a proper range in the colorbar and in the new figure (now Fig. 11) the conduction velocity appears to be reduced and heterogeneous as described in the text. We report below the old and new Figures.

Old Fig. 10:

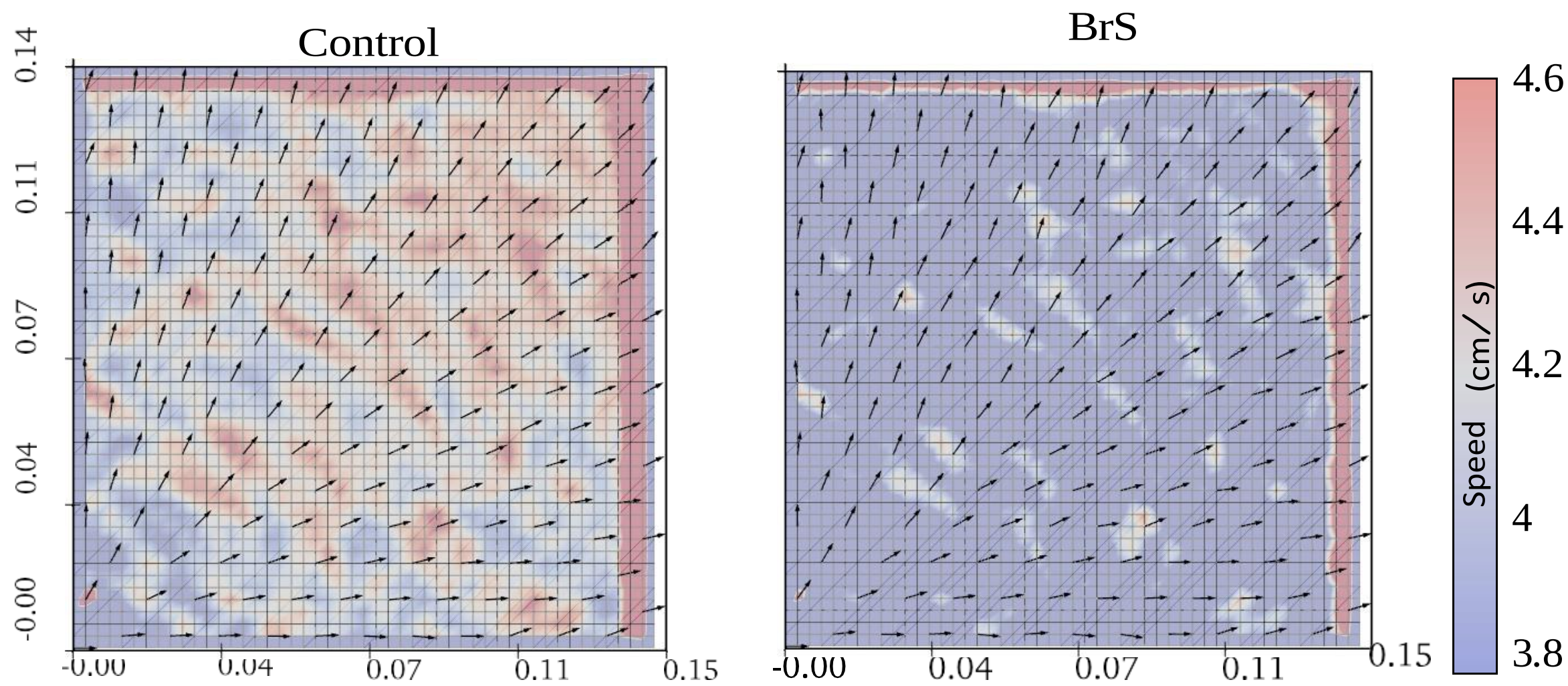

New Fig. 11:

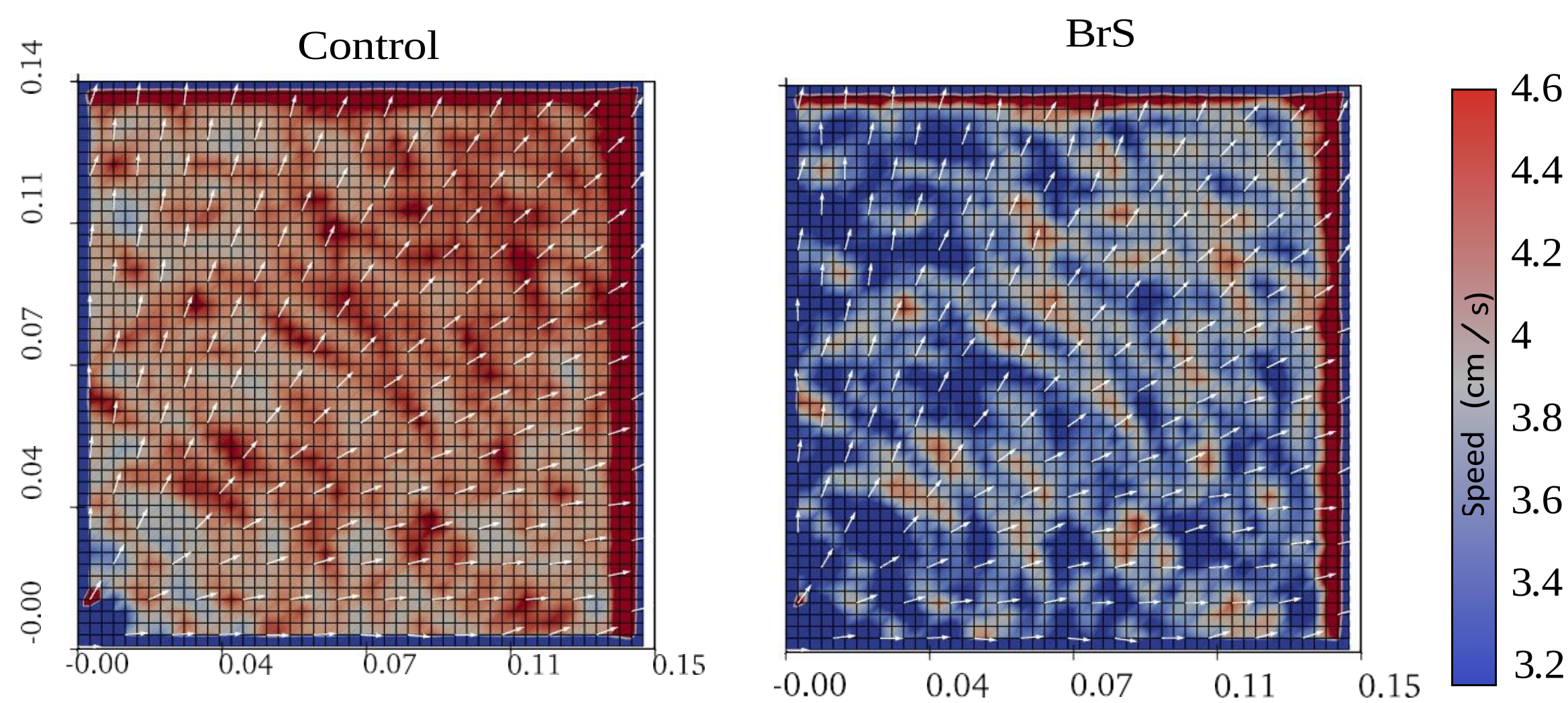

11. In the drug testing sections, there are often references to the appendix figures. Since the appendix is really brief, I would recommend the authors remove it, and include the figures in the main text of the paper.

Answer. Thank you for this valuable recommendation. We have removed the Appendix and moved all its material to the main body of the paper, in particular in the drug testing section (3.5) of the Results.

12. Page 22 compares the Bidomain and EMI models, implying that the latter is superior except for computational efficiency. Since the computational domain used in this study is relatively small, I would expect a not too high

computational costs in general. Could the authors comment on computational time and provide insights into their code's efficiency?

Answer. We thank the referee for this valuable comment. As requested, we have provided additional details on the computational efficiency of our code in the manuscript. Specifically, we have clarified in subsection 3.4 of the Methods that our simulations were performed on a single CPU with 32 threads, requiring approximately 3 hours for the full computational domain. We also included at the end of the Discussion some comments on the potential computational costs associated with the EMI model and highlighted future optimization strategies to enhance the performance of the bidomain simulations. We believe these changes address the referee's concerns and improve the clarity of the manuscript.

13. On Page 23, the authors mention that extracellular conductivity was reduced to reproduce realistic amplitudes but do not highlight this choice in earlier sections. If this is a key contribution, it should be emphasized in the methodology.

Answer. As recommended, we have added to subsection 2.3 on Tissue conductivity a sentence specifying that: "we choose $\sigma^i = 0.01$ mS/cm and $\sigma^e = 0.1$ mS/cm, as suggested in Jæger et al. (2021a), and which results in a conduction velocity equal to about 4 cm/s, matching the values in Abbate et al. (2018). These values are lower than those observed in human cardiac tissue, as the conduction in hiPSC-CMs is significantly slower compared to that in the human heart."

14. To improve readability, I recommend aligning the order of results, discus- sion, and conclusions. For example, the results sections address conduction velocity first, followed by Brugada syndrome and drug studies, while the discussion and conclusions analyze these in a different order.

Answer. We have restructured the Discussion section in order to align it with the order used in the Results section. We have also added a few subheadings in bold face in order to clarify the organization of the Discussion section.

Minor Comments:

1. On Page 3, the first sentence of Section 2.1, there probably is a "is" missing after "EP models". I would suggest to rephrase this first sentence.

Answer. We rephrased the first sentence as: "In cardiac electrophysiology modeling, the electrical behaviour of cardiac tissue in space ($\boldsymbol{x} \in \Omega$) and time ($t \in (0, T)$) is described by means of two superimposed continua, the intra- and the extra- cellular spaces."

2. Page 5, under equations 6, "If considering" should be "Concerning"?

Answer. Thank you, we have corrected the sentence and streamlined the description of the boundary conditions of the EMI system.

3. Page 5, equations 7b, is the domain $\partial\Omega\ N$ or $\_D$? According to the para- graph above, that should be a D, however, these are Neumann conditions.

Answer. Thank you for spotting this mistake. We have corrected the boundary conditions description as follows:

"We also impose the following boundary conditions, where **n** is the outward pointing normal:

(i) homogeneous Neumann boundary conditions associated with the no flux assumption of the intracellular potential on the external surface $\partial\Omega$: $D_i\nabla(v + u_e) \cdot \mathbf{n} = 0$ on $\partial\Omega \times (0, T)$;

(ii) homogeneous Dirichlet boundary conditions on the three edges connected to the ground: $D_e\nabla u_e \cdot \mathbf{n} = 0$ on $\partial\Omega_N \times (0, T)$;

(iii) Neumann non-flux boundary condition for the remaining edge: $u_e = 0$ on $\partial\Omega_D \times (0, T)$. "

4. Page 6, after "(APD_{20} over APD_{90}), is there a "was" missing?

Answer. Yes, thank you. We have corrected the typo.

5. Caption Fig.3, I would recommend the author to add the explanation for dash and solid lines.

Answer. Thank you for the suggestion. The figure has been changed extensively according to the suggestions of referee 2. The current version also includes an explanation of dash and solid lines.

6. Page 8, first row, what datasets for AL have been used?

Answer. We added the following reference of the used dataset in the manuscript:

"AL datasets presented in Altomare et al. (2023), recorded under DC, have been recently used to build a novel atrial-specific ionic model, Botti2024 (2024)."

7. Page 11, paragraph after the formulas, last row. Should the cells here be VL cells instead of AL cells?

Answer. Yes, thank you, we have corrected the mistake in the manuscript, changing the sentence to: "These plots show that tissues with AL cells exhibits a higher conduction velocity when compared to tissues with VL hiPSC-CMs cells."

8. On Page 15, there is an "in-vitro" that should probably be "in-silico"

Answer. Yes, we have corrected the text as: "However, these insights may not fully apply to hiPSC-CM-based *in-silico* models, where the heterogeneous differentiation of the cells makes it challenging to map specific substrates to anatomical regions, such as the right ventricle."

9. On Page 15, there is also a "ventricular-like" that should be changed to VL, and the same paragraph seems to be incomplete.

Answer. Thank you for noticing this omission. We have restored the final part of the sentence as follows: "Following the approach proposed by Hoogendijk et al. (2011), where ST-segment elevation in BrS is linked to reduced sodium channel (g_{Na}) and L- type calcium channel (g_{CaL}) conductances alongside increased potassium channel (g_{to}) conductance, we simulate these ionic alterations using the Paci2020 VL hiPSC-CM model. In our approach, we adjust g_{Na}, g_{CaL}, and g_{to} under the assumption that all cells within the simulated tissue region exhibit characteristics of BrS. Furthermore, since our interest is focused on a ventricular-specific disorder, we consider the standard differentiation protocol, which leads to the 30 % of VL hiPSC-CMs in the culture."

10. Page 16, the third point in the list, there is probably a missed "to". Moreover, in the following paragraph, there is a reference to equation 5 which is probably incorrect.

Answer. We have corrected both the typo and the reference. The corrected sentence is now: "g_{to} is increased and set to 110 % of its normal value. This observation is quantitatively confirmed by calculating the average conduction velocity using Eq. (20), which yields a value of 3.59 cm/s in the tissue affected by BrS."

11. Page 17, toward the end, there is a "blocker blockers", and a missing s in "blocker" in the following row.

Answer. Thank you, we have eliminated both typos and restructured several paragraphs of the Results section on current blockers' effects.

12. Fig. 11, in the caption, Fig.11c is described with a red line that should probably be a black line.

Answer. Thank you for noticing, there was a mistake in the figure generation which now has been corrected. In addition, the figure has been changed extensively by taking into account the valuable suggestion of removing the appendix and redistributing its material to the Results section.

Referee Report (# 2)

Answer. We sincerely appreciate the referee's valuable comments, which greatly improve the manuscript's readability and clarify the presentation of our results. Below, we provide a detailed, point-by-point response.

Botti et al. introduce a comprehensive computational framework that integrates a virtual multi-electrode array (MEA) setup with in silico human induced pluripotent stem cell-derived cardiomyocytes (hiPSC-CMs) models, to predict drug responses. By testing four drug compounds and simulating Brugada syndrome, the authors demonstrate the framework's robustness, while also providing some insights into how tissue heterogeneity could affect electrical behaviour. These findings underscore the potential of this approach to enhance drug screening.

I have five (5) major concerns to be addressed and modified accordingly:

1. The language of individual sentences is clear and readable; however, there are major issues in the structure of the manuscript that make the story hard to follow and rather exhausting to read.

- **At least to me, the manuscript appears to include paragraphs and/or text formatted in way that typical for a review paper of a book chapter.**

Answer. The manuscript has changed considerably thanks to both referees' suggestions and advices. The paragraphs previously identified as resembling a review paper have been removed or adjusted due to the changes we made.

- **For example, the Introduction section starts by mentioning regenerative medicine, which is not the topic of the manuscript. Drug testing is the topic, but authors get to only in the 2nd paragraph of the intro. Why not start with that, as the 3rd paragraph describes that very well?**

Answer. As requested, we have restructured the Introduction to start directly with drug testing instead of regenerative medicine. This change aligns the content with the primary focus of the manuscript and makes the narrative more coherent and impactful.

- **There's some really generic text in the Intro. For example, this bit: "This work focuses on human induced pluripotent stem cell-derived CMs (hiPSC- CMs) [1, 2]. These cells, derived from reprogrammed somatic cells, can be maintained in a pluripotent state or differentiated into various somatic cell types, including CMs. Notably, hiPSC-CMs bear significant resemblance to adult cardiomyocytes, particularly in their electrophysiological properties, making them valuable in-vitro models for cardiac research." Is that relevant?**

Answer. We have removed overly generic text and ensured that the content focuses only on aspects directly relevant to drug testing. We minimized general descriptions about hiPSC-CMs, emphasizing instead their electrophysiological properties and importance in pharmacological research.

- **On the other hand, there are also that kind of details in the Intro that could/should actually be in the Methods section: "Our mathematical model builds on the bidomain equations, incorporating an electrode model tailored for MEA devices. The study focuses on a specific device (the 60-6 well MEA by Multi Channel Systems, https://www.multichannelsystems.com), which consists of six independent wells, each containing nine electrodes, allowing for parallel experiments under consistent conditions." And, also this sentence "The data used in this study are sourced from the systems described in [11, 12]." would be better placed in the Methods.**

Answer. Thank you for this helpful suggestions. We have moved the MEA Multi Channel sentences to the Method section (subsection 2.1), while we removed the second sentence.

- **The word count of the Introduction section is 540. The authors should aim to shorten that with 100 words. That should be quite straightforward based on the bullet points above.**

Answer. As requested, we have significantly shortened the Introduction by removing some review sentences, relocating others to the Discussion, and condensing the remaining text. Additionally, we have carefully revised the Introduction to align with The Journal of Physiology guidelines, which state: "The Introduction should present the context and rationale of the study and introduce what was known and unknown on the topic."

- **There is similar kind of wordiness in other parts of the manuscript as well. For example, the 1st paragraph in the Methods section, which again looks more like something from a book chapter or a review paper.**

Answer. We have compacted several parts of the Methods section. We retained a compacted version of Subsection 2.1 describing the mathematical model employed in the paper, since this coupling of MEA Bidomain and hiPSC-CM ionic models is novel.

- **Page #17: First six paragraphs of chapter 3.3 should be in the Methods section, as they describe the general approach for modelling the drug-induced current blocks in silico.**

Answer. As requested, we have moved the description of drug modeling from Chapter 3.3 to the Methods, subsection 2.8.

- **Page #19, #20 and #21: Last sentence of chapter 3.3.1, last two sentences of chapter 3.3.2, last two sentences of chapter 3.3.3, and last three sentences of chapter 3.3.4 read like points that should be made in the Discussion section instead of Results.**

Answer. We acknowledge that some parts of the Discussion were mixed with the Results. As suggested, we have relocated the parts indicated above to the Discussion section, improving the discussion of the compounds' effects and their clinical perspectives in the appropriate section (pages 31 - 32).

- **Overall, the manuscript does not appear to be formatted according to the J Physiol guidelines. Starting from the abstract that is supposed to written as a continuous, unstructured paragraph. The Figure legends have also some unusual formatting. For example, in Figure 1 referring to the figure panels could be done briefly as a) and b), instead of Fig. 1a) and Fig. 1b).**

Answer. Thank you for pointing out the abstract and figure legend formatting issues. We revised the abstract to follow a continuous paragraph structure and adjusted the figure legends according to The Journal of Physiology guidelines.

- **These observations apply to the whole manuscript. Thus, the authors should reformat and edit the text so that it complies the J Physiol guidelines.**

Answer. We carefully reviewed the entire manuscript to ensure that it complies with The Journal of Physiology formatting requirements. We hope all elements are now correctly aligned with the guidelines. Thank you once again for your valuable feedback, which has helped improve the clarity and presentation of the manuscript.

2) About the Figures:

2.1) Figure 3 is not very informative in its current format. Please add panels that show traces for those ion currents that underlie the differences between the atrial-like and ventricular-like virtual cells.

Answer. We sincerely thank the referee for these constructive suggestions. We agree that the previous format of Fig. 3 was less informative. As requested, we have added additional panels to the revised Fig. 3, reported below, showing ion current traces that highlight the differences between atrial-like and ventricular-like cells. This enhancement improves clarity and strengthens the figure's interpretability.

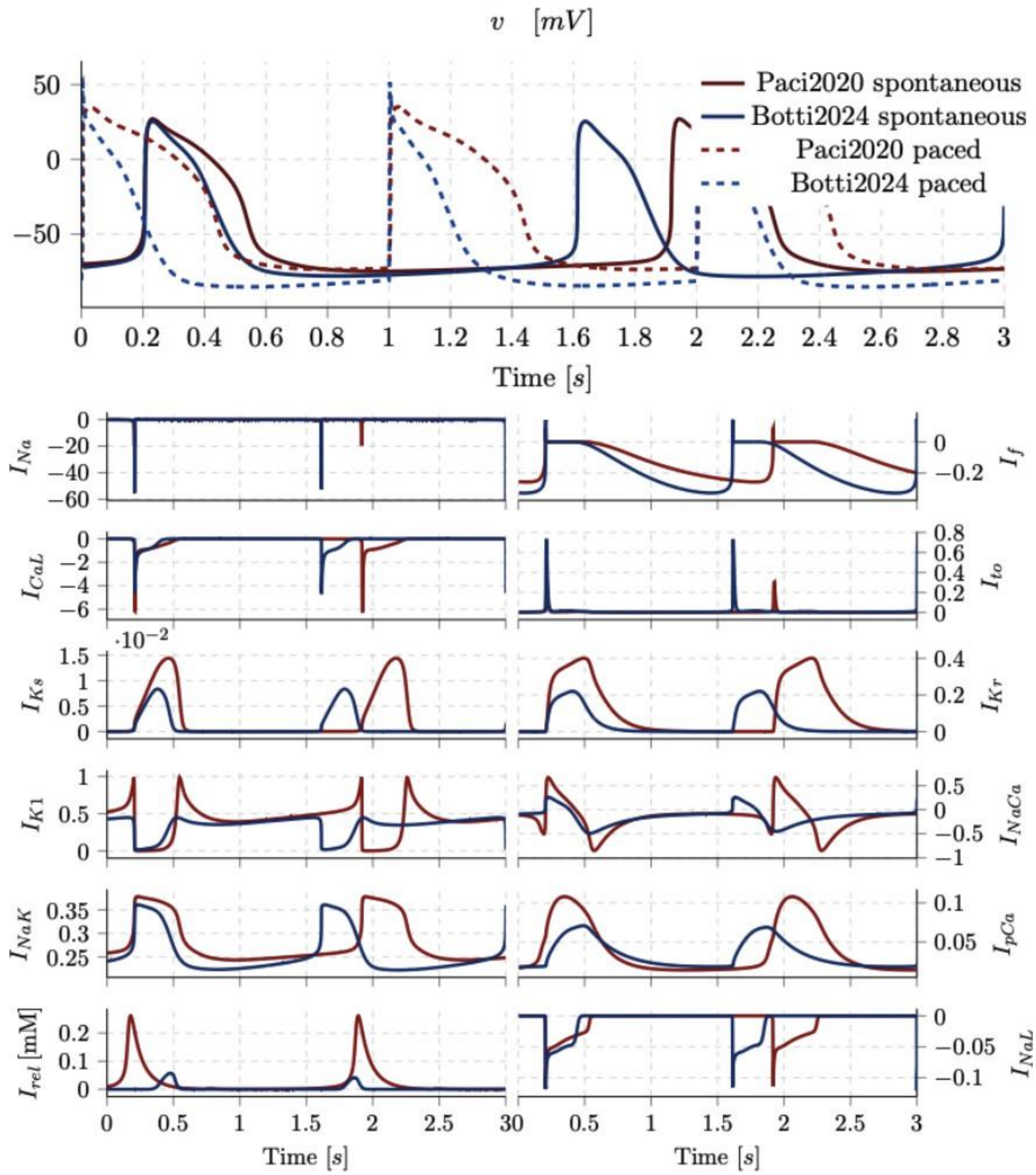

2.2) Extend Figure 8 with additional panels to include 1) the simulated "Brugada AP" from the Botti24 model, and 2) the traces of those ion currents (both control and "Brugada" cases), whose conductances were modified to obtain the "Brugada" variant of the CM models.

Answer. Thank you for this insightful suggestion. We have included in the revised figure (now Fig. 9, reported below) the traces of the ion currents (both control and Brugada variants) whose conductances were modified to generate the Brugada variant of the CM models. However, we did not include changes in the Botti24 model because it was not affected by any of these modifications. Brugada syndrome is a ventricular-specific condition, while the

Botti24 model was developed for atrial-like cells.

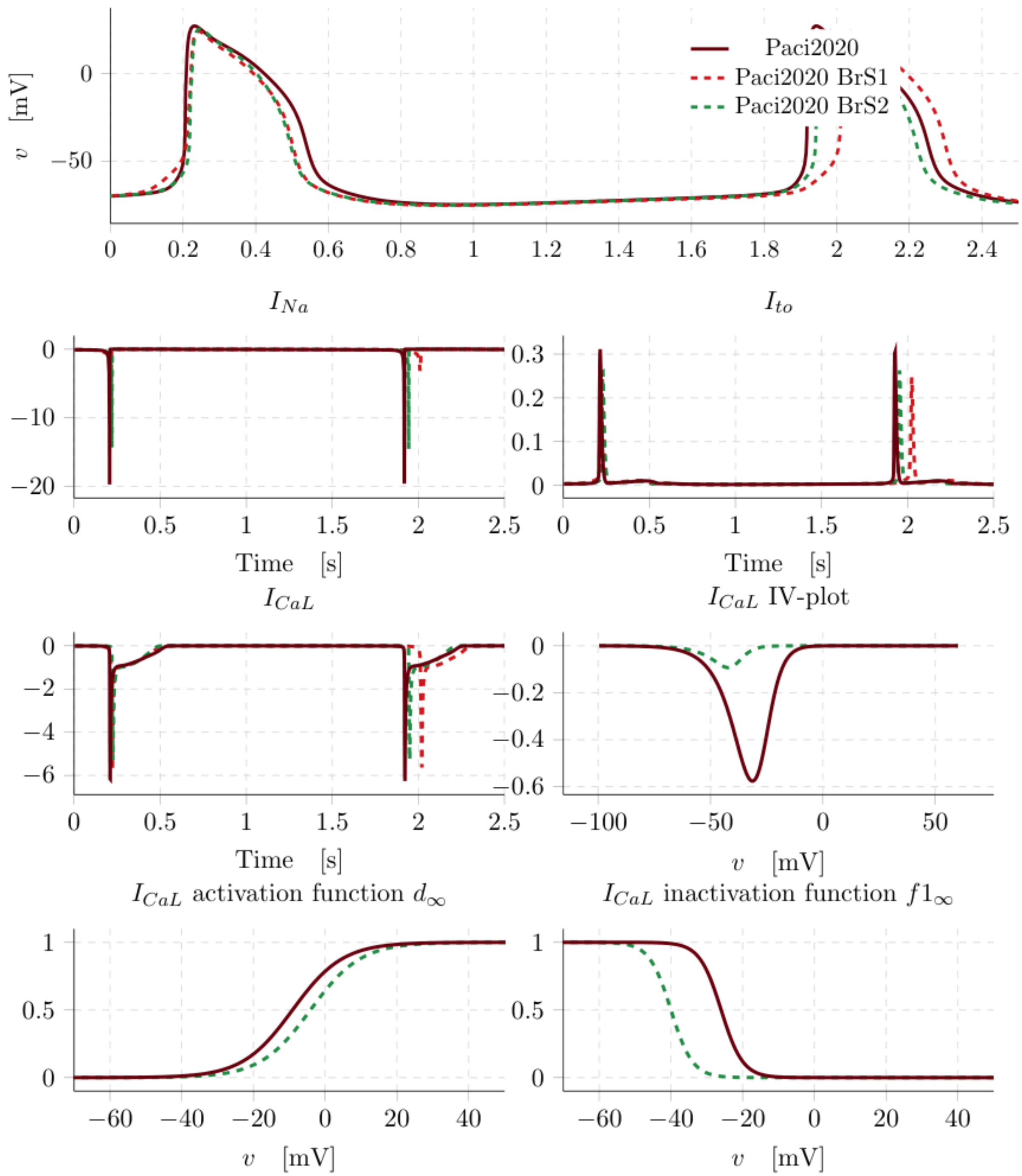

2.3) Presenting part of the main results in the Appendix makes the story difficult to follow. And, I do not think that is in accordance with J Physiol guidelines either. Please, integrate the drug-specific panels of Figure A.15 with corresponding Figures 11-14.

Answer. Thank you for pointing out the potential confusion and alignment issue with the journal guidelines. We have fully integrated the drug-specific panels of Figure A.15 with corresponding Figures 11-14. This restructuring improves the manuscript flow and aligns better with the journal's presentation standards.

3) The authors need to critically re-evaluate their claims regarding 1) their framework's utility and 1) physiological significance of the simulation results, in the context of the VL vs. AL hiPSC-CM phenotype. This applies, for example, to pages #14 and #26. The fundamental problem with comparing the VL and AL model variants, which are used in this study, is that they are not based on coherent in vitro data sets. It is like "comparing apples and oranges", as the common idiom states. To my knowledge, there are no published in vitro studies that would show, for example, that the minimum diastolic potential (MDP) would be more negative in AL vs. VL hiPSC-CMs or engineered heart tissue (EHT) in coherent data sets. Please correct me, if I am wrong. The only coherent datasets that I am aware of, indicate the opposite. Check out Figure 6A in Lemme et al. (2018; https://doi.org/10.1016/j.stemcr.2018.10.008) regarding the MDP in AL vs. VL EHT. Those findings are in line with I_{K1} amplitudes – measured in co- herent in vitro conditions – that are about 7 vs. 14 pA/pF at -100 mV as reported by Schulz et al. (2023; https://doi.org/10.1161/circep.122.011602) and Horvath et al. (2018; https://doi.org/10.1016/j.stemcr.2018.01.012) for AL vs. VL hiPSC-CMs, respectively. The results that authors present are valid and insightful in the context of tissue heterogeneity but not in the context AL vs. VL hiPSC-CMs phenotype.

Answer. We appreciate the reviewer's thorough feedback and acknowledge the need to reduce the strength of our claims regarding the utility and physiological significance of our framework in the context of VL vs. AL hiPSC-CM phenotypes. In the revised manuscript, we ensured that our discussion reflects this adjustment. Below, we address the specific concerns raised about our comparison between VL and AL model variants: First, we would like to clarify that the results of Altomare et al. are indeed based on comparisons between pools of AL and VL hiPSC-CMs derived from the same cultures and protocols. These pools were subjected to DC techniques, and the MDP after the injection of I_{K1} does not show a significant difference between AL and VL phenotypes. This is an important consideration that we highlighted in order to moderate the physiological implications of our findings.

In our study, we did not employ the published model versions in which the difference in MDP between AL and VL is substantial. Instead, we utilized a version with modified g_{K1} values to represent a more immature state of hiPSC-CMs, as illustrated by the solid lines in Figure 3 of our manuscript. This adjustment aligns better with the experimental conditions under which immature hiPSC-CMs are typically observed.

Additionally, following the reviewer's suggestion, we revisited Figure 8 of Lemme et al. (2018; https://doi.org/10.1016/j.stemcr.2018.10.008). Specifically, we analyzed

the upper panel, which depicts engineered heart tissue (EHT) treated with or without retinoic acid. This scenario appears more relevant to our study's context of cells in MEA cultures. Importantly, this figure does not reveal a pronounced discrepancy in MDP between AL and VL phenotypes. Instead, when overlaying the data from Lemme et al. with our solid lines in Figure 3, we observe a similar profile, supporting the validity of our parameterization.

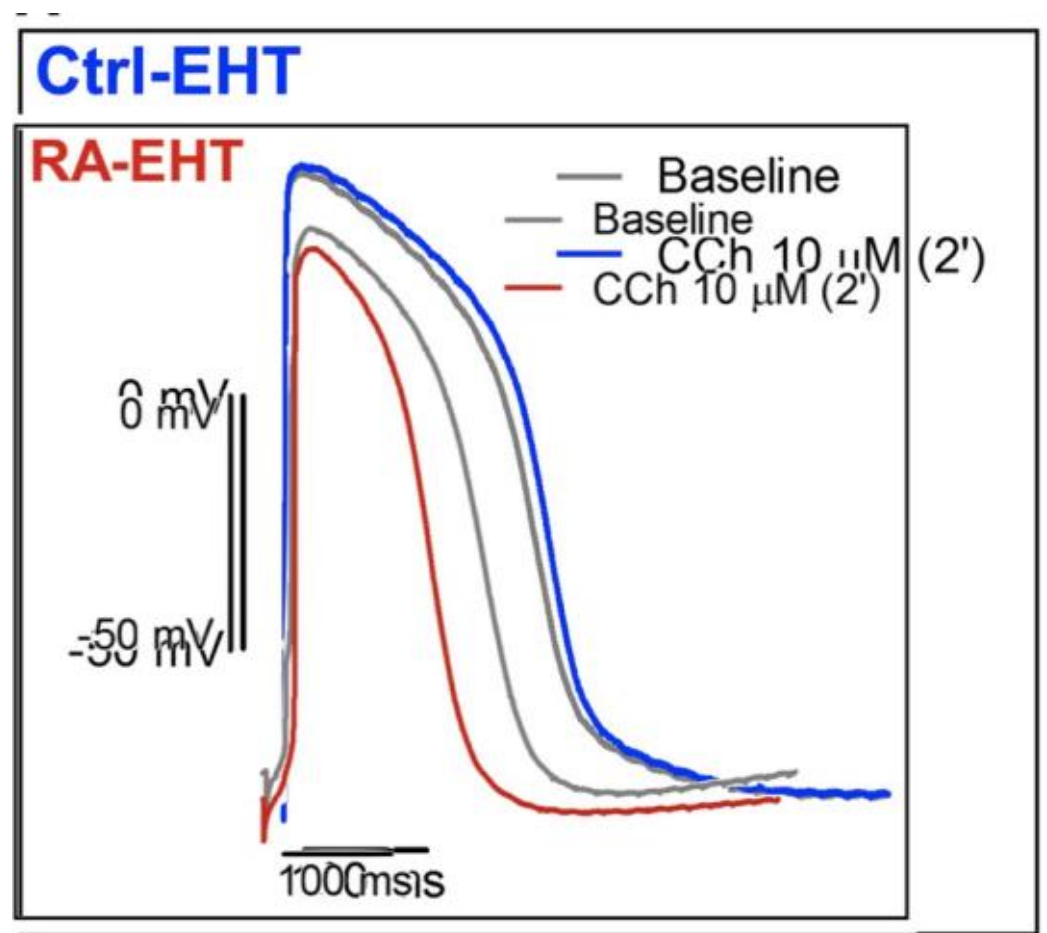

In light of these findings, we acknowledge the limitations of comparing VL and AL hiPSC-CM phenotypes without fully coherent in-vitro datasets. Therefore, we revised the manuscript, we moved the presentation on ionic model for chamber-specific hiPSC-CMs in the Discussion section (page 28), in order to emphasize that our results are more relevant to understanding tissue heterogeneity rather than providing definitive insights into the VL vs. AL hiPSC-CM phenotype.
We thank the reviewer for pointing out additional references that deepen our discus- sion. We have incorporated them in the text (pages 28 - 29) in order to ensure that our manuscript provides a balanced and accurate representation of the physiological significance of our findings.

4) About the modelling of the Brugada Syndrome (BrS). While the authors have cleverly selected BrS as a disease case to investigate with their frame- works, they are hampering potential significance of their findings by selecting a generic, "top-down" way to implement the BrS variants of the in silico cell models. There are plenty of hiPSC-based in vitro studies that report the changes in I_{Na} properties. For example, a search "brugada hiPSC patch- clamp" gives 18 results in PubMed. I would urge the authors to select a specific BrS-causing mutation and to implement a corresponding "bottom- up" BrS variants of the cell models. The simulations results obtained with that approach are more physiologically relevant and potentially much more insightful. That is because – at least to my knowledge – the weaker I_{Na} in BrS is commonly a combination of decreased sodium channel expression and shifted voltage dependencies of activation & inactivation (and in some mutations, also recovery kinetics) of I_{Na}. Those alterations together with the

tissue heterogeneity, which authors can account for in their framework, would potentially result in much more complex changes in electrical function of the virtual tissue.

Answer. We appreciate the suggestion to enhance the physiological relevance of our simulations by incorporating specific BrS-causing mutations. In response, we conducted an extensive literature review with the goal of identifying experimental data reporting changes in ionic current properties, particularly I_{Na}, associated with specific mutations relevant to Brugada Syndrome (BrS). Specifically, we focused on alterations in the activation and inactivation functions of sodium channels caused by SCN5A mutations. However, despite our efforts, we were unable to find sufficient experimental data that directly provided these details for I_{Na} in SCN5A-related BrS. During our review, we discovered experimental data describing the activation and inactivation properties of the L-type calcium current (I_{CaL}), but these were associated with mutations in the CACNA1C gene, which are also implicated in BrS (referred to as BrS2). Recognizing the potential significance of these findings, we decided to incorporate the CACNA1C mutation into our study, extending our analysis to include I_{CaL} dynamics in BrS2. This allowed us to adopt the bottom-up approach suggested by the referee, thereby enriching the physiological relevance of our simulations.
The addition of BrS2 cases aligns with the referee's recommendation for a bottom-up approach. For BrS2, we modeled the specific alterations in the L-type calcium current (I_{CaL}) due to CACNA1C mutations based on the data extracted from patch-clamp experiments. These mutations are associated with reduced calcium channel functionality, as highlighted in studies such as [Antzelevitch et al., 2007]. This allowed us to capture the differential electrophysiological profiles of BrS1 and BrS2, enriching the physiological insights offered by our computational framework.
We have updated the manuscript to reflect these improvements. Key additions include *(i)* a detailed explanation of the pathophysiological basis of BrS linked to both SCN5A (BrS1) and CACNA1C (BrS2) mutations; *(ii)* simulation results for BrS2 based on the bottom-up approach, including altered I_{CaL} properties, highlighting the interplay between sodium and calcium currents in arrhythmogenesis; *(iii)* discussion of the clinical and electrophysiological implications of our findings, emphasizing how the expanded analysis offers a more comprehensive understanding of BrS mechanisms.

5) On page #18, the authors state: "Conversely, by reducing late-phase calcium entry, mexitiline. . . ". However, Figure A15 does not indicate that. In the panel 2 nd from the top on the left (Paci20 model), the amplitude of I_{CaL} appears to be larger during the late phase of the action potential. In the same Figure, corresponding panel on the right, it is not possible to compare the traces of control vs. mexitiline for the Botti24 model, because the figure panel appears to include some other current than I_{CaL} (perhaps I_{Na}?). The 5th panel from the top on the right (i.e., the "nifedipine" panel) in that same Figure appears to include actual I_{CaL} traces. And they do not correspond with the panel 2nd from the top on the right. Please correct the Figure. Also, consider including an additional panel that zooms in the differences of the I_{CaL} and/or calculating the integral of I_{CaL}. That is, if the authors make some statements in the text about calcium entry changing, that the difference should be quantified and/or clearly visible in the Figure.

Answer. Thank you for noticing the issue with the figure; indeed, we mistakenly plotted I_{Na}

instead of I_{CaL}, as the referee correctly assumed. The figure has now been corrected. We also acknowledge that the sentence "by reducing the late-phase calcium entry" can be misleading. We did not intend to suggest that I_{CaL} specifically affects the late phase of the action potential. Rather, we merely wanted to reference the name of the current, as I_{CaL} denotes the L-type calcium channel passing inward Calcium current. However, we made an error when rephrasing the sentence. Thus, we have removed that reference to avoid confusion.

Minor points to consider and fix:

- **Page #2: What is this comma before the citation? That is not standard formatting, right? For example: "discovery, [4]."**

Answer. Thank you for noticing the discrepancy between the manuscript and the journal's guidelines. We have changed the citations format in the text, modifying it according to the journal guidelines.

- **Page #2: "insights into long-term maturation"; reference?**

Answer. We have added the reference to Clements, M., Thomas, N., 2014b. High-throughput multi-parameter profiling of electrophysiological drug effects in human embryonic stem cell derived cardiomyocytes using multi-electrode arrays. T*oxicological sciences: an official journal of the Society of Toxicology* 140, 445-461. We notice that the text has been moved to the Discussion section.

- **Page #2: "due to variability"; variability of what exactly?**

Answer. Thank you for your doubt, which allowed us to correct and improve the text as follows: "The variability of FPs arises from several factors, including differences in cell density, culture conditions, electrode placement, and the inherent heterogeneity of hiPSC- CM preparations. These factors influence the amplitude, duration, and morphology of the recorded signals, making it difficult to standardize and interpret results across experiments." We notice that the text has been moved to the Discussion section.

- **Figure 2: "spontaneously beating" instead of "spontaneous beating"**

Answer. Thank you for noticing. We changed "spontaneous beating" to the more appropriate "spontaneously beating" in the figure.

- **Page #6: "…AP triangular shape"; reference?**

We have added at pages 2 and 28 the citations Chapotte-Baldacci, C.A., Pierre, M., Djemai, M., Chahine, M., 2024. Characterizing ionic currents of atrial-like and ventricular- like hiPSC-cardiomyocytes. Biophysical Journal 123. doi:10.1016/j.bpj.2023.11.2341. and Chapotte-Baldacci, C.A., Pierre, M., Djemai, M., Pouliot, V., Chahine, M., 2023. Bio- physical properties of nav1.5 channels from atrial-like and ventricular-like cardiomy- ocytes derived from human induced pluripotent stem cells. Scientific Reports 13. doi:10.1038/s41598-023-47310-6.

- **Page #6: I think proper English phrase is "According to" or "In accordance with" instead of "According with"?**

Answer. We agree and we modified the text according to the proper English phrase "In accordance with". The sentence is now: "To model the electrophysiological heterogeneity, we introduce both phenotypes in the same domain. In accordance with Altomare et al. (2023), 30% of recorded cells in a standard dish can be defined as AL CM."

- **Page #7: Very long sentence that is difficult to follow. Please, rewrite as two separate sentences: "In Paci13 [20], the authors introduce AL and VL ionic models, highlighting that the primary distinctions lie in the conductances of certain channels and the dynamics of ICaL, which primarily contributes to the plateau phase; but it was based on recordings obtained from a mixture of nodal- ventricular and atrial–like hiPSC–CMs and the reproduce the phenotypical heterogeneity different scalings were considered, instead of phenotype–specific currents."**

Answer. Thank you for the valuable suggestion. We have changed the text as follows. "In Paci13 (Paci et al., 2013), the authors introduce AL and VL ionic models, highlighting that the primary distinctions lie in the conductances of certain channels and the dynamics of I_{CaL}, which primarily contributes to the plateau phase. Although, Paci2013 model was based on recordings obtained from a mixture of nodal- ventricular and atrial–like hiPSC–CMs and the phenotypical heterogeneity was reproduced considering different scalings, instead of phenotype–specific currents."

- **Page #7: Please write "real-time"; instead of "real time"?**

Answer. Thank you for the suggestion, we have changed the text as: "Among the different maturation techniques, the dynamic clamp (DC) overcomes the immature electrical properties of hiPSC-CM through the injection of a virtual current in a real-time mode."

- **Figure 3: Please write "spontaneous" instead of "unpaced", for the sake of consistency.**

Answer. Thanks you, we have improved the legend following your suggestion.

- **Page #8: Should be "immature"; instead of "mature"?**

Answer. Thank you for the correction, we changed the text as follows. “The published model accurately reflects the AL AP under external stimulation and injection of additional I_{K1}; nevertheless, the immature condition showing spontaneous firing activity arises for lower values of the I_{K1} conductance (g_{K1}).”

- **Page #8: Please write “Thus, here” instead of “Thus”?**

Answer. Thank you, we improved the text as suggested: “Thus, here we considered a modified version of the published model.”

- **Page #9: Please write “which results in” instead of “we reach the”?**

Answer. We have changed the text as: “We choose σ^i = 0.01 mS/cm and σ^e = 0.1 mS/cm as suggested in Jæger et al. (2021a) and which results in conduction velocity equal to 4 cm/s matching Abbate et al. (2018).”

- **Page #9: Please write “4 cm/s matching [11].” instead of “4 cm/s provided by the study of [11].”?**

Answer. We have changed the text as: “We choose σ^i = 0.01 mS/cm and σ^e = 0.1 mS/cm as suggested in Jaeger et al. (2021) and which results in conduction velocity equal to 4 cm/s matching Abbate et al. (2018).”

- **Page #9: Please write “conduction” instead of “conduction velocity”?**

Answer. We have changed the text as: “These values are lower than those observed in human cardiac tissue, as the conduction in hiPSC-CMs is significantly slower compared to that in the human heart.”

- **Page #13: The cv definition should be in Methods?**

Answer. Yes, we have moved the conduction velocity definition to the Methods section (subsection 2.6).

- **Page #13, this appears borderline trivial: “Furthermore, an analysis of the Repolarization Times reveals that AL tissues tend to repolarize more quickly, requiring approximately 100 ms less with respect to the homogeneous VL tissue to return to the resting potential. This finding aligns with the triangular shape of the AL AP, which characterizes their faster recovery dynamics compared to other tissue types.” I mean, what else could one expect, as AL cells have shorter APs than VL cells. So, maybe rephrase this to something like: “As expected based on the shorter APs in AL cells, . . . ”?**

Answer. We have rephrased the sentences in the following way: “As expected due to the shorter action potential duration in AL cells, the Repolarization Times analysis shows that AL tissues repolarize earlier, requiring about 100 ms less than homogeneous VL tissue to return to the resting potential. This behavior reflects the triangular shape of the AL action potential, highlighting their faster recovery dynamics compared to other tissue types.”

- **Page #15: Please write "hiPSC-CM-based in vitro models" instead of "to in-vitro models proposed in this study, based on hiPSC-CMs"?**

Answer. We have corrected the sentence as: "However, these insights may not fully apply hiPSC-CM-based *in-silico* models, where the heterogeneous differentiation of the cells makes it challenging to map specific substrates to anatomical regions, such as the right ventricle."

- **Page #15: Something important missing here: "protocol, which leads to the 30"?**

Answer. Thank you for noticing this omission. We restored the text as: "Following the approach proposed by Hoogendijk et al. (2011), where ST-segment elevation in BrS is linked to reduced sodium channel (g_{Na}) and L-type calcium channel (g_{CaL}) conductances alongside increased potassium channel (g_{to}) conductance, we simulate these ionic alterations using the Paci2020 VL hiPSC-CM model. In our approach, we adjust g_{Na}, g_{CaL}, and g_{to} under the assumption that all cells within the simulated tissue region exhibit characteristics of BrS. Furthermore, since our interest is focused on a ventricular-specific disorder, we consider the standard differentiation protocol, which leads to the 30 % of VL hiPSC-CMs in the culture."

- **Figure 8: What does "ionic AP" mean, in contrast to just an "AP"? Please write simply "AP" instead.**

Answer. We have corrected the caption, thanks.

- **Page #16: Something missing here: "and set to 110"?**

Answer. Thank you for noticing this omission. We have changed the text as: "g_{to} is increased and set to 110 % of its normal value. This observation is quantitatively confirmed by calculating the average conduction velocity using Eq. (20), which yields a value of 3.59 cm/s in the tissue affected by BrS."

- **Page #17: Please write "blockers" instead of "blocker"?**

Answer. We have eliminated the typo and restructured several paragraphs on current blockers' effects in the Results section (subsection 2.8).

- **Page #22: Please consider rewriting the 3rd paragraph of Discussion, with a bit less repetition regarding the EMI model.**

Answer. As requested, we rephrased the paragraph as follows: "Finally, our model assumes that the intracellular (I), cell membrane (M), and extracellular (E) spaces are uniformly distributed throughout the simulated domain. While this simplification has been effective in computational cardiology for millimeter-scale resolutions over decades, it becomes less accurate at the micrometer scale of individual cells. At this level, more detailed approaches like the Extracellular-Membrane-Intracellular (EMI) model, which explicitly separates the computational domains for the E, M, and I spaces, offer improved

precision (Béecue et al., 2017; Jæger and Tveito, 2022; Tveito et al., 2017). However, despite their accuracy at subcellular scales, these methods are computationally intensive. For this study, the bidomain model remains the preferred choice due to its efficiency in simulating larger tissue geometries."

- **Page #22: Please write "VL cells" and "AL cells" instead of "VL stem cells" and "AL stem cells". After the CMs have been derived and differentiated from stem cells, they are no longer stem cells, right?**

Answer. Thank you for addressing this point: we agree that the CMs differentiated from stem cells are no longer stem cells. We have modified the text as follows. "We accounted for the heterogeneity in hiPSC-CMs phenotypes by adopting the Paci20 ionic model, (Paci et al., 2020), which captures the electrophysiological properties of VL hiPSC cells. Additionally, we incorporated the recently published Botti24 model for AL hiPSC cells, (Botti et al., 2024), extending the utility of Paci's ionic modeling framework to encompass various phenotypes."

- **Page #24: It is not clear, how the tool will potentially benefit "regenerative medicine". So, maybe it is more reasonable to make statements on the pharmacological aspects only?**

Answer. We accepted your suggestion; it is indeed premature to discuss regenerative medicine at this stage. Therefore, we have decided to remove it.

Dear Dr Botti,

Re: JP-RP-2025-287276R1 "In-silico Modeling of Multi-Electrode Arrays for Enhancing Cardiac Drug Testing on hiPSC-CMs Heterogeneous Tissues" by Sofia Botti, Rolf Krause, and Luca Franco Pavarino

Thank you for submitting your manuscript to The Journal of Physiology. It has been assessed by a Reviewing Editor and by 2 expert referees and we are pleased to tell you that it is acceptable for publication following satisfactory revision.

The referee reports are copied at the end of this email.

Please address all the points raised and incorporate all requested revisions or explain in your Response to Referees why a change has not been made. We hope you will find the comments helpful and that you will be able to return your revised manuscript within 4 weeks. If you require longer than this, please contact journal staff: jp@physoc.org.

Your revised manuscript should be submitted online using the link in your Author Tasks Link Not Available. This link is accessible via your account as Corresponding Author; it is not available to your co-authors. If this presents a problem, please contact journal staff (jp@physoc.org). Image files from the previous version are retained on the system. Please ensure you replace or remove any files that are being revised.

If you do not wish to submit a revised version of your manuscript, you must inform our journal staff (jp@physoc.org) or reply to this email to request withdrawal. Please note that a manuscript must be formally withdrawn from the peer review process at one journal before it may be submitted to another journal.

TRANSPARENT PEER REVIEW POLICY: To improve the transparency of its peer review process The Journal of Physiology publishes online, as supporting information, the peer review history of all articles accepted for publication. Readers will have access to decision letters, including Editors' comments and referee reports, for each version of the manuscript, as well as any author responses to peer review comments. Referees can decide whether or not they wish to be named on the peer review history document.

ABSTRACT FIGURES: Authors are expected to use The Journal's premium BioRender account to create/redraw their Abstract Figures. Information on how to access this account is here:
https://physoc.onlinelibrary.wiley.com/journal/14697793/biorender-access.

This will enable Authors to create and download high-resolution figures. If authors have used the free BioRender service, they can use the instructions provided in the link above to download a high-resolution version suitable for publication.
The link provided should only be used for the purposes of this submission. Authors will be charged for figures created on this account if they are not related to this manuscript submission.

LANGUAGE EDITING AND SUPPORT FOR PUBLICATION: If you would like help with English language editing, or other article preparation support, Wiley Editing Services offers expert help, including English Language Editing, as well as translation, manuscript formatting, and figure formatting at www.wileyauthors.com/eeo/preparation. You can also find resources for Preparing Your Article for general guidance about writing and preparing your manuscript at www.wileyauthors.com/eeo/prepresources.

RIGOUR AND REPRODUCIBILITY: We recommend authors consult The Journal's Rigour and Reproducibility requirements to ensure that their manuscript is suitable for publication: https://physoc.onlinelibrary.wiley.com/pb-assets/hub-assets/physoc/documents/TJP-Rigour-and-Reproducibility-Requirements.pdf

REVISION CHECKLIST:
Check that your Methods section conforms to journal policy: https://jp.msubmit.net/cgi-bin/main.plex?form_type=display_requirements#methods

Check that data presented conforms to the statistics policy: https://jp.msubmit.net/cgi-bin/main.plex?form_type=display_requirements#statistics

Upload a full Response to Referees file. To create your 'Response to Referees' copy all the reports, including any comments from the Senior and Reviewing Editors, into a Microsoft Word, or similar, file and respond to each point, using font or background colour to distinguish comments and responses and upload as the required file type.

Please upload two versions of your manuscript text: one with all relevant changes highlighted and one clean version with no changes tracked. The manuscript file should include all tables and figure legends, but each figure/graph should be uploaded as separate, high-resolution files. The journal is now integrated with Wiley's Image Checking service. For further details, see: https://www.wiley.com/en-us/network/publishing/research-publishing/trending-stories/upholding-image-integrity-wileys-image-screening-service

You may also upload:

- 'Potential Cover Art' for consideration as the issue's cover image
- Appropriate Supporting Information (Video, audio or data set: see https://jp.msubmit.net/cgi-bin/main.plex?form_type=display_requirements#supp).

We look forward to receiving your revised submission.

If you have any queries, please reply to this email and we will be pleased to advise.

Yours sincerely,

Natalia Trayanova
Senior Editor
The Journal of Physiology

EDITOR COMMENTS

Reviewing Editor:

There are still 4 minor points to address (1 from Ref1, 3 from Ref3). Please provide suitable changes and checks in the text before publication.

REFEREE COMMENTS

Referee #1:

The authors have addressed all of my questions and concerns consistently, robustly, and thoroughly. I would like to thank them for their constructive approach in responding to my revision.

As a very minor comment, I would recommend the authors to check the domain names assigned to Dirichlet and Neumann boundary conditions at page 6, since they seem to be inverted.

Referee #2:

Overall, the authors have done an excellent job in rewriting and restructuring the text, as well as reformatting and correcting the figures, in accordance with the concerns that Reviewer #1 and I raised in 1st review round.

I have one remaining major concern:

Reading through the rewritten and restructured Discussion section, it becomes apparent that the subsections "Impact of tissue heterogeneity and Brugada Syndrome" and "Drugs effects on heterogeneous MEA recordings" are detached from the rest of the manuscript. That is, the authors do not discuss their own findings in these subsections. Please, try to tie in your results with published studies that you are discussing.

Two minor notes:

1) Except for Figures 9 and 10, the Figures have widely variable font-sizes for the legends, panel labels, axis labels, and texts. That makes the Figures difficult and cumbersome to read. Please, agree on a common scheme and use that in all the figures. For example, something like: font-size 12 for the panel labels and 9 for everything else (when the Figures are scaled to their final/print size).

2) The authors write on page #32: "Notably, the ionic term in our bidomain model can be readily adapted to other established electrophysiological models of hiPSC-CMs, such as those by Koivumäki et al. (2018) and Kernik et al. (2019). Detailed comparative analyses of these models have been previously discussed, as referenced in recent reviews (Wilhelms et al., 2013; Vagos et al., 2018; Trayanova et al., 2024; Trancuccio, 2023)." There seems to be an internal contradiction in the logic of these references. That is, the Wilhelms et al. and Vagos et al. papers were published in 2013 and 2019, so it could not have analysed the models published in 2018 and 2019. Please, remove the Wilhelms et al. and Vagos et al. papers and cite instead this one that actually compares the aforementioned models: Paci et al. (2021). Comparison of the Simulated Response of Three in Silico Human Stem Cell-Derived Cardiomyocytes Models and in Vitro Data Under 15 Drug Actions. Front. Pharmacol. 12. doi: 10.3389/fphar.2021.604713.

END OF COMMENTS

March 17, 2025

Dear Editors of The Journal of Physiology,

We would like to sincerely thank you for your positive feedback on our manuscript, "*In-silico* Modeling of Multi-Electrode Arrays for Enhancing Cardiac Drug Testing on hiPSC-CMs Heterogeneous Tissues", coauthored by Sofia Botti, Luca F. Pavarino, and Rolf Krause, submitted for publication in *The Journal of Physiology*.

We also extend our gratitude to the reviewers for their constructive feedback and for their positive assessment of our work. Their comments have been invaluable in refining the manuscript, and we have carefully addressed all minor revisions requested.

Below, we provide a detailed response to each comment, outlining the corresponding changes made to the text. The revised sections are highlighted in red color in the manuscript for clarity.

We appreciate your time and consideration and look forward to hearing from you soon.

Sincerely,

Sofia Botti
Luca F. Pavarino
Rolf Krause

Referee Report (# 1)

The authors have addressed all of my questions and concerns consistently, robustly, and thoroughly. I would like to thank them for their constructive approach in responding to my revision.

As a very minor comment, I would recommend the authors to check the domain names assigned to Dirichlet and Neumann boundary conditions at page 6, since they seem to be inverted.

Answer. Thank you for raising these important points. They were inadvertently inverted when rephrasing the system into sentences. We have now corrected them as follows.

We also impose the following boundary conditions, where **n** is the outward pointing normal:
(i) homogeneous Neumann boundary conditions associated with the no flux assumption of the intracellular potential on the external surface $\partial\Omega$: $\mathrm{D}_i\nabla(v+u_e)\cdot\mathbf{n}=0$ on $\partial\Omega\times(0,T)$;
(ii) homogeneous Dirichlet boundary conditions on the three edges connected to the ground: $u_e\cdot\mathbf{n}=0$ on $\partial\Omega_D\times(0,T)$;
(iii) Neumann no-flux boundary condition for the remaining edge: $\mathrm{D}_e\nabla u_e\cdot\mathbf{n}=0$ on $\partial\Omega_N\times(0,T)$.

Referee Report (# 2)

Overall, the authors have done an excellent job in rewriting and restructuring the text, as well as reformatting and correcting the figures, in accordance with the concerns that Reviewer #1 and I raised in 1st review round.

I have one remaining major concern:

Reading through the rewritten and restructured Discussion section, it becomes apparent that the subsections ”Impact of tissue heterogeneity and Brugada Syndrome” and ”Drugs effects on heterogeneous MEA recordings” are detached from the rest of the manuscript. That is, the authors do not discuss their own findings in these subsections. Please, try to tie in your results with published studies that you are discussing.

Answer. Thank you for your insightful comments regarding the discussion of tissue heterogeneity and drug effects. We appreciate your feedback and have revised the manuscript to better integrate our results with published studies. Below, we summarize the key modifications made in response to your concerns.

Impact of Tissue Heterogeneity and Brugada Syndrome

We have strengthened the connection between our findings and prior literature by explicitly discussing how our results align with, contrast, or extend published studies. Specifically:

• We now emphasize that the observed increase in conduction velocity with AL hiPSC-CM proportion is consistent with heterogeneous tissue behavior reported in prior studies (Clark et al., 2024).

• We address the apparent discrepancy with O’Brien et al. (2022) and Goldfracht et al. (2020) by clarifying the fundamental structural differences between MEA syncytia and engineered heart tissues (EHT). These differences likely account for variations in conduction velocity, and we now explicitly discuss how cellular clustering and fiber alignment may influence this parameter.

• To further support our observations, we reference Riedel et al. (2014), which demonstrates how tissue heterogeneity impacts conduction and repolarization patterns in MEA recordings. We have now explicitly tied our results to their findings, particularly regarding the irregularity of APD isochrones in heterogeneous tissues.

Drug Effects on Heterogeneous MEA Recordings

We have revised this section to better integrate our findings with existing literature. Key improvements include:

• Directly linking our computational results to prior experimental and in silico studies on Mexiletine (Matsa et al., 2011), Dofetilide (Hancox et al., 2008; Zhao and Li, 2019), Nifedipine (Hayashi, 2000), and E-4031 (Grandi et al., 2010).

• Emphasizing that our simulations reproduce known drug-induced electrophysiological effects, such as Dofetilide's pro-arrhythmic risk via early afterdepolarizations (EADs) and E-4031's impact on repolarization reserve.

• Referencing previous multiscale modeling studies (Passini et al., 2017) to highlight the reliability of our approach in capturing MEA-acquired signals and drug response trends.

These revisions ensure that our discussion is fully anchored in the context of published studies while reinforcing the novelty and significance of our findings. We believe these improvements address your concerns and enhance the manuscript's clarity and impact.

Two minor notes:

1) Except for Figures 9 and 10, the Figures have widely variable font-sizes for the legends, panel labels, axis labels, and texts. That makes the Figures difficult and cumbersome to read. Please, agree on a common scheme and use that in all the figures. For example, something like: font-size 12 for the panel labels and 9 for everything else (when the Figures are scaled to their final/print size).

Answer. Thank you for your valuable feedback regarding figure consistency. We carefully reviewed all figures to ensure a uniform font size for legends, panel labels, axis labels, and text. Below, we detail the specific modifications made:

Figure 1: We have increased the size of the panel labels (a) and (b) to enhance clarity. Additionally, we adjusted the font sizes in subfigure (b) to improve readability.

Old:

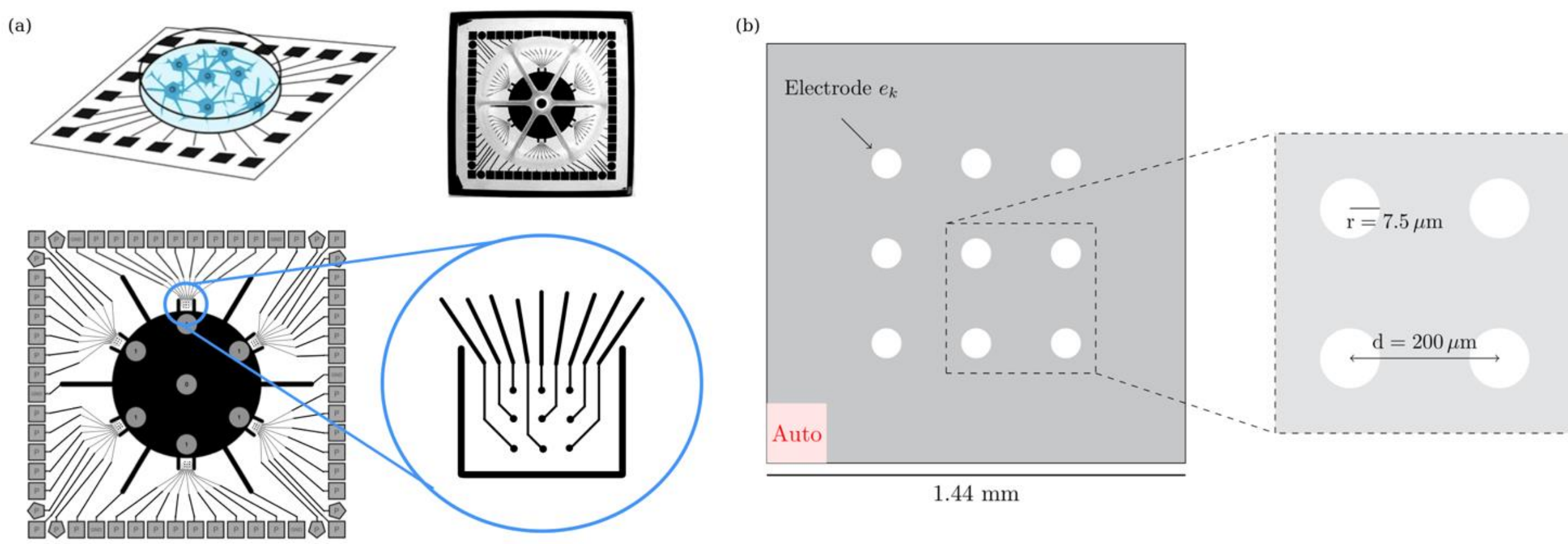

New:

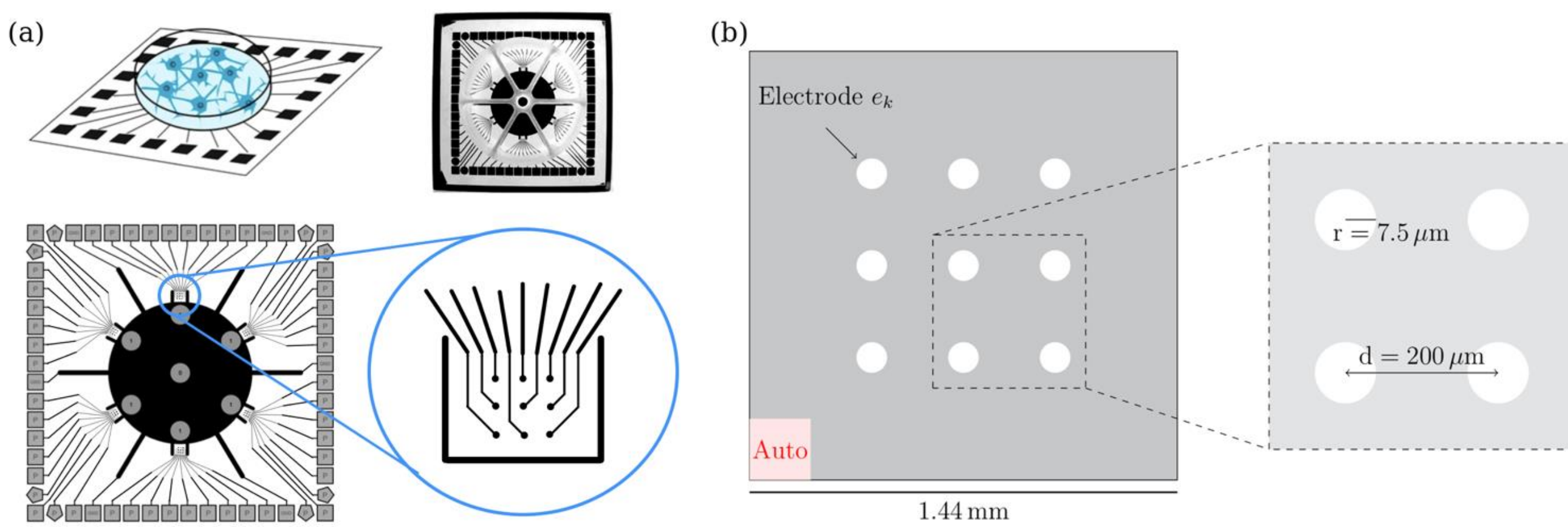

Figures 2 and 3: These figures were generated using LaTeX, and their font sizes are already aligned with the main text. Given that they are naturally well-integrated into the manuscript, we did not find it necessary to modify them. However, if you have any specific concerns, we would be happy to make further refinements.

Figures 4, 5, 6, and 7: We have updated these figures to ensure a uniform font size and consistent formatting. As an illustrative example, Figure 4 was modified as follows:

Old:

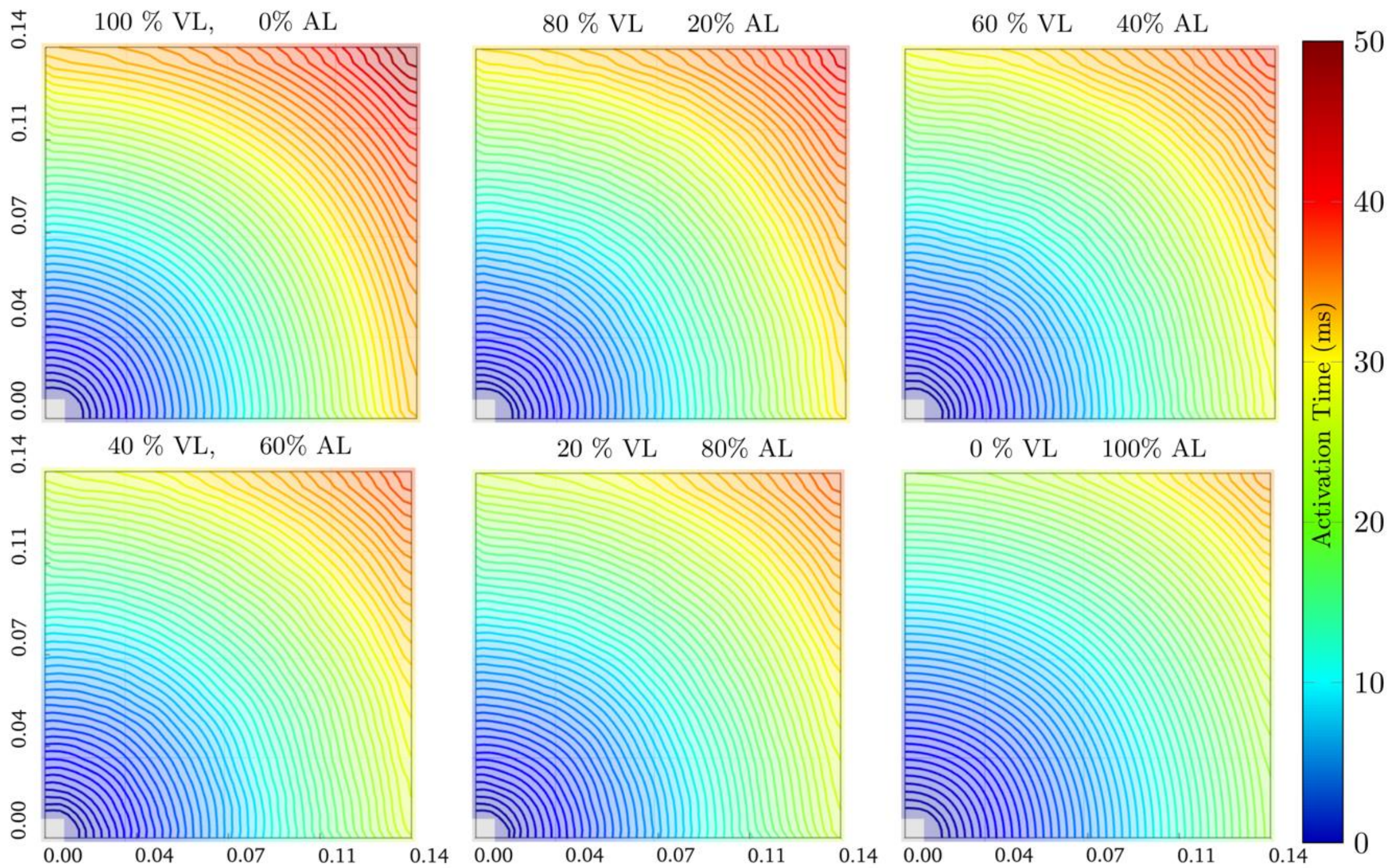

New:

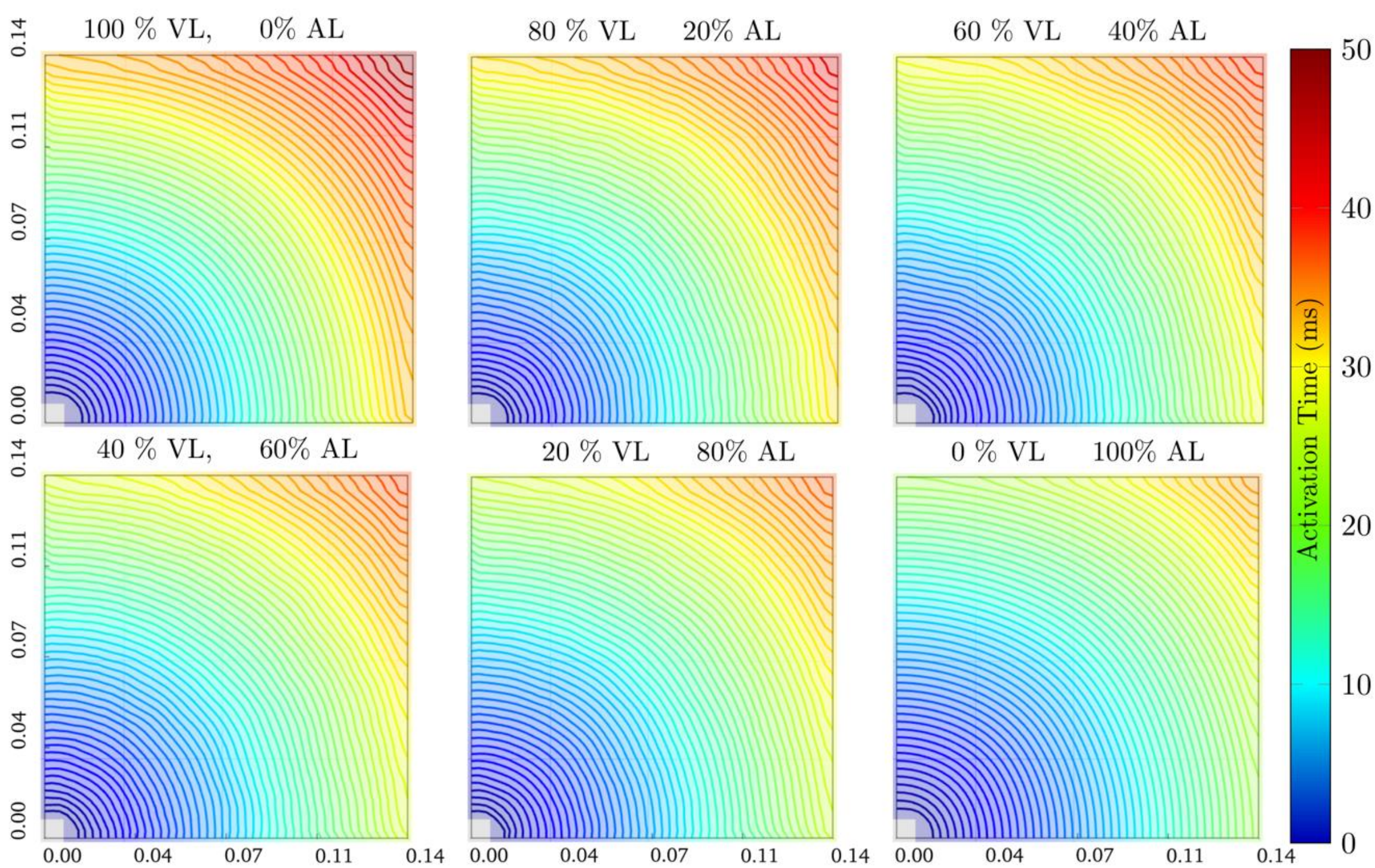

Figure 8: We increased the font sizes of the tick labels and enhanced the image quality to improve readability.

Old:

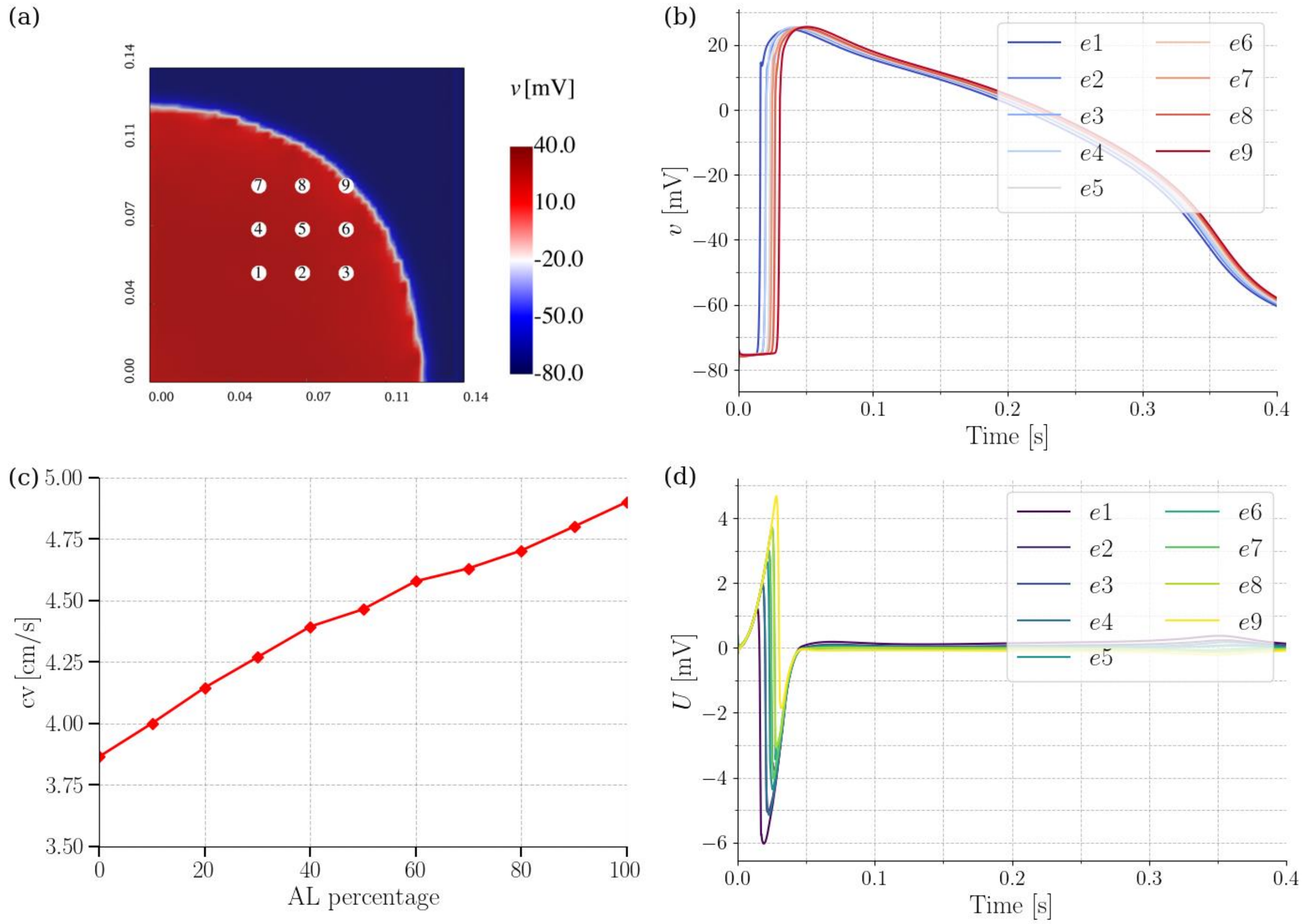

New:

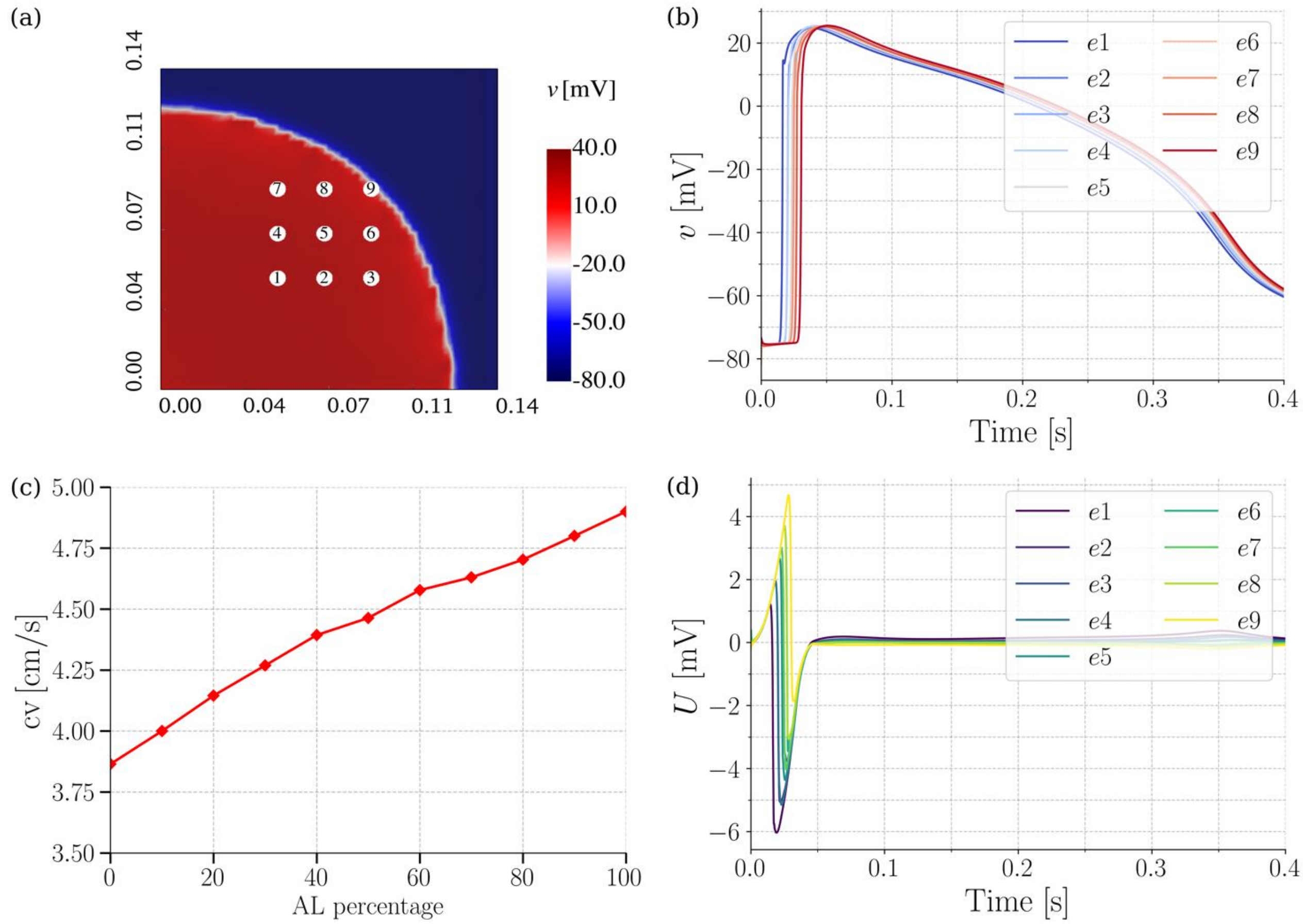

(a)
v [mV]
40.0
10.0
-20.0
-50.0
-80.0
0.00
0.04
0.07
0.11
0.14
1 2 3 4 5 6 7 8 9
(b)
v [mV]
Time [s]
e1 e2 e3 e4 e5 e6 e7 e8 e9
(c)
cv [cm/s]
AL percentage
(d)
U [mV]
Time [s]
e1 e2 e3 e4 e5 e6 e7 e8 e9

Figure 11: This figure was adjusted according to the same criteria applied to Figures 4–7, ensuring consistent formatting and readability.

Old:

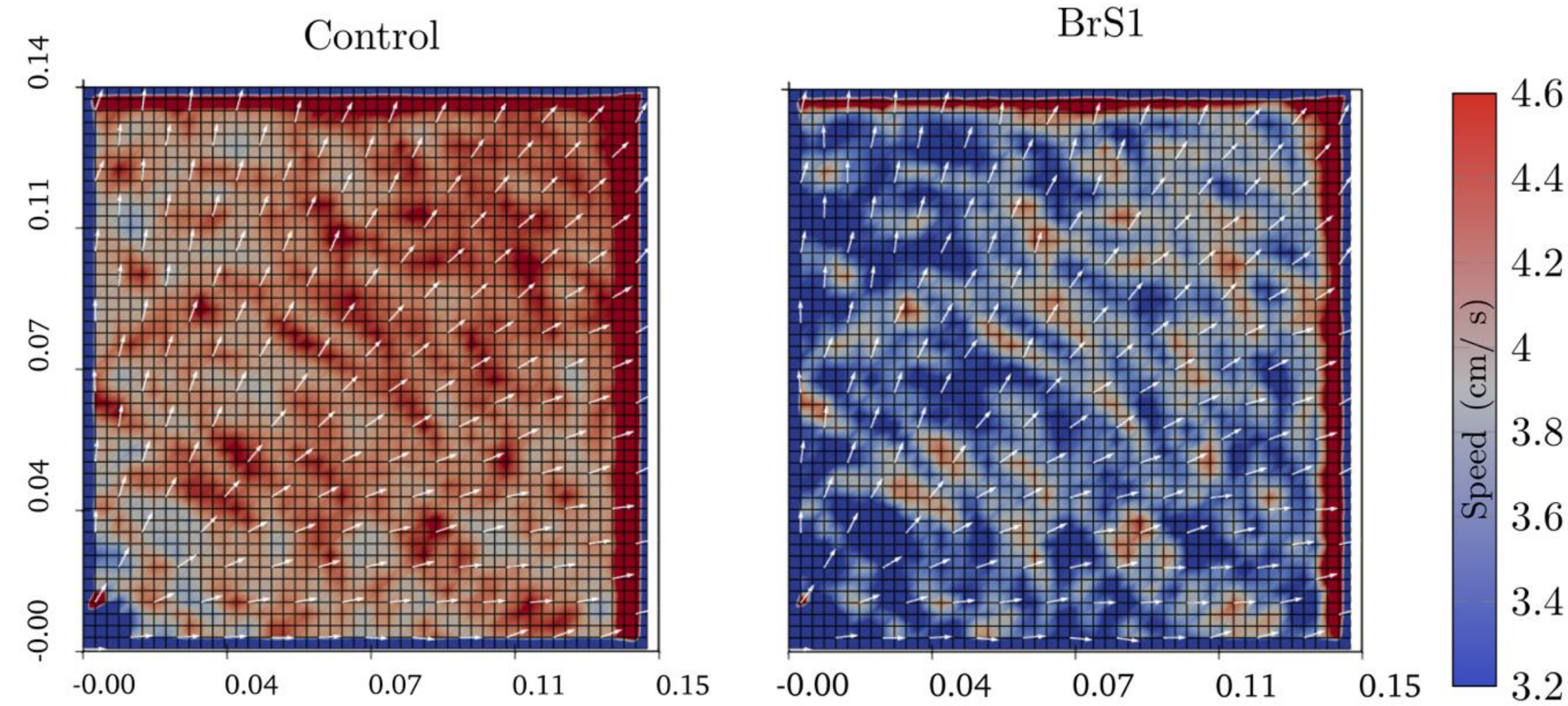

New:

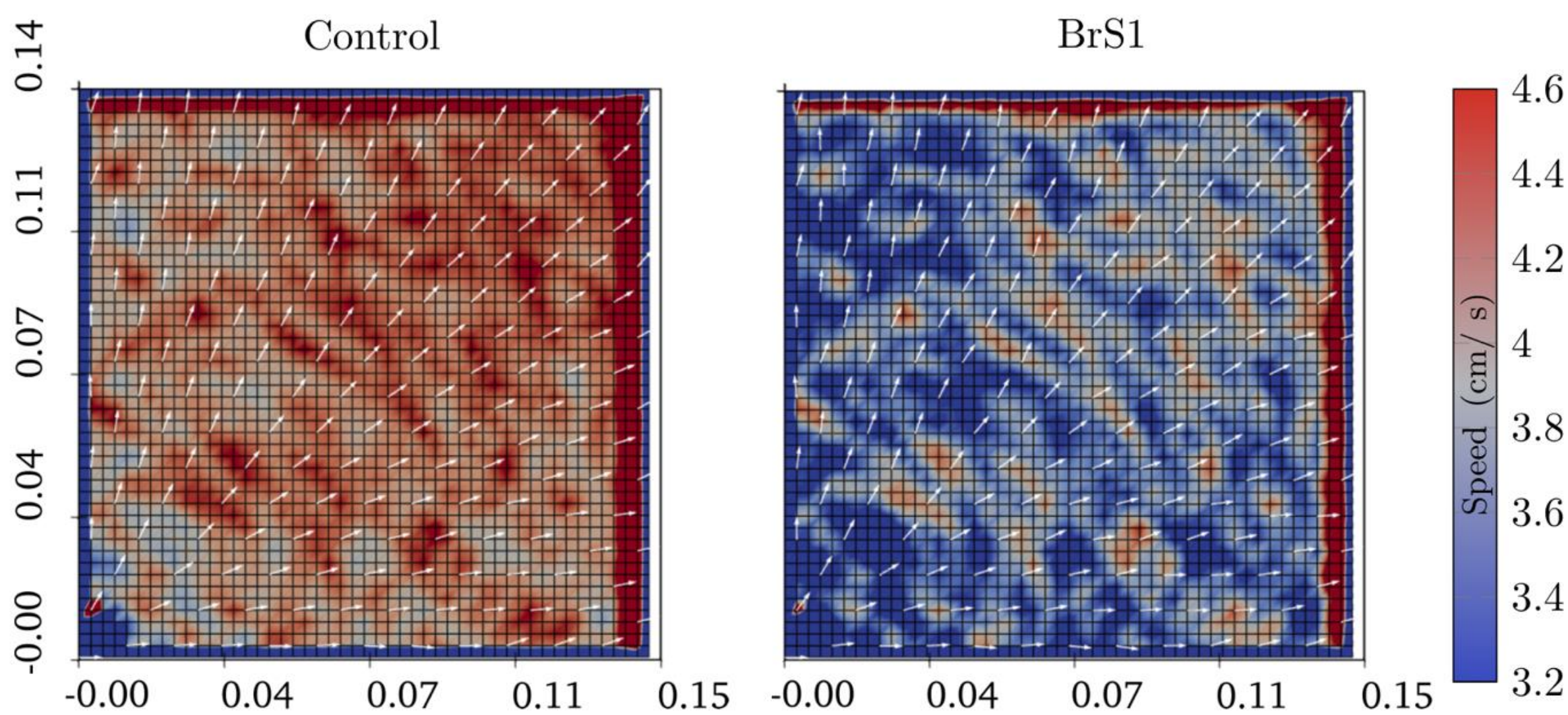

Figures 12–15: These figures contain screenshots from a published paper, making it challenging to modify the embedded text and tick labels directly. However, we carefully adjusted the tick marks where possible to ensure better consistency with the rest of the figures. Additionally, we noticed that Figures 14 and 15 contained drug names with disproportionately large font sizes, which we have corrected for better balance.

Old Figure 14:

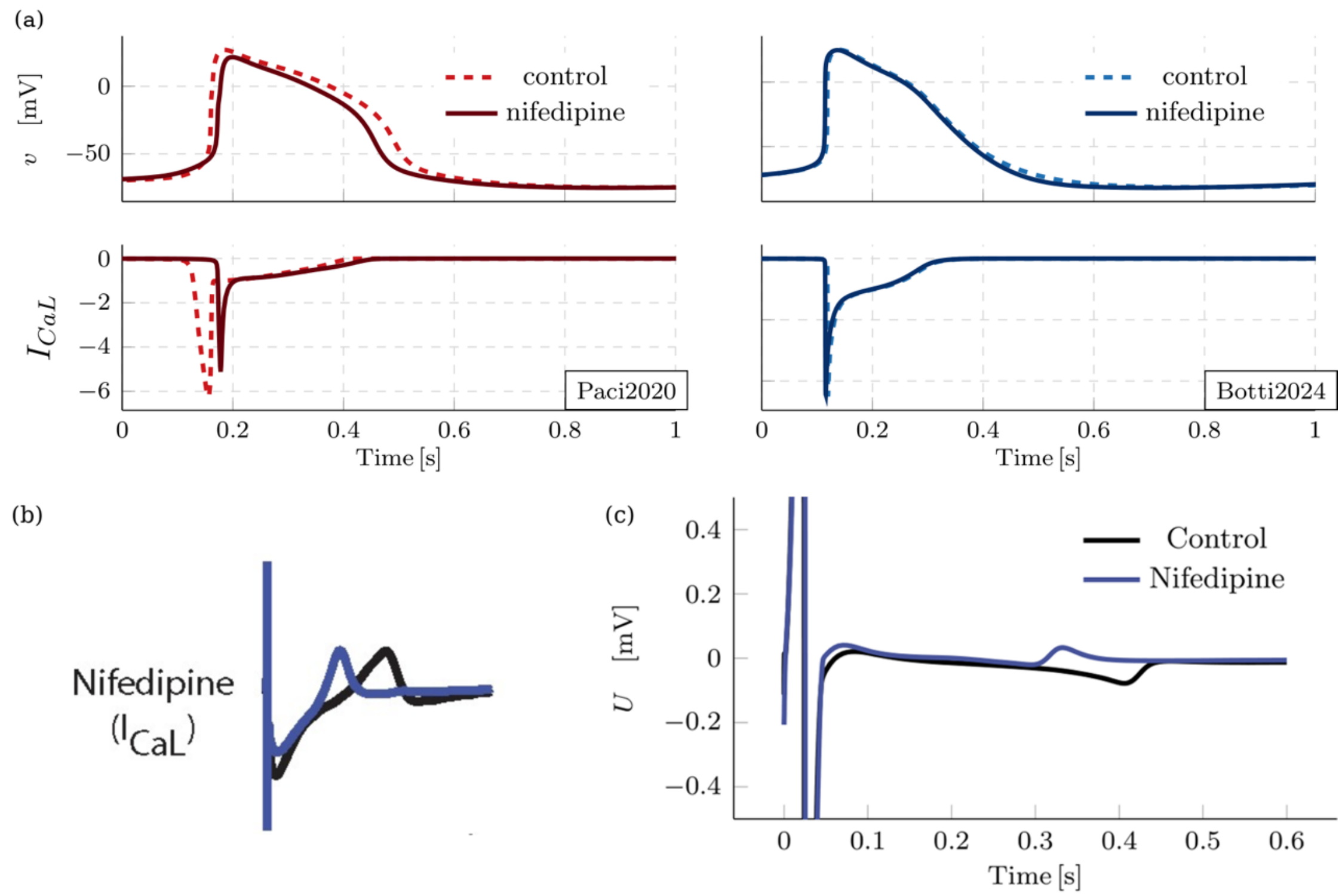

New Figure 14:

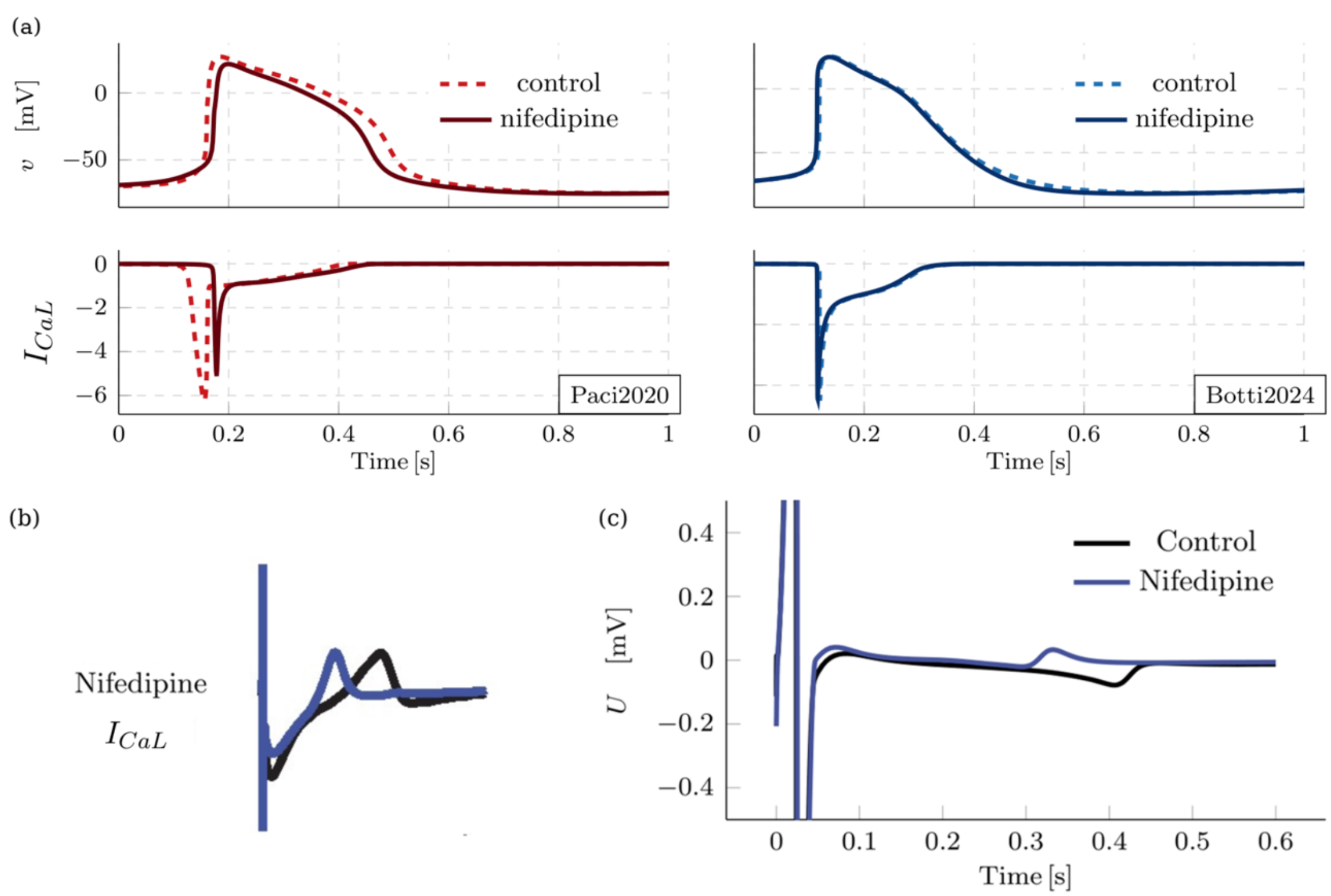

Old Figure 15:

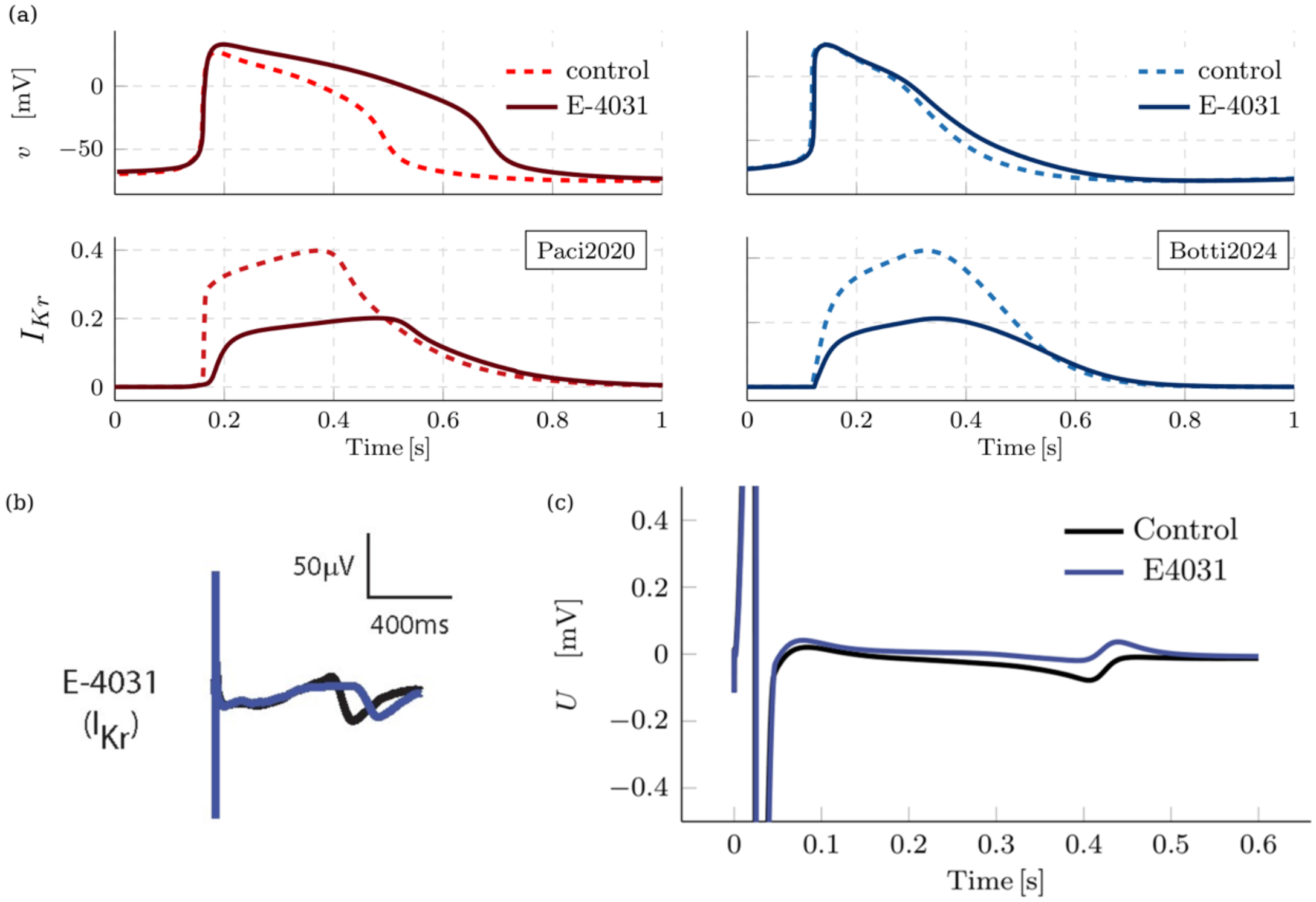

New Figure 15:

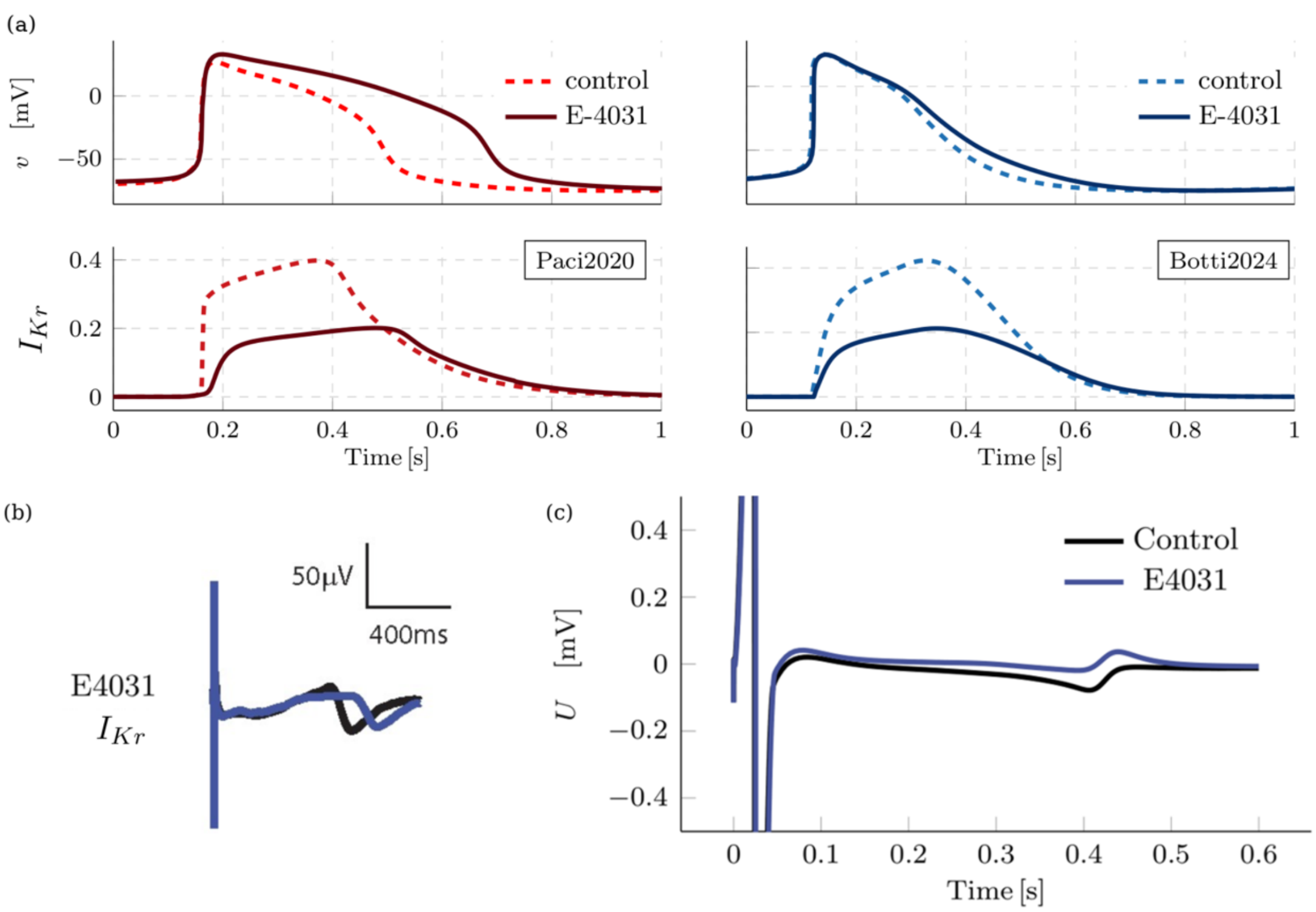

2) The authors write on page #32: “Notably, the ionic term in our bidomain model can be readily adapted to other established electrophysiological models of hiPSC-CMs, such as those by Koivum¨aki et al. (2018) and Kernik et al. (2019). Detailed comparative analyses of these models have been previously discussed, as referenced in recent reviews (Wilhelms et al., 2013; Vagos et al., 2018; Trayanova et al., 2024; Trancuccio, 2023).” There seems to be an internal contradiction in the logic of these references. That is, the Wilhelms et al. and Vagos et al. papers were published in 2013 and 2019, so it could not have analysed the models published in 2018 and 2019. Please, remove the Wilhelms et al. and Vagos et al. papers and cite instead this one that actually compares the aforementioned models: Paci et al. (2021). Comparison of the Simulated Response of Three in Silico Human Stem Cell-Derived Cardiomyocytes Models and in Vitro Data Under 15 Drug Actions. Front. Pharmacol. 12. doi: 10.3389/fphar.2021.604713.

Answer. We thank the reviewer for pointing out this inconsistency in our references. We have now removed the citations of Wilhelms et al. (2013) and Vagos et al. (2018), as they could not have analyzed the Koivum¨aki et al. (2018) and Kernik et al. (2019) models. Instead, we have cited Paci et al. (2021), which provides a direct comparison of these models, as suggested. This correction ensures that our references accurately support the statement regarding comparative analyses of electrophysiological models of hiPSC-CMs.

Dear Dr Botti,

Re: JP-RP-2025-287276R2 "In-silico Modeling of Multi-Electrode Arrays for Enhancing Cardiac Drug Testing on hiPSC-CMs Heterogeneous Tissues" by Sofia Botti, Rolf Krause, and Luca Franco Pavarino

We are pleased to tell you that your paper has been accepted for publication in The Journal of Physiology.

The last Word (or similar) version of the manuscript provided will be used by the Production Editor to prepare your proof. When this is ready you will receive an email containing a link to Wiley's Online Proofing System. The proof should be thoroughly checked and corrected as promptly as possible.

Authors should note that it is too late at this point to offer corrections prior to proofreading. Major corrections at proof stage, such as changes to figures, will be referred to the Editors for approval before they can be incorporated. Only minor changes, such as to style and consistency, should be made at proof stage. Changes that need to be made after proof stage will usually require a formal correction notice.

All queries at proof stage should be sent to: TJP@wiley.com.

Following copyediting and typesetting the accepted version will be published online.

Yours sincerely,

Natalia Trayanova
Senior Editor
The Journal of Physiology

IMPORTANT POINTS TO NOTE FOLLOWING YOUR PAPER'S ACCEPTANCE:

If you would like to receive our 'Research Roundup', a monthly newsletter highlighting the cutting-edge research published in The Physiological Society's family of journals (The Journal of Physiology, Experimental Physiology, Physiological Reports, The Journal of Nutritional Physiology and The Journal of Precision Medicine: Health and Disease), please click this link, fill in your name and email address and select 'Research Roundup':
https://www.physoc.org/journals-and-media/membernews

• TRANSPARENT PEER REVIEW POLICY: To improve the transparency of its peer review process, The Journal of Physiology publishes online as supporting information the peer review history of all articles accepted for publication. Readers will have access to decision letters, including Editors' comments and referee reports, for each version of the manuscript as well as any author responses to peer review comments. Referees can decide whether or not they wish to be named on the peer review history document.

• You can help your research get the attention it deserves! Check out Wiley's free Promotion Guide for best-practice recommendations for promoting your work at: www.wileyauthors.com/eeo/guide. You can learn more about Wiley Editing Services which offers professional video, design, and writing services to create shareable video abstracts, infographics, conference posters, lay summaries, and research news stories for your research at: www.wileyauthors.com/eeo/promotion.

• IMPORTANT NOTICE ABOUT OPEN ACCESS: To assist authors whose funding agencies mandate public access to published research findings sooner than 12 months after publication, The Journal of Physiology allows authors to pay an Open Access (OA) fee to have their papers made freely available immediately on publication.

The Corresponding Author will receive an email from Wiley with details on how to register or log-in to Wiley Authors Services where you will be able to place an order.

• You can check if your funder or institution has a Wiley Open Access Account here: https://authorservices.wiley.com/author-resources/Journal-Authors/licensing-and-open-access/open-access/author-compliance-tool.html.

EDITOR COMMENTS

Reviewing Editor:

No further comments.

REFEREE COMMENTS

Referee #2:

The authors have addressed all my concerns and suggestions thoroughly. Overall, I appreciate their thoughtful and consistent approach to the whole revision process.
